# Annexin A2 stabilizes the endoplasmic reticulum and actin cytoskeleton and influences the formation of reovirus factories

**DOI:** 10.1128/jvi.01389-25

**Published:** 2025-11-24

**Authors:** Raquel Tenorio, Gwen M. Taylor, Ana Cayuela, C. O. S. Sorzano, Isabel Fernández de Castro, Sara Y. Fernández-Sánchez, Alexa N. Roth, Xayathed Somoulay, Gavin S. Treadaway, Terence S. Dermody, Cristina Risco

**Affiliations:** 1 Cell Structure Laboratory, National Center for Biotechnology, CNB-CSIC, Madrid, Spain; 2 Department of Pediatrics, University of Pittsburgh School of Medicine 12317 , Pittsburgh, Pennsylvania, USA; 3 Institute of Infection, Inflammation and Immunity, UPMC Children’s Hospital of Pittsburgh, Pittsburgh, Pennsylvania, USA; 4 Microscopy Core Unit, Spanish National Cancer Research Centre-CNIO 16358, Madrid, Spain; 5 Biocomputing Unit, National Center for Biotechnology, CNB-CSIC, Madrid, Spain; 6 Department Microbiology and Molecular Genetics, University of Pittsburgh School of Medicine 12317 , Pittsburgh, Pennsylvania, USA; University of Michigan Medical School, Ann Arbor, Michigan, USA

**Keywords:** annexin A2, reovirus, viral factories, σNS, µNS, endoplasmic reticulum, actin

## Abstract

Early in infection, mammalian orthoreoviruses (reoviruses) build neo-organelles called viral factories (VFs). These structures incorporate fragments of endoplasmic reticulum (ER) and serve as sites of viral genome replication and particle assembly. Two reovirus nonstructural proteins, σNS and µNS, remodel the ER to produce the vesicles and tubules that form the VF matrix. Using co-immunoprecipitation assays followed by mass spectrometry, we identified annexin A2 (ANXA2), which binds actin and cellular membranes, as a host factor that interacts with reovirus nonstructural proteins. In the absence of ANXA2, VF formation was accelerated in the early phases of reovirus infection, and yields of reovirus were increased. Moreover, organization of the ER network and structure of the actin cytoskeleton were altered in the absence of ANXA2, suggesting that ANXA2 binding to actin is required to maintain ER network morphology. These findings provide evidence that interactions of reovirus nonstructural proteins directly or indirectly with ANXA2 are required for ER remodeling and formation of the membranous fragments that serve as the matrix to assemble reovirus factories.

**IMPORTANCE:**

Reovirus uses ER fragments to build the membranous scaffold of viral factories (VFs). Host proteins that participate in the ER remodeling that precedes factory biogenesis are not known. We identified actin-binding protein ANXA2 as a cellular factor required for maintenance of ER morphology. The absence of ANXA2 destabilizes the actin cytoskeleton and consequently the ER, which accelerates VF biogenesis and enhances reovirus replication. Uncovering the cellular factors used by viruses to form VFs deepens an understanding of viral cell biology and highlights new targets for antiviral drug development.

## INTRODUCTION

Viral factories (VFs) are formed during infection and concentrate viral and host components required for viral replication. These specialized intracellular compartments establish an ideal environment for essential steps in viral infection, including genome replication and packaging and assembly of progeny particles. VFs can also shield viral genomes and proteins from detection by innate immune response sensing mechanisms (1, 2). VFs can be membrane-enclosed (3), devoid of membranes (4), or nonmembrane-enclosed with internal membranes (5, 6). Viruses often transform cellular membrane organelles, such as the endoplasmic reticulum (ER), Golgi, lysosomes, or mitochondria, to build VFs (7–9). The ER is most commonly subverted by viruses to build membrane-enclosed and nonmembrane-enclosed factories (10). A variety of viruses, including mammalian orthoreovirus (reovirus) (5), nidoviruses (11, 12), potyviruses (13), tombusviruses (14), vaccinia virus (15), and Zika virus (16), use the ER to form VFs.

Reoviruses are nonenveloped, segmented, double-stranded RNA (dsRNA) viruses (17) that remodel the ER to build nonmembrane-enclosed VFs (5, 6, 18). Reovirus has a broad host range and infects many mammalian species (17). Reovirus infections in humans are mild or asymptomatic and have been implicated in the development of celiac disease (19). Reovirus factories contain a combination of viral and cellular components (20) and are nucleated by viral nonstructural proteins σNS and µNS (21). In infected cells, the nonstructural proteins induce an unusual remodeling of the ER. σNS binds ER cisternae and transforms these structures into thin tubules, whereas µNS binds the tubules, eliminates their branches, and fragments these structures into small membranous pieces that contribute to the VF scaffold (6, 22). Cellular proteins required for the transformation of the ER, a key step in VF biogenesis, are not known.

In this study, we used co-immunoprecipitation assays followed by mass spectrometry (MS) to identify cellular proteins that interact with reovirus σNS and µNS. We found that both nonstructural proteins precipitated annexin A2 (ANXA2), an actin-binding protein. ANXA2 binds membranes using type II calcium-binding domains (23). In the absence of ANXA2, the actin cytoskeleton and ER network are disrupted, and reovirus factory formation and infection progress more rapidly than in cells expressing ANXA2. These results demonstrate that interactions of ANXA2 with actin stabilize the ER network and suggest that interactions of reovirus nonstructural proteins with ANXA2 function in ER remodeling and VF biogenesis during reovirus infection.

## RESULTS

### ANXA2 associates with reovirus nonstructural proteins

**Fig 1:**
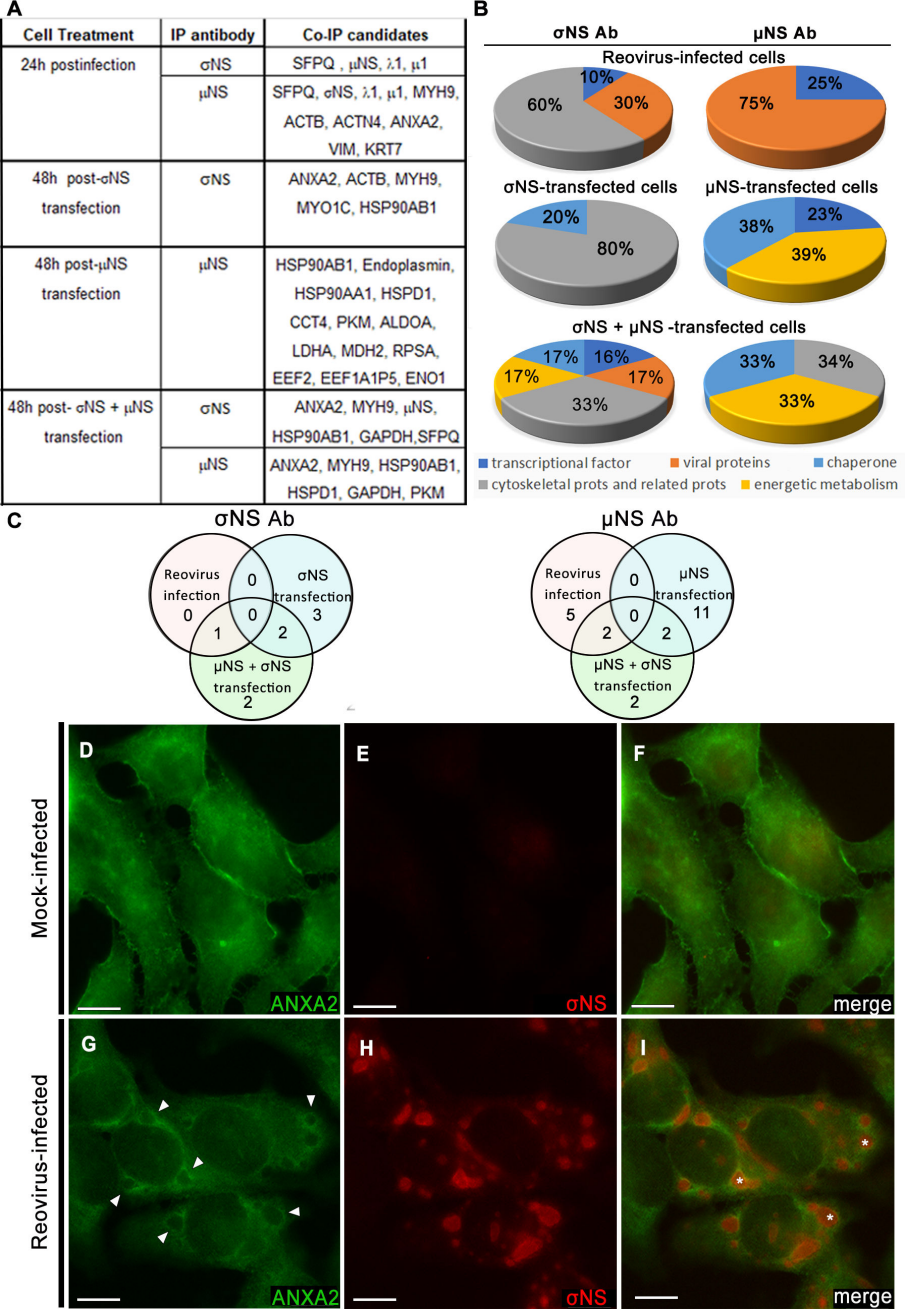
Identification of ANXA2 in a co-immunoprecipitation (co-IP) assay and changes in ANXA2 distribution during reovirus infection. (**A**) HeLa cells were infected with reovirus (24 h post-infection) or transfected (48 h post-transfection) with σNS, µNS, or both proteins and immunoprecipitated with σNS-specific or µNS-specific antibodies. Proteins in the immunoprecipitates were identified using MS. (**B**) Functions of the proteins obtained from the co-IP experiments. (**C**) Venn diagrams of co-IP candidates (excluding viral proteins). (**D–I**) Cells were either mock-infected or infected with reovirus and imaged using confocal immunofluorescence microscopy. (**D–F**) In mock-infected cells, ANXA2 distributes throughout the cytoplasm and concentrates at the cell periphery. (**G–I**) In infected cells, ANXA2 (arrowheads) predominantly surrounds VFs (*). Bars, 10 µm (**D–I**).

To identify host factors that interact with reovirus nonstructural proteins σNS and µNS, we used co-immunoprecipitation assays followed by MS. HeLa cells were either infected with reovirus strain type 1 Lang (T1L) M1 P208S, which forms large, globular VFs (24), or transfected with plasmids encoding σNS, µNS, or both. Cells were lysed and incubated with antibodies specific for σNS or µNS to capture complexes containing reovirus nonstructural and host proteins. Immunoprecipitated proteins were identified by MS at 24 h post-infection or 48 h post-transfection (Fig. 1A).

Proteins identified by MS were distributed in five groups: viral proteins, transcription factors, cytoskeletal proteins, chaperones, and energetic metabolism proteins (Fig. 1B). Only two proteins, ANXA2 and myosin-9 (MYH9), were identified in infected and transfected cells as putative interacting partners of both σNS and µNS (Fig. 1A and C). In reovirus-infected cells, ANXA2 and MYH9 were co-immunoprecipitated by a µNS-specific antibody but not by a σNS-specific antibody. However, ANXA2 and MYH9 were co-immunoprecipitated by the σNS-specific antibody in cells transfected with σNS alone or in combination with µNS. ANXA2 and MYH9 were also co-immunoprecipitated by the anti-µNS antibody, but only in cells expressing both σNS and µNS. ANXA2 is an actin-binding protein involved in endosomal trafficking and cholesterol homeostasis (25). It was also identified as a proviral gene candidate for reovirus replication in siRNA screens (26, 27). Based on the known functions of ANXA2 and its identification in unrelated genetic screens, we evaluated a potential role for ANXA2 in reovirus replication.

ANXA2 distributes to intracellular vesicles and the plasma membrane and is involved in vesicular trafficking, actin remodeling, and regulation of translation (28, 29). To determine the distribution of ANXA2 during reovirus infection, we imaged reovirus-infected cells using confocal immunofluorescence microscopy (Fig. 1D through I). ANXA2 was diffusely distributed in the cytoplasm of mock-infected cells (Fig. 1D). However, in reovirus-infected cells, ANXA2 redistributed and became more intense surrounding the periphery of VFs, where reovirus nonstructural proteins concentrate (Fig. 1G through I).

### ANXA2 expression influences VF formation and nonstructural protein distribution

**Fig 2:**
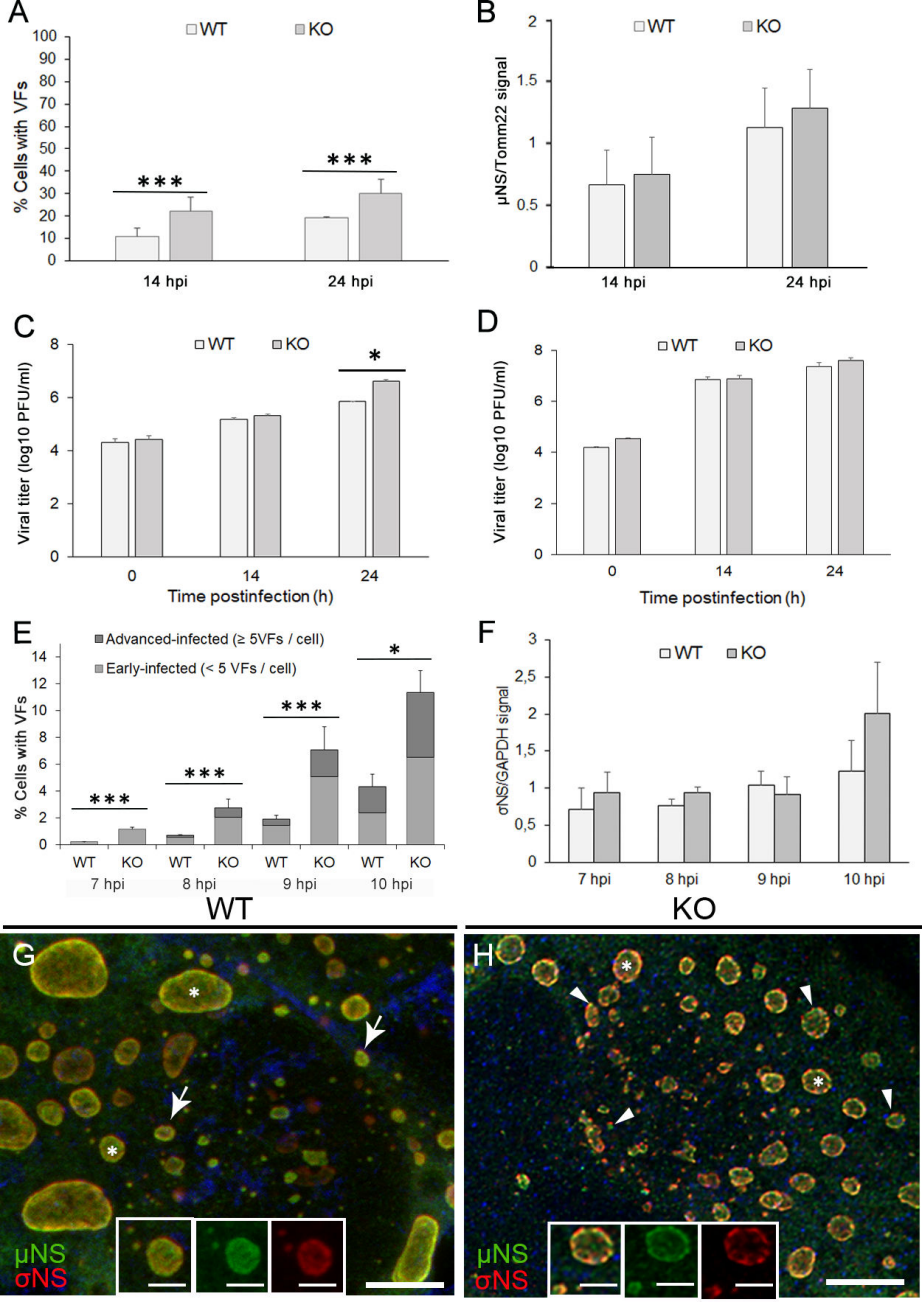
The absence of ANXA2 leads to increased reovirus yields. WT and ANXA2 KO HeLa cells were infected with reovirus and fixed at 14 and 24 h post-adsorption. (**A**) The percentage of infected cells detected by immunostaining with a µNS-specific antibody. (**B**) Reovirus µNS protein levels at 14 and 24 h post-adsorption were determined by immunoblotting using a µNS-specific antibody and normalized to Tomm22. Reovirus titers at 0, 14, and 24 h post-adsorption in (**C**) culture supernatants and (**D**) cell lysates were determined by plaque assay. (**E and F**) WT and ANXA2 KO HeLa cells were infected with reovirus and fixed at 7, 8, 9, and 10 h post-adsorption. (**E**) The percentage of cells with VFs was enumerated following immunostaining with a µNS-specific antibody. Early-infected cells (less than 5 VFs per cell, light gray) and advanced-infected cells (five or more VFs per cell, dark gray) were discriminated in each condition. Statistical analysis was applied to the percentage of all cells with VFs in each condition. (**F**) Reovirus σNS protein levels at 7, 8, 9, and 10 h post-adsorption were determined by immunoblotting using a σNS-specific antibody and normalized to GAPDH. (**G and H**) WT and ANXA2 KO cells were infected with reovirus T1L M1-P208S and imaged at 14 h post-adsorption using confocal microscopy and deconvolution image processing. (**G**) In WT cells, σNS and µNS signals colocalize (arrows) at the periphery of VFs (asterisks). (**H**) In ANXA2 KO cells, σNS and µNS signals uncouple (arrowheads). (**G and H**) Split fluorescent channels of representative VFs are shown in insets. Bars, 5 µm (**G and H**), 2 µm (insets G-H). Results in (**A–F**) are presented as the mean of three independent experiments. Two-tailed Student’s *t*-test: *P < 0.05; ***P < 0.001.

To evaluate a function for ANXA2 in reovirus replication, we infected wild-type (WT) and ANXA2-knockout (KO) HeLa cells with reovirus and assessed the progression of early and late stages of infection. We adsorbed WT and KO cells with reovirus and quantified the number of infected cells at 14 and 24 h post-adsorption using immunofluorescence staining for µNS. Following adsorption with reovirus, there was an approximate twofold increase in the number of KO cells containing VFs relative to VF-containing WT cells at both 14 and 24 h post-adsorption (Fig. 2A), suggesting that ANXA2 limits the formation of reovirus VFs. To determine whether levels of µNS, which is expressed early in infection, are altered in the absence of ANXA2, we quantified µNS levels in reovirus-infected WT and KO cells using immunoblotting. At 14 and 24 h post-adsorption, µNS levels were comparable in WT and KO cells (Fig. 2B), suggesting that ANXA2 is not required for the expression of reovirus proteins.

To quantify viral titer by plaque assay, we adsorbed WT and KO cells with reovirus and collected culture supernatants and cell lysates at 0, 14, and 24 h post-adsorption. Comparison of viral titers in culture supernatants and cell lysates allows us to determine whether there are differences in viral egress. Significantly more infectious virus was detected in supernatants of KO cells than in WT cells at 24 h post-adsorption (Fig. 2C). However, viral titers in lysates of WT and KO cells were comparable at 14 and 24 h post-adsorption (Fig. 2D). These findings suggest that ANXA2 influences steps in reovirus infection that lead to viral release.

To determine whether ANXA2 regulates the formation of VFs early in infection, we quantified cells containing VFs in WT and KO cells at 7, 8, 9, and 10 h post-adsorption in a synchronized infection in which cells were incubated for 30 min on ice during adsorption. In WT cells, VFs were small punctate structures first detectable by immunofluorescence microscopy at approximately 7 h post-adsorption. At all times post-adsorption, the number of VF-containing KO cells was significantly greater than VF-containing WT cells (Fig. 2E; Fig. S1 and S2), and significantly more advanced-infected cells, defined as those with five or more VFs/cell, were observed in KO than WT cells (Fig. 2E). Large VFs (≥1 µm) were visible starting at 10 h post-adsorption in both WT and KO cells (Fig. S3). However, there were no significant differences in σNS protein levels at these early times post-adsorption (Fig. 2F). These data suggest that the absence of ANXA2 accelerates VF formation early in infection, independent of effects on protein expression.

During reovirus infection, σNS and µNS colocalize within VFs and at the factory periphery (21, 30). To determine whether the absence of ANXA2 alters the distribution of σNS and µNS, WT and KO cells were adsorbed with reovirus, and the distribution of σNS and µNS was defined using confocal immunofluorescence microscopy. In WT cells, σNS and µNS colocalize within VFs and concentrate at the VF periphery (Fig. 2G). However, in KO cells, while σNS and µNS distribute to VFs, their colocalization is significantly reduced. (Fig. 2; Fig. S4). Instead, σNS and µNS appear to be in close proximity. These data suggest that ANXA2 influences the distribution of σNS and µNS inside VFs.

### ANXA2 depletion alters the morphology of the ER network

**Fig 3:**
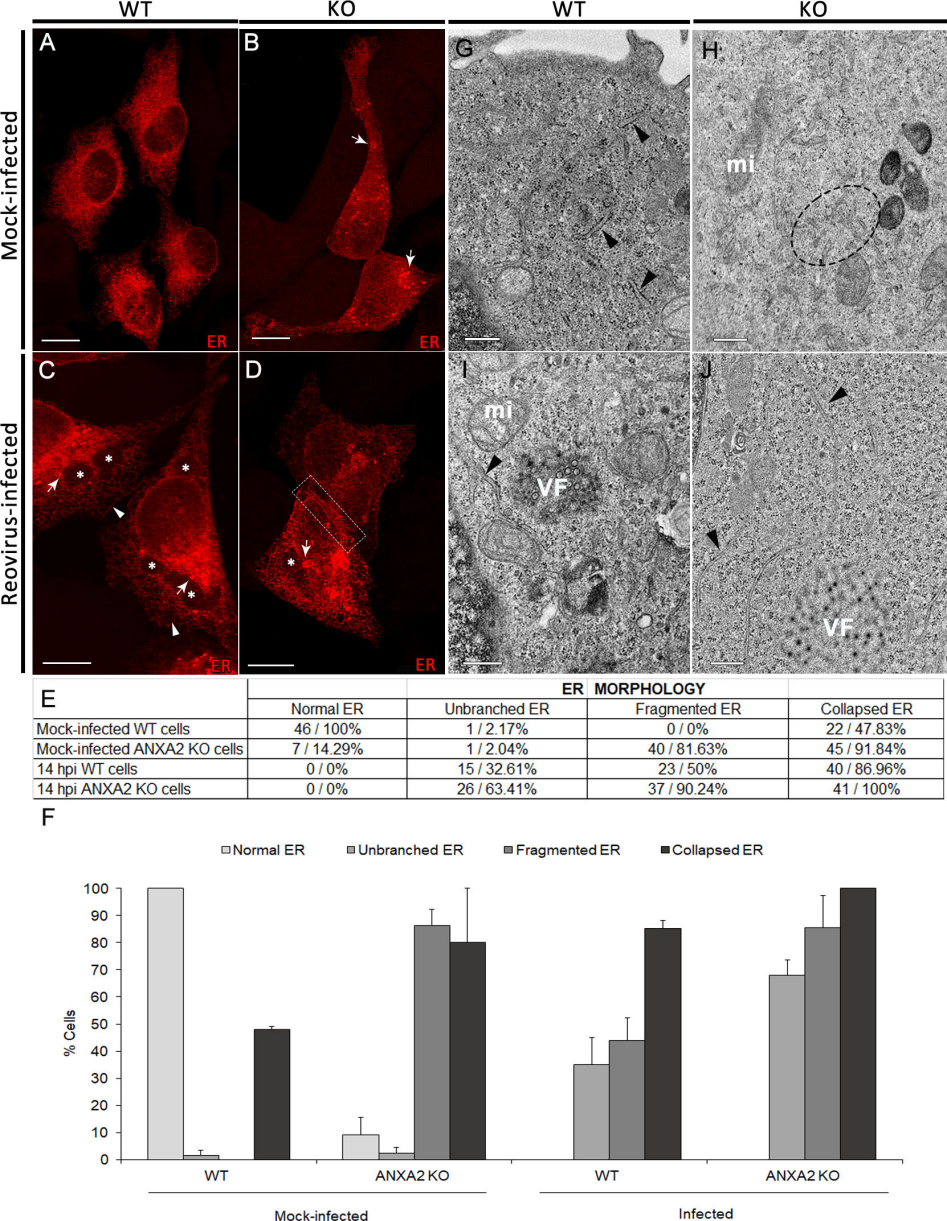
Effects of reovirus infection and absence of ANXA2 on ER morphology. (**A–D**) WT and ANXA2 KO HeLa cells were transfected with mCherry-ER-3 to label the ER and incubated for 24 h. Cells were then mock-infected or infected with reovirus, incubated for 14 or 24 h, and imaged using confocal microscopy. (**A and B**) In the absence of infection, (**A**) WT cells show a characteristic ER network and (**B**) KO cells show dismantling and collapse of the ER (arrows). (**C**) In infected WT cells, the ER surrounds VFs (asterisks), and occasional, collapsed (arrows) and stretched ER cisternae (arrowheads) appear. (**D**) In infected KO cells, collapsed and unbranched linear tubules (dashed rectangle) are more prominent. (**E and F**) Quantification of ER morphology from confocal micrographs. (**E**) The number and percentage of cells with the ER remodeling features observed (normal ER, unbranched ER tubules, fragmented ER, and collapsed ER) in mock-infected or reovirus-infected WT or ANXA2 KO cells. (**F**) Comparison of ER morphologies in these four conditions. Cells at 14 and 24 h post-adsorption were included in the quantification. (**G–I**) Mock-infected or reovirus-infected WT and KO HeLa cells were fixed at 24 h post-adsorption and imaged using transmission electron microscopy. (**G**) Normal ER (arrowheads), (**H**) fragmented ER (dashed ellipse), (**I**) normal ER cisternae surrounding a VF, with some cisternae bifurcated (arrowhead), and (**J**) unbranched linear tubules (arrowheads). VF, viral factory; mi, mitochondria. Bars, 10 µm (**A–D**), 500 nm (**G–J**). Results in (**F**) are presented as the mean of at least three independent experiments.

VFs contain membranous fragments that are derived from extensive remodeling of the ER by σNS and µNS during reovirus infection (6). To determine whether depletion of ANXA2 alters ER morphology, we transfected WT and KO cells with a plasmid expressing mCherry-ER-3 to label the ER, visualized ER morphology using confocal microscopy followed by deconvolution image processing (Fig. 3A through D), and quantified the ER morphologies observed (Fig. 3E and F). In WT cells, the mCherry-ER-3 signal revealed an ER network with a typical reticular pattern (Fig. 3A). However, in KO cells, the ER network was less well defined with an 81.6% increase in cells with fragmented ER and a 44% increase in cells with collapsed ER structures (Fig. 3B, E, and F), demonstrating that the ER network is altered in the absence of ANXA2.

We conducted similar experiments to evaluate ER morphology in WT and KO cells during reovirus infection. Following infection of WT cells, we observed the characteristic ER remodeling induced by reovirus (6), in which the ER becomes thin and fragmented (50% increase) and forms small collapsed structures (39% increase) (Fig. 3C, E, and F), similar to the ER morphology of mock-infected cells in the absence of ANXA2. In reovirus-infected KO cells, the collapsed ER aggregates were more prominent than in mock-infected KO cells (8% increase), and we observed more unbranched linear ER tubules (61% increase) (Fig. 3D, E and F). Representative images of the observed ER morphologies are shown in Fig. S5.

To further characterize these morphological changes, we used transmission electron microscopy (TEM) to image mock-infected and reovirus-infected WT and KO cells. The ER in mock-infected WT cells formed a regular branched tubular network (Fig. 3G). However, in mock-infected KO cells, the ER network was not well organized, and ER tubules were fragmented (Fig. 3H). In reovirus-infected WT cells, ER tubules, including branched tubules, surround the VFs (Fig. 3I). However, in reovirus-infected KO cells, the ER tubules surrounding VFs were long, thin, and unbranched (Fig. 3J). These data demonstrate that the ER network is disrupted in the absence of ANXA2 and during reovirus infection. However, while ANXA2 localizes at the periphery of VFs at 14 and 24 h post-adsorption, it does not colocalize with ER membranes within VFs (Fig. S6).

### ANXA2 depletion disrupts the actin cytoskeleton

**Fig 4:**
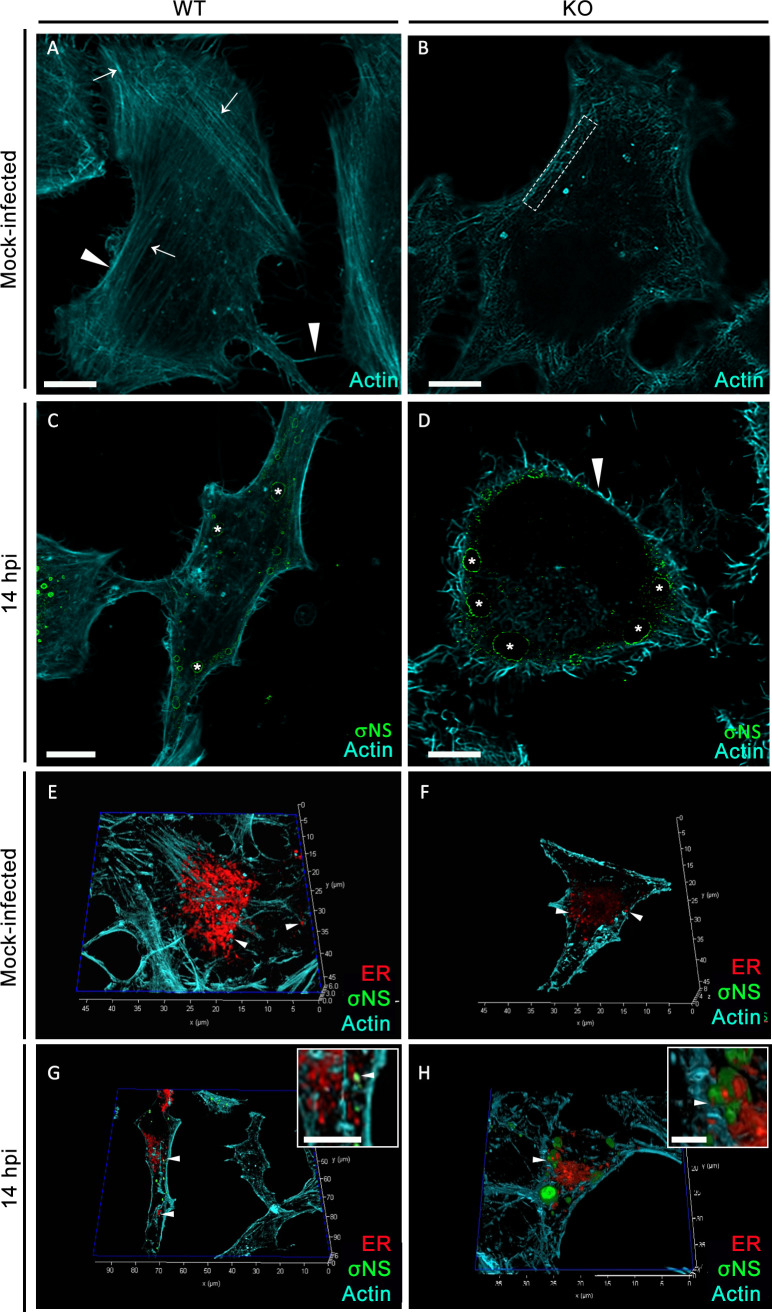
Effects of reovirus infection and absence of ANXA2 on the actin cytoskeleton. (**A–D**) WT and ANXA2 KO HeLa cells were either mock-infected or infected with reovirus and imaged at 14 h post-adsorption using confocal immunofluorescence microscopy followed by deconvolution image processing. (**A and B**) In the absence of infection, (**A**) WT cells show long stress fibers (arrows) and cortical actin (arrowheads), and (**B**) ANXA2 KO cells show broken stress fibers (dashed rectangle) and fewer stress fibers in the cell center. (**C**) In infected WT cells, actin fibers were also less abundant. (**D**) In infected ANXA2 KO cells, stress fibers were dismantled. Only cortical actin (arrowhead) remained. In the images shown in (**C**) and (**D**), VFs (asterisks) are adjacent to the remaining fibers. (**E–H**) 3D reconstructions. (**E and F**) The actin cytoskeleton interacts with the ER network (**E and F**, arrowhead). (**G–H**) VFs interact with actin and the ER (arrowheads). Bars, 10 µm (**A–D**), 2 µm (insets **G–H**).

ANXA2 is an actin-binding protein with multiple functions, including stabilizing the actin cytoskeleton (31, 32). The actin cytoskeleton also interacts with components of the ER to maintain ER morphology (33, 34). To determine whether ANXA2 depletion disrupts the actin cytoskeleton, we used fluorescently labeled phalloidin and confocal microscopy to visualize actin filaments in WT and KO cells. In WT cells, we observed peripheral cortical actin and groups of long actin filaments and stress fibers (Fig. 4A). In KO cells, we observed cortical actin networks only at the cell periphery, and we did not detect long actin filaments (Fig. 4B), suggesting that ANXA2 functions in stress fiber formation or maintenance (35). We also imaged actin filaments in WT and KO cells infected with reovirus at 14 h post-adsorption. Fewer stress fibers were observed in infected WT cells, while cortical actin appeared intact (Fig. 4C). The organization of the actin cytoskeleton in infected KO cells appeared similar to that observed in uninfected KO cells, with only cortical actin networks visible (Fig. 4D). Together, these data demonstrate that the absence of ANXA2 and reovirus infection alters the actin cytoskeleton.

To assess interactions between the actin cytoskeleton and ER network during reovirus infection, we imaged infected WT and KO cells using confocal microscopy and assembled 3D reconstructions using LAS X software. Fluorescent phalloidin was used to detect actin filaments, transfection with mCherry-ER-3 was used to detect the ER network, and an antibody specific for σNS was used to detect VFs. Videos were recorded from the 3D reconstructions of mock-infected (Videos S1, S3, S5 and S7) and reovirus-infected cells (Videos S2, S4, S6 and S8). In frames from videos of mock-infected cells, actin was associated with the ER in both WT and KO cells (Fig. 4E and F). Actin and ER were also in close proximity in VFs of reovirus-infected WT and KO cells (Fig. 4G and H). Together, these data demonstrate that the actin cytoskeleton and ER network are remodeled during reovirus infection and are disrupted in the absence of ANXA2.

### µNS and σNS differentially modify the ER network in WT and KO cells

**Fig 5:**
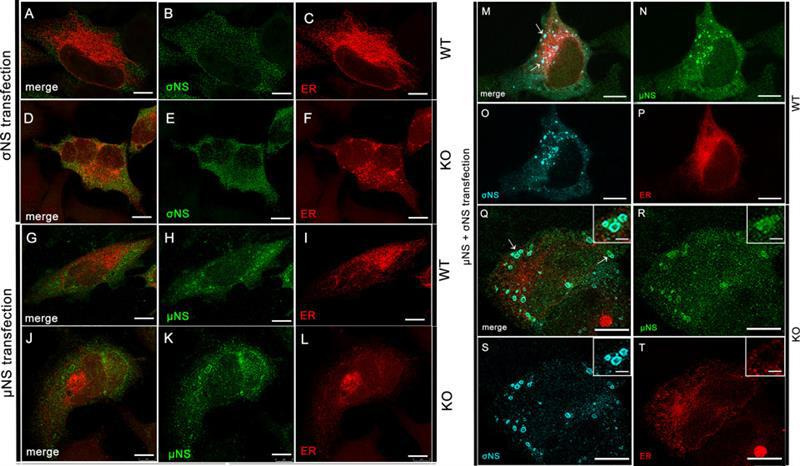
Effects of σNS and µNS on ER morphology. WT and ANXA2 KO HeLa cells were transfected with mCherry-ER-3 along with σNS, µNS, or both proteins and incubated for 24 h. Cells were fixed and imaged using confocal immunofluorescence microscopy and deconvolution image processing. (**A–F**) In WT cells transfected with σNS (**A–C**), ER tubules are stretched, whereas in KO cells transfected with σNS (**D–F**), the ER has a punctate distribution. (**G–L**) In WT cells transfected with µNS (**G–I**), the ER is unbranched and fragmented, whereas in ANXA2 KO cells transfected with µNS (**J–L**), the ER network is collapsed. (**M–T**) Transfection of both σNS and µNS causes ER remodeling in both WT and KO cells, comparable to that observed in infected WT cells. (**M**) In WT cells, σNS and µNS colocalize (arrows). (**Q**) In ANXA2 KO cells, σNS and µNS uncouple (arrows). Regions corresponding to high-magnification insets are indicated by white arrows. Bars, 10 µm (**M–T**), 2 µm (insets **Q–T**).

To determine how ANXA2 depletion affects ER remodeling by σNS and µNS, we transfected WT and KO cells with mCherry-KDEL together with σNS, µNS, or both proteins and monitored ER morphology using confocal microscopy. In WT cells expressing σNS, ER tubules were stretched at the cell periphery (Fig. 5A through C), as observed previously (6). However, in the absence of ANXA2, the ER remained as clusters of small collapsed cisternae throughout the cell (Fig. 5D through F). In both the presence and absence of ANXA2, σNS was distributed diffusely in the cytoplasm. In WT cells expressing µNS, ER tubules were unbranched and fragmented, with some long unbranched tubules remaining (Fig. 5G through I), as observed previously (6, 36). In the absence of ANXA2, the ER was mostly collapsed at the periphery of the nucleus (Fig. 5J through L). In both WT and KO cells, µNS was distributed in the cytoplasm and formed factory-like structures. In WT cells expressing both σNS and µNS, ER remodeling was similar to that observed during reovirus infection, and σNS and µNS co-localized in factory-like structures (Fig. 5M through P). In ANXA2 KO cells transfected with both nonstructural proteins, the ER was highly unstructured, and some small foci of collapsed ER were observed adjacent to factory-like structures (Fig. 5Q through T). The most common ER morphologies observed were quantified (Fig. S7). Viral nonstructural proteins also were concentrated in these factory-like structures, but they did not colocalize, similar to that observed in reovirus-infected KO cells (Fig. 2H). Collectively, these data suggest that the absence of ANXA2 modifies the effects of reovirus nonstructural proteins on the morphology of the ER network, increasing the portions of collapsed ER and uncoupling σNS and µNS interactions.

### The distance between reovirus nonstructural proteins, ER, and actin is altered in the absence of ANXA2

**Fig 6:**
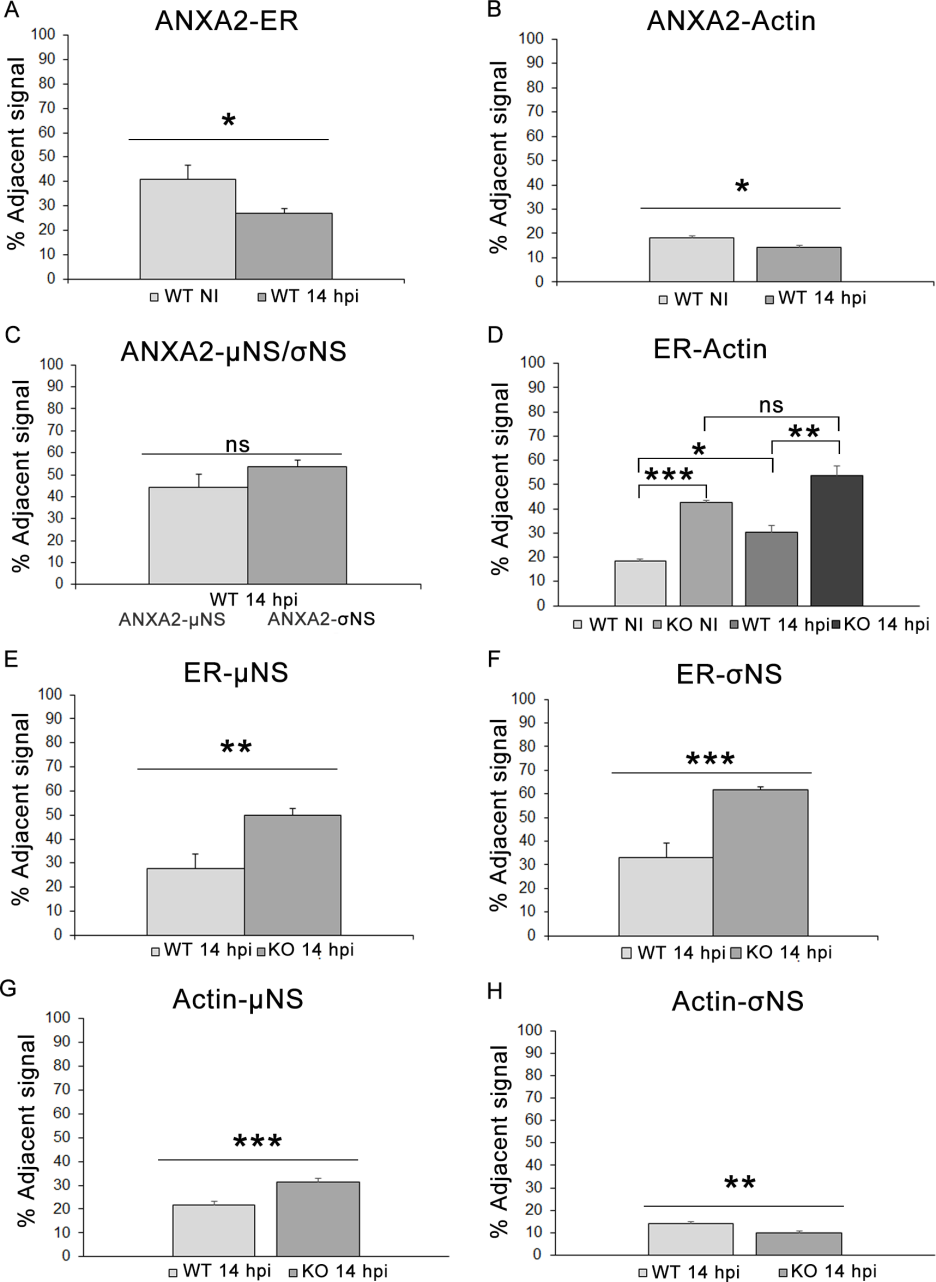
Quantification of the minimum distance between fluorescence signals associated with reovirus nonstructural proteins and cell components. The minimum distances between ANXA2, σNS, and µNS and the ER and actin were determined and compared in pairs, and the percentage of adjacent signal between the two signals was calculated. Distances between (**A**) ANXA2 and ER, (**B**) ANXA2 and actin, or (**C**) ANXA2 and the nonstructural proteins were determined and compared. Distances between (**D**) the ER and actin and (**E, F**) the ER and nonstructural proteins. (**G, H**) Distances between actin and nonstructural proteins. Results are the mean of at least seven images processed for each condition. Unpaired two-tailed Student’s *t*-test. *P < 0.05; **P < 0.01; ***,*P* < 0.001; ns, non-significant.

To better understand the dynamics of reovirus nonstructural proteins and ANXA2 in relation to the ER and actin cytoskeleton, we quantified minimum distances between the fluorescence signals of σNS, µNS, ANXA2, the ER, and the actin cytoskeleton in mock-infected and reovirus-infected WT and KO cells using the z-stack analyzer plug-in for FIJI software. This quantification method is based on the minimum distances separating the signals of two different fluorescent channels. More than 1 million measurements of minimum distances were obtained and ordered from shortest (0 nm) to longest (several µm). Adjacent signal distances were obtained by subtracting measurements between 0 and 150 nm from all measurements obtained. Thus, the adjacent signal measurements correspond to all measurements between the first channel and second channel with a maximum separation of three pixels (with a pixel size of 52 nm) (Fig. S8). The percentage of adjacent signal measurements was calculated as the ratio of all measurements within the adjacent signal interval to the total minimum measurements multiplied by 100 (Fig. 6).

We first determined the distance between ANXA2 and the ER and ANXA2 and the actin cytoskeleton in mock-infected and reovirus-infected WT cells. The distance between ANXA2 and the ER (Fig. 6A) and ANXA2 and actin (Fig. 6B) increased in infected cells relative to mock-infected cells, suggesting that the association of ANXA2 with both the ER and actin is altered during reovirus infection. We also analyzed the distance between ANXA2 and the nonstructural proteins in infected WT cells. The distance between ANXA2 and σNS and ANXA2 and µNS was comparable (Fig. 6C), suggesting that the nonstructural proteins form a complex during infection that is in close proximity to ANXA2.

We next determined the distance between the ER and actin cytoskeleton in mock-infected and reovirus-infected WT and KO cells. The distance between the ER and actin decreased in both mock-infected and reovirus-infected KO cells relative to mock-infected WT cells (Fig. 6D). Additionally, the distance between the ER and actin decreased in infected WT cells relative to mock-infected WT cells. Together, these data suggest that the disruption of the ER network and actin cytoskeleton that occurs during reovirus infection or in the absence of ANXA2 increases an association of actin with the ER.

We also analyzed the distance between the reovirus nonstructural proteins and the ER and actin in reovirus-infected WT and KO cells. The proximity of the ER to either µNS (Fig. 6E) or σNS (Fig. 6F) was significantly greater in KO cells than in WT cells, suggesting that in the absence of ANXA2, the nonstructural proteins are more closely associated with the ER than they are in WT cells. However, the distance between µNS and actin was decreased in KO cells (Fig. 6G), while the distance between σNS and actin was increased (Fig. 6H). These data suggest that ANXA2 influences the distribution of σNS, µNS, and actin in VFs and are consistent with our morphological observations.

### Complementation of ANXA2 KO cells with ANXA2 recovers ER network morphology

**Fig 7:**
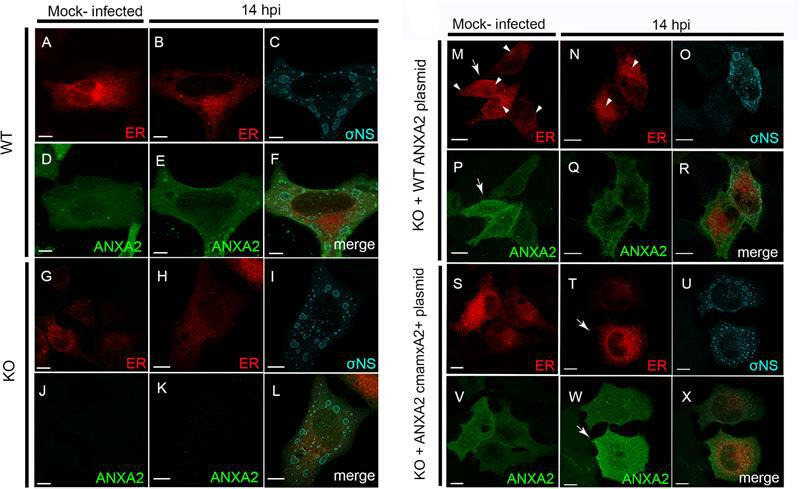
Effects of WT ANXA2 and mutant ANXA2 cmamxA2+ in ANXA2 KO HeLa cells. WT and ANXA2 KO HeLa cells were either mock-transfected or transfected with ANXA2 or ANXA2 cmamxA2+, incubated for 24 h, and either mock-infected or infected with reovirus. At 14 h post-adsorption, cells were fixed and imaged using confocal immunofluorescence microscopy and deconvolution image processing. (**A–F**) In WT cells, reovirus infection produces thin, linear ER tubules and ER fragmentation and collapse. (**G–L**) In ANXA2 KO cells, reovirus infection dismantles the ER network, forming small, collapsed ER clusters. (**M–R**) In both mock-infected and infected ANXA2 KO cells complemented with WT ANXA2, the ER shows a pattern similar to that in WT cells, although some collapsed ER structures were not restored (arrowheads in **M and N**). (**S–X**) In ANXA2 KO cells complemented with ANXA2 cmamxA2+, the effects were similar to those in cells complemented with WT ANXA2. Higher levels of WT ANXA2 correlate with the efficiency of ER restoration (arrows in **M, P, T, and W**). Bars, 10 µm.

To ensure that potential off-target effects of ANXA2 gene disruption do not contribute to the alteration of the ER network observed in KO cells relative to WT cells, we transfected KO cells with a plasmid encoding WT ANXA2 and compared ER morphology in mock-infected and reovirus-infected cells (Fig. 7). Mock-infected and reovirus-infected WT and ANXA2 KO cells were used as controls for ER morphology (Fig. 7A through L). In KO cells transfected with ANXA2, the ER morphology in mock-infected cells (Fig. 7M) was similar to that in mock-infected WT cells (Fig. 7A), with restoration of the tubular ER network. However, some collapsed ER cisternae were observed in the complemented cells (Fig. 7M). The relative ER network recovery was proportional to the level of ANXA2 in the transfected cells (Fig. 7P and Q). ER remodeling in reovirus-infected KO cells complemented with ANXA2 (Fig. 7N) was comparable to ER remodeling in reovirus-infected WT cells (Fig. 7B), with an increase in thinned and fragmented ER tubules and areas of collapsed ER (Fig. 7B and N). These data demonstrate that transient expression of ANXA2 in KO cells restores ER morphology.

To test whether ANXA2 maintains ER structure by binding ER membranes, we transfected KO cells with a plasmid encoding ANXA2 cmamxA2+, which lacks the calcium-binding domains and cannot interact with membranes (37), and compared ER morphology in mock-infected and reovirus-infected cells. In mock-infected KO cells expressing ANXA2 cmamxA2+, we observed a tubular ER network, similar to the ER morphology in WT cells and KO cells expressing ANXA2 (Fig. 7S). In reovirus-infected KO cells complemented with cmamxA2+ (Fig. 7T), ER remodeling was similar to ER remodeling in reovirus-infected WT cells and KO cells expressing ANXA2, with an increase in fragmented ER tubules and areas of collapsed ER. As observed previously, levels of ANXA2 correlate with the extent of ER network restoration (Fig. 7V and W). Collectively, these data suggest that the capacity of ANXA2 to bind ER membranes is not required to maintain ER structure.

To confirm a function for ANXA2 in reovirus infection, we used CRISPR/Cas9 gene editing to engineer a new clonal HeLa cell line with a nonfunctional ANXA2 gene. As a control, the rederived ANXA2 KO cells were complemented with wild-type ANXA2 (KO+) (Fig. S9). The rederived WT, KO, and KO+ cells were adsorbed with reovirus, and virus replication was quantified. The percentage of cells with VFs was significantly increased in KO cells at 14 and 24 h post-infection relative to WT and KO+ cells, and significantly higher titers of infectious virus were detected in culture supernatants of KO cells relative to WT or KO+ cells at 14 h post-adsorption (Fig. S10). Thus, these data suggest that ANXA2 expression maintains ER morphology and influences the rate of VF formation.

## DISCUSSION

**Fig 8:**
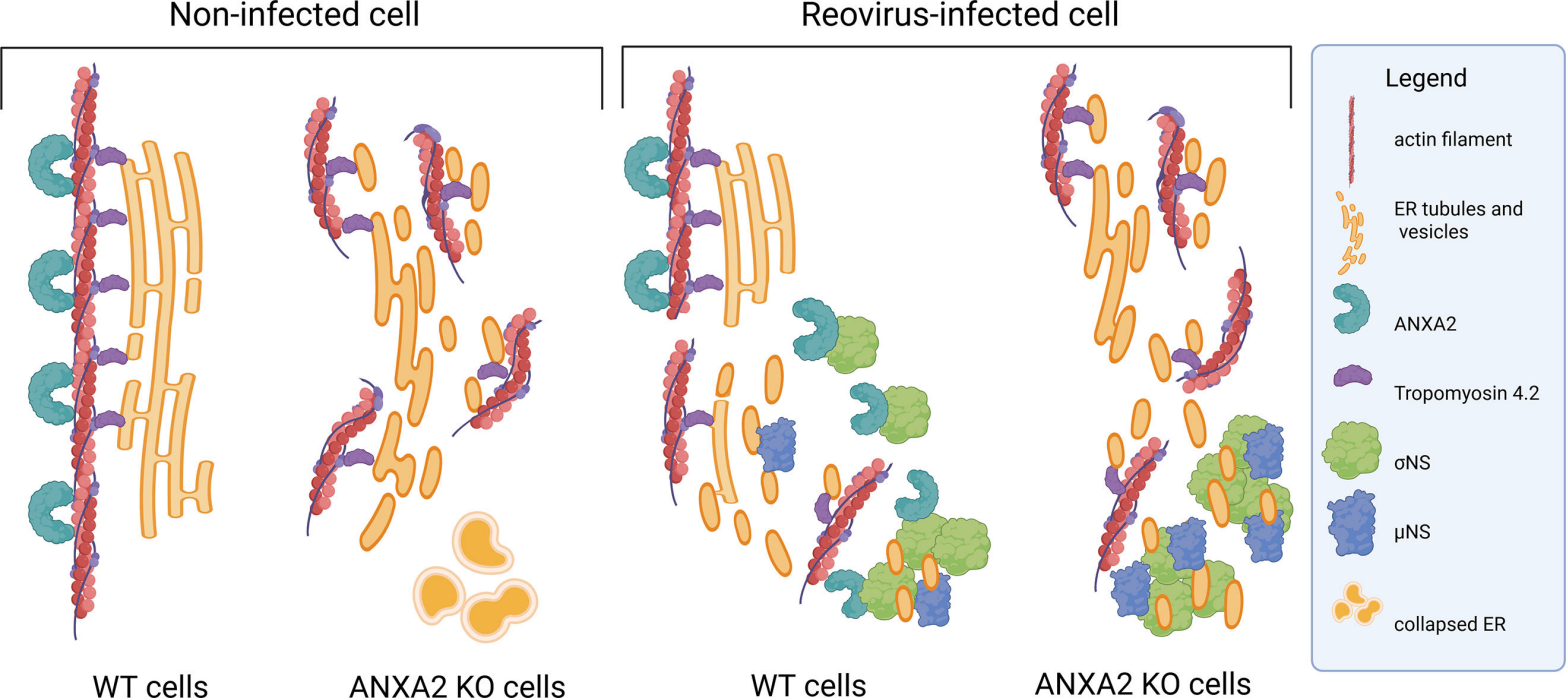
Model of ANXA2 and reovirus infection. ANXA2 is required for ER morphology and interactions with the actin cytoskeleton. In the absence of ANXA2, the ER and actin cytoskeleton are dismantled. Following reovirus infection of cells that lack ANXA2, ER fragments and actin are recruited more efficiently to VFs, which accelerates reovirus replication and release. By virtue of its functions in regulating ER homeostasis and interactions with actin, ANXA2 regulates reovirus infection.

During the early stages of reovirus infection, nonstructural proteins σNS and µNS function cooperatively to build functional VFs. These proteins remodel the ER to form small membranous fragments that contribute to the VF matrix. How σNS and µNS fragment and cleave the ER is not known. In this study, we identified ANXA2 as a host factor that interacts with nonstructural proteins σNS and µNS. We discovered that ANXA2, which binds actin, calcium, and lipids and functions in endocytosis, exocytosis, and the formation of lipid microdomains, also maintains the morphology of the ER network and influences VF formation. In the absence of ANXA2, the ER network is fragmented, similar to that observed during reovirus infection. In reovirus-infected cells lacking ANXA2, VF formation is accelerated relative to WT cells. Based on these data, we propose a model in which σNS and µNS interact directly or indirectly with ANXA2 to interrupt engagement of ANXA2 with actin fibers associated with the ER (Fig. 8). Disruption of ANXA2-actin interactions facilitates dismantling of the actin cytoskeleton and ER network, allowing the formation of ER membranous fragments that coalesce to form the reovirus factory matrix. In the absence of ANXA2, the actin cytoskeleton and ER are dismantled prior to infection, allowing for rapid recruitment of ER fragments to VFs and accelerated reovirus factory formation.

Early in reovirus infection, when VFs are forming, all viral and host factors required for VF assembly must be available. We hypothesize that alterations in ANXA2 distribution during infection destabilize the ER and facilitate the acquisition of ER fragments that nucleate VFs. In the absence of ANXA2, ER fragments are available at the initiation of infection and, therefore, VFs can form more rapidly in the presence of unchanged levels of nonstructural proteins. Mutagenesis experiments with the reovirus nonstructural proteins are required to test this hypothesis.

Several viruses use ANXA2 at different steps of replication, including entry, assembly, and egress. ANXA2 is a putative receptor for respiratory syncytial virus (38), mediates membrane fusion and internalization of porcine epidemic diarrhea virus (39), regulates hepatitis C virus RNA synthesis and assembly (40, 41), and contributes to assembly of human immunodeficiency virus (42). ANXA2 also functions in replication of members of the *Reovirales* order, including avian reovirus (43) and bluetongue virus (BTV) (44). ANXA2 is required for caveolin-dependent endocytosis of avian reovirus (45, 46) and functions in BTV egress by interacting with viral nonstructural protein NS3 (47). In each of these cases, ANXA2 interactions with membranes are required for its activities in viral replication. We found that ANXA2 stabilizes the ER network and slows VF biogenesis using a mechanism that is not dependent on membrane binding. Complementation of ANXA2 KO cells with an ANXA2 mutant lacking the membrane-binding domain (cmamxA2+) restored ER morphology in a manner comparable to WT cells and ANXA2 KO cells complemented with WT ANXA2 (Fig. 7). These data suggest that the function of ANXA2 in maintaining ER morphology is independent of binding ER membranes. We identified a new function for ANXA2 in viral infection that is dependent on the interaction of ANXA2 with the actin cytoskeleton.

The actin cytoskeleton scaffolds the ER network using TPM4 (34), which binds actin and forms copolymers with short actin filaments that interact with the ER. In the absence of TPM4, the ER network is destabilized (34, 48), similar to that observed in the absence of ANXA2 (Fig. 3). However, a function for ANXA2 in maintaining ER morphology has not been previously reported. Our data demonstrate that ANXA2 is required to maintain ER network morphology (Fig. 3). In the absence of ANXA2, fragmented and collapsed ER increased by 81.6% and 44%, respectively (Fig. 3I and J). A function for ANXA2 in maintaining ER morphology is further supported by our finding that the distance between ANXA2 and the ER and ANXA2 and actin increases in reovirus-infected WT cells in which the ER is fragmented (Fig. 6A and B). The increased association of actin with the ER in reovirus-infected WT and KO cells relative to mock-infected WT and KO cells may be due to the distribution of actin and ER fragments within VFs (Fig. 6D). These data suggest that ANXA2 stabilizes the ER by interactions with the actin cytoskeleton. We also observed that destabilization of the ER network by ANXA2 gene editing (Fig. 3; Fig. S1) results in accelerated VF biogenesis, further supporting our model in which preemptive dismantling of the ER allows for rapid recruitment of ER fragments to VFs and accelerated reovirus factory formation.

Dynamic changes in the actin cytoskeleton are required for many cellular functions, including endocytosis, intracellular transport of macromolecular cargo, and exocytosis. Viruses interact with elements of the cytoskeleton at several steps of infection, including cell entry, protein synthesis, genome replication, and egress. Cytoskeletal elements, including microtubules (49) and actin filaments (Fernandez de Castro et al., unpublished results), are required for reovirus replication. Reovirus binding to PirB, which serves as a receptor for reovirus in some types of cells (50, 51), activates receptor signaling and triggers endocytosis (51). Binding of some ligands to PirB induces depolymerization of actin filaments (52), which may be required for reovirus entry. We used fluorescently labeled phalloidin to demonstrate that reovirus infection alters the actin cytoskeleton. Reovirus-infected cells have fewer stress fibers than mock-infected cells, while cortical actin appears intact (Fig. 4C). Thus, actin serves a key function in reovirus replication.

Nonmembrane-enclosed VFs have properties of biomolecular condensates, including liquid-like properties (53–55), and can be either devoid of membranes or contain membranes within the factory. Reovirus factories are nonmembrane-enclosed but contain fragments of ER membranes that contribute to the VF matrix and may serve as a scaffold for RNA synthesis and viral particle assembly (6). Other members of the *Reovirales*, such as rotaviruses, also assemble nonmembrane-enclosed VFs. However, membranes have not been observed within these factories, suggesting that the composition of the factories formed by reovirus and rotavirus differs.

Biomolecular condensates were once thought to be devoid of membranes (56). However, membrane-associated condensates form at the plasma membrane, ER, nuclear envelope, peroxisomes, and autophagosomes (57). The thermodynamics of condensate formation depend on the local concentration of phase-separating macromolecules, such as proteins or RNA. A lower threshold concentration is required for membrane-associated condensate formation (58). Our data suggest that reovirus factories are membrane-associated condensates. Reovirus nonstructural proteins induce fragmentation and vesiculation of ER membranes (Fig. 3C) (6), and 3D electron tomography shows ER membranes surrounding VFs (6) as well as membrane fragments within VFs (6, 18). When ER membrane fragments are present prior to reovirus infection, as occurs in the absence of ANXA2, VFs are visible by immunofluorescence staining of nonstructural proteins as early as 7 h post-infection (Fig. 2), which is approximately 2 h earlier than observed in cells expressing ANXA2. These data suggest that ER fragments serve as nucleation sites for functional VF biogenesis.

Within the order *Reovirales*, several cellular factors required for VF formation or function have been described. For example, casein kinase 2 and phosphatase 2A are required for BTV (59), ADP ribosylation factor 1 (ARF1) is required for grass carp reovirus (60), and casein kinase 1α (61), casein kinase 2 (62), small ubiquitin-like modifier (63), and perilipin1 (64) are required for rotavirus. In the case of reovirus, the TRiC chaperonin (65), hsc70 (66), and ER fragments and vesicles (6) localize to factories and are required for VF function but not formation. We found that ANXA2 localizes to the periphery of VFs (Fig. 1) and may regulate VF biogenesis by direct or indirect interactions with nonstructural proteins σNS and µNS.

In this study, we discovered that ANXA2 stabilizes the ER. Disrupting the binding of ANXA2 to actin perturbs ER membranes, leading to ER dismantling. Reovirus infection of cells lacking ANXA2 leads to accelerated VF formation and enhances the kinetics of reovirus replication. We found that reovirus dismantles actin fibers, which is essential for the formation of reovirus factories. This work establishes the foundation for future research on how ER membranes, the cytoskeleton, and the cellular factors identified in our proteomic screens coordinate with reovirus proteins to assemble functional VFs. As these mechanisms may be conserved across virus families, this work could illuminate new targets for broadly applicable antiviral therapeutics.

## MATERIALS AND METHODS

### Cells

HeLa cells (WT) and ANXA2 knockout (KO) HeLa cells were obtained from Dr. Martin Kast at the University of Southern California (36). Both cell lines were propagated in Dulbecco’s modified Eagle’s medium (DMEM; D6429; Sigma) supplemented to contain 10% fetal bovine serum (FBS), 100 U/mL penicillin G, 100 µg/mL streptomycin (Gibco), 0.25 µg/mL amphotericin B, nonessential amino acids, 2 mM L-glutamine, and 1 mM sodium pyruvate (Sigma). L929 fibroblast cells were propagated in DMEM (D6429; Sigma) supplemented to contain 10% FBS, 100 U/mL penicillin G, 100 µg/mL streptomycin (Gibco), nonessential amino acids (Sigma), and 2 mM L-glutamine.

New clonal HeLa ANXA2 knockout cells (ANXA2 KO cells) and ANXA2 complemented cells (ANXA2 KO+) (Fig. S9 and S10) were engineered by transfecting HeLa S3 cells with either empty CRISPR-KO transfer vector (lentiCRISPRv2-blast) or lentiCRISPRv2-blast encoding ANXA2-specific guide sequence (5′-GGTCCTTCTCTGGTAGGCGA-3′ from the human Brunello CRISPR knockout pooled library) using LipfectAMINE 3000 according to the manufacturer’s instructions (67). At 48 h post-transfection, cells were selected with medium containing 10 µg/mL blasticidin for 5 days. Single-cell clones were selected from surviving cells and screened for ANXA2 expression. To confirm the specificity of *Anxa2* knockout, the rederived ANXA2 KO cells were transduced with lentivirus vector pCSIB-expressing ANXA2 (engineered by Gibson assembly). Two days post-transduction, cells were selected with medium containing 10 µg/mL blasticidin for 5 days, and ANXA2 expression in the surviving cell population was assessed (ANXA2 KO+).

### Viruses

Reovirus strain T1L M1-P208S is identical to reovirus strain T1L, with the exception of a proline-to-serine substitution at position 208 of the µ2 protein (M1 gene). This substitution results in a change in VF morphology from filamentous to globular (24). Site-directed mutagenesis was used to engineer the P208S substitution in the M1 gene with the following primers: forward, 5′ CATTTCGGGGTAGCAATTGATGAAAATGTGCCAACATTAAATCTAG 3′; reverse, 5′ CTAGATTTAATGTTGGCACATTTTCATCAATTGCTACCCCGAAATG 3′. Both strains were recovered by plasmid-based reverse genetics (68), purified using cesium gradient centrifugation (69), and propagated at a multiplicity of infection (MOI) of 5 PFU/cell at 33°C for 65 h to yield working stocks. Viral titers were determined by plaque assay using L929 cells (70).

### Viral titration by plaque assay

Titers of infectious reovirus were determined by plaque assay using L929 cells (70). Cells were adsorbed with tenfold serial dilutions of the infected sample or viral stock at 37°C for 1 h. Following viral adsorption, cells were overlaid with DMEM supplemented to contain 0.5% agarose, 1% penicillin/streptomycin, 1% nonessential amino acids, 0.1% gentamicin, and 2% FBS. Cells were incubated for 7 d, fixed with 10% formaldehyde for 1 h, and stained with 0.1% crystal violet for 5 min to visualize viral plaques. Viral titer is expressed as plaque-forming units per mL of culture supernatant or cell lysate (PFU/mL).

### Co-immunoprecipitation

HeLa cells cultivated in two P150 plates per condition were infected with reovirus T1L M1 P208S at an MOI of 20 PFU/cell or transfected with T3D σNS, µNS, or both expression plasmids (71) using the calcium phosphate method (72). At 24 h post-infection (hpi) or 48 h post-transfection, cells were collected and lysed using co-immunoprecipitation (co-IP) buffer (20  mM Tris [pH 8], 137  mM NaCl, 2  mM EDTA, and 1% NP-40 substitute) supplemented with protease inhibitors (Roche, 11873617001) at 4°C for 30  min with rotation. Lysates were cleared by centrifugation, and supernatants were incubated with σNS-specific or µNS-specific antibodies at 4°C overnight and incubated with A Dynabeads (Thermo Fisher, 10001D) at room temperature (RT) for 1.5 h with rotation. Beads were collected from the supernatant using a magnet and washed six times with cold lysis buffer. Bound proteins were eluted by boiling in Laemmli sample buffer (Bio-Rad) with 10% β-mercaptoethanol for 15  min. Proteins were identified by MS (matrix-assisted laser desorption/ionization with time-of-flight [MALDI-ToF) and analyzed using MASCOT software (Matrix Science) to obtain qualitative identification of the resultant peptides.

### Immunoblotting

Cells harvested for protein extraction were lysed in 2× Laemmli sample buffer (Bio-Rad) containing 10% β-mercaptoethanol and incubated at 95°C for 15 min. A volume of 10 µL of each sample was electrophoresed in 4-20% Mini-Protean TGX gels (Bio-Rad). Following electrophoresis, proteins were transferred from gels to PVDF membranes using a Transblot turbo transfer pack (Bio-Rad). Membranes were blocked at RT for 1 h with 3% nonfat milk in phosphate-buffered saline (PBS) containing 0.05% Tween 20 and incubated overnight at 4°C with 1:1,000 rabbit σNS-specific polyclonal antibody VU82 (73), 1:12,000 chicken µNS-specific antiserum provided by John Parker (Cornell University) (20), and 1:2,000 mouse ANXA2-specific monoclonal antibody (Proteintech) in blocking buffer. Mouse tubulin-specific, rabbit Tomm22-specific, or mouse GAPDH-specific antibodies in blocking buffer were used for controls. After washing with 0.1% Tween 20 in PBS, membranes were incubated with secondary antibodies conjugated with horseradish peroxidase (HRP, POD) at RT for 1 h. Target proteins were detected using ECL solutions (SuperSignal West Dura Extended Duration Substrate, Thermo Scientific) and a ChemiDoc Imager (BioRad). Band intensity was calculated using Quantity One software (BioRad) and normalized to the respective control proteins. Three independent replicates were analyzed for each experiment.

### Confocal microscopy, immunofluorescence, quantification of ER morphology, and nonstructural proteins colocalization

HeLa cells cultivated on glass coverslips in 6-well plates were adsorbed at 37°C with reovirus at an MOI of 1 PFU/cell and incubated at 37°C for 1 h. For experiments probing events at early times post-adsorption (7, 8, 9, and 10 h post-adsorption), cells were adsorbed on ice for 30 min to synchronize the onset of infection. Following incubation, cells were fixed with 4% PFA in PBS (pH 7.4) at RT for 20 min, permeabilized with 0.25% saponin, and blocked in saturation buffer (0.25% saponin, 2% FBS in PBS) for 30 min. Antibodies and probes were diluted in saturation buffer as follows: 1:1,000 for rabbit σNS-specific antibody VU82, 1:200 for guinea pig σNS-specific antiserum (21), chicken µNS-specific antiserum, rabbit giantin-specific polyclonal antiserum (BioLegend), 4′,6-diamidino-2-phenylindole (DAPI), and phalloidin (Thermo Fisher Scientific), 1:50 for mouse ANXA2-specific monoclonal antibody (Abcam), and 1:500 for Alexa Fluor-conjugated secondary antibodies (Invitrogen). Images were obtained using a Leica Stellaris 5 confocal microscope, followed by a deconvolution processing using the integrated module Lightning in LAS X software (Leica Microsystems).

Confocal images obtained for 3D reconstruction were processed and built using the 3D integrated module in LAS X software. Quantification of ER morphology changes was conducted using confocal images of mock-infected WT cells (*n* = 46), reovirus-infected WT cells (*n* = 46), mock-infected ANXA2 KO cells (*n* = 49), and reovirus-infected ANXA2 KO cells (*n* = 41). ER structures were classified in four categories: (i) regular ER, (ii) fragmented ER, (iii) unbranched ER, and (iv) collapsed ER. Images were obtained using a Leica Stellaris 5 confocal microscope at a magnification of x63 or x100 using LAS X software.

Confocal images used for colocalization studies were processed using the JACoP plugin (74) in FIJI software (75). We studied σNS and µNS colocalization in 36 VFs per condition. Pearson’s and Mander’s coefficients were obtained for each VF. Mander’s coefficients were calculated as Mander’s 1 (fraction of red signal [σNS] overlapping green signal [µNS]) and Mander’s 2 (fraction of green signal [µNS] overlapping red signal [σNS]).

### Quantification of reovirus infection

Cells were adsorbed with reovirus at an MOI of 1 PFU/cell at 37°C for 1 h. The inoculum was removed, and cells were incubated at 37°C for various intervals. Cells were fixed with 4% PFA in PBS (pH 7.4) at RT for 20 min, permeabilized with 0.25% saponin, and blocked with 0.25% saponin and 2% FBS in PBS. Fixed and permeabilized cells were incubated sequentially with chicken µNS-specific antiserum and Alexa Fluor 488-conjugated chicken-specific antibody in PBS containing 0.25% saponin and 2% FBS. Stained cells were imaged using a Leica DMI6000B fluorescence microscope equipped with a 40× air objective. Ten fields of view per condition were imaged by an observer blinded to the conditions of the experiment. In each field of view, 25–75 cells were quantified. Cells with at least one VF were considered infected. Cells with VFs were quantified using LAS X software.

### Transmission electron microscopy

Cells were propagated in six-well plates, infected with reovirus, and processed for embedding in resin as described (1, 6). Cells were incubated for 24 h and fixed with a mixture of 4% PFA and 1% glutaraldehyde in 0.4 M HEPES buffer, pH 7.4, at RT for 1 h. Cells were postfixed at 4°C for 1 h with a mixture of 1% osmium tetroxide and 0.8% potassium ferricyanide in water and dehydrated in 5-minute steps with increasing concentrations of acetone (50%, 70%, 90%, and twice in 100%) at 4°C. Cells were processed for embedding in the epoxy resin EML-812 (TAAB Laboratories) by incubation overnight with a 1:1 mixture of acetone and resin at RT. After embedding in 100% resin for 8 h, samples were polymerized at 60°C for 48 h. Serial sections of 50–70 nm thickness were obtained using a UC6 ultramicrotome (Leica Microsystems) and collected on uncoated 300-mesh copper grids (TAAB Laboratories). Sections were stained with 4% uranyl acetate and Reynold’s lead citrate prior to TEM imaging. Images were acquired using a 100 kV JEOL JEM 1011 TEM, equipped with a Gatan ES1000WW digital camera.

### Reovirus nonstructural protein transfections and ANXA2 complementation

WT and KO cells were transfected with σNS, µNS, or both expression plasmids in combination with the mCherry-ER-3 plasmid expressing mCherry fused with calreticulin, ER signal peptide, and KDEL (Addgene) using Fugene HD (Promega) according to the manufacturer’s instructions. At 24 h post-transfection, cells were fixed with 4% paraformaldehyde (PFA) in PBS at RT for 20 min. The σNS and µNS proteins were imaged using confocal immunofluorescence microscopy.

To complement ANXA2 in ANXA2 KO cells, we used the ANXA2-GFP (Addgene #107196) and ANXA2 cmamxA2-GFP (Addgene # 107197) plasmids encoding WT ANXA2 and an ANXA2 mutant in which all type II calcium-binding sites are deleted, respectively. The GFP tag was deleted in both plasmids prior to use. Linear fragments of ANXA2 and ANXA2-cmamxA2 flanked by NheI and XhoI restriction enzyme sites were amplified using PCR and primers ANXA2-NheI-F (5′-TAAGCAGCTAGCACCATGTCTACTGTTCACGAAATC-3′) and ANXA2-XhoI-R (5′-TGCTTACTCGAGTCAGTCATCTCCACCACACAGG-3′) to engineer untagged ANXA2 plasmids. PCR fragments were cloned into pcDNA3.1 using NheI (NEB, R3131S) and XhoI (NEB, R0146S).

ANXA2 KO cells were either mock-transfected or transfected with engineered plasmids in combination with the mCherry-ER-3 plasmid using Fugene HD (Promega). At 24 h post-transfection, cells were fixed with 4% PFA in PBS at RT for 20 min and imaged using confocal microscopy.

### Minimum distance analysis

Minimum distance analyses were conducted using the Z-stack analyzer plug-in for FIJI software (75). This method was developed to evaluate the spatial distribution of two fluorescence channels within a specified region of interest. It determines whether these channels are independent or follow the same spatial distribution, going beyond colocalization analysis by identifying dependencies between channels that do not necessarily colocalize. It operates by computing the minimum distance from a particle in one channel (Marker A) to the flipped particles in the other channel (Marker B), simulating a scenario where two channels are independent. The minimum distances calculated in this scenario serve as a baseline for comparison (Fig. S8A). Then the Kolmogorov-Smirnov test is used to compare the minimum distance distributions in the actual and simulated scenarios. The test computes a *P*-value, which denotes the probability that the two distributions are the same. A small P-value indicates significant distribution differences, suggesting a spatial dependency among the channels, even when one channel’s particles are reversed.

WT and KO cells were mock-infected or reovirus-infected, at 14 h post-adsorption, fixed with 4% PFA in PBS at RT for 20 min, and processed for immunofluorescence. From the collected confocal images, the distances between the ER, NS proteins, actin, and ANXA2 fluorescent channels (in groups of two by two) were measured and quantified. The ratio of adjacent signals was calculated by taking all measurements of distances between channels from 0 to 150 nm and dividing by the total measurements (all lengths) (Fig. S8B).

To verify the robustness of the method, minimal measurements were made between the fluorescence channel marking the nucleus and the Golgi in all conditions, as well as measurements between the µNS channel and the Golgi channel in infected cells. In all cases, no significant differences were observed (Fig. S8C and D).

### Quantification and statistical analysis

All experiments were conducted with a minimum of three independent replicates. The data are presented as the mean ± s.e.m. Statistical significance was determined using a two-sample unequal variance t-test with a two-tailed distribution (α = 0.05). Graphs and statistical analyses were conducted using Microsoft Excel.

## Data Availability

The authors declare that the main data supporting the findings of this study are available within the article and its supplemental material. The mass spectrometry proteomics data have been deposited to the ProteomeXchange Consortium via the PRIDE (76) partner repository with the data set identifier PXD067290 and 10.6019/PXD067290.

## SUPPLEMENTAL MATERIAL

File S1 jvi.01389-25-s0001.pdf Raw data corresponding to graphs.

Supplemental figures jvi.01389-25-s0002.pdf Figures S1 to S10.

Video S1 jvi.01389-25-s0003.mp4 3D representation of WT mock-infected HeLa cells.

Video S2 jvi.01389-25-s0004.mp4 3D representation of WT infected HeLa cells.

Video S3 jvi.01389-25-s0005.mp4 3D representation of ANXA2 KO mock-infected HeLa cells.

Video S4 jvi.01389-25-s0006.mp4 3D representation of ANXA2 KO infected HeLa cells.

Video S5 jvi.01389-25-s0007.mp4 3D representation of WT mock-infected HeLa cells.

Video S6 jvi.01389-25-s0008.mp4 3D representation of WT infected HeLa cells.

Video S7 jvi.01389-25-s0009.mp4 3D representation of ANXA2 KO mock-infected HeLa cells.

Video S8 jvi.01389-25-s0010.mp4 3D representation of ANXA2 KO infected HeLa cells.

ASM does not own the copyrights to Supplemental Material that may be linked to, or accessed through, an article. The authors have granted ASM a non-exclusive, world-wide license to publish the Supplemental Material files. Please contact the corresponding author directly for reuse.
