# Supplementary material for: Annexin A2 stabilizes the endoplasmic reticulum and actin cytoskeleton and influences the formation of reovirus factories
Source: J Virol. 2025 Nov 24;99(12):e01389-25. doi: 10.1128/jvi.01389-25 (PMC12724372; doi:10.1128/jvi.01389-25)

FIGURE S2 A: % Cells with VFs
Counting = Infected cells/total cells

R1

14hpi		
	WT	KO
1	0,08	0,1025641
2	0,0952381	0,125
3	0,08571429	0,21428571
4	0,02439024	0,18181818
5	0,05882353	0,24
6	0,04545455	0,0952381
Mean	0,06493678	0,15981768
**%**	**6,49367833**	**15,9817682**

24hpi		
	WT	KO
1	0,2	0,30769231
2	0,25806452	0,18181818
3	0,30232558	0,15625
4	0,10526316	0,125
5	0,13636364	0,18181818
6	0,16129032	0,14285714
Mean	0,19388454	0,18257264
**%**	**19,3884536**	**18,2572636**

R2

14hpi		
	WT	KO
1	0,22807	0,36
2	0,205128	0,301369863
3	0,17284	0,309278351
4	0,155172	0,440677966
5	0,157895	0,301204819
Mean	0,183821	0,3425062
**%**	**18,3821**	**34,25061998**

24hpi		
	WT	KO
1	0,197368	0,289473684
2	0,267606	0,271428571
3	0,112676	0,481927711
4	0,179104	0,481012658
5	0,229508	0,385714286
Mean	0,197253	0,381911382
**%**	**19,72526**	**38,19113821**

Summary

	R1	R2	R3			
	WT	WT	WT	Mean	Std deviatic	Std error
14hpi	6,49367833	18,3821007	8,048494252	10,9747578	6,461881	3,730768936
24hpi	19,3884536	19,7252557	18,61906925	19,2442595	0,567015	0,327366292
	KO	KO	KO	Mean	Std deviatic	Std error
14hpi	15,9817682	34,25062	16,78814056	22,3401763	10,32262	5,959769607
24hpi	18,2572636	38,1911382	34,42177843	30,2900601	10,58977	6,114004743

T student	14hpi	WT vs KO	0,000438299
	24hpi	WT vs KO	0,004400658

All data

R3

14hpi			
		WT	KO
	1	0,11111111	0,19672131
	2	0,05714286	0,17924528
	3	0,06578947	0,15
	4	0,11111111	0,18965517
	5	0,03571429	0,125
	6	0,10204082	0,16666667
Mean		0,08048494	0,16788141
**%**		**8,04849425**	**16,7881406**
24hpi			
		WT	KO
	1	0,15625	0,31428571
	2	0,17808219	0,38028169
	3	0,27419355	0,39655172
	4	0,24691358	0,38461538
	5	0,11666667	0,29411765
	6	0,14503817	0,29545455
Mean		0,18619069	0,34421778
**%**		**18,6190693**	**34,4217784**

14hpi		24hpi	
WT	KO	WT	KO
0,08	0,1025641	0,2	0,307692308
0,095238095	0,125	0,258065	0,181818182
0,085714286	0,21428571	0,302326	0,15625
0,024390244	0,18181818	0,105263	0,125
0,058823529	0,24	0,136364	0,181818182
0,045454545	0,0952381	0,16129	0,142857143
0,0625	0,125	0,242424	0,133333333
0,051724138	0,10810811	0,102564	0,083333333
0,108108108	0,10344828	0,258065	0,235294118
0,068181818	0,08571429	0,108696	0,625
0,228070175	0,36	0,197368	0,289473684
0,205128205	0,30136986	0,267606	0,271428571
0,172839506	0,30927835	0,112676	0,481927711
0,155172414	0,44067797	0,179104	0,481012658
0,157894737	0,30120482	0,229508	0,385714286
0,111111111	0,19672131	0,15625	0,314285714
0,057142857	0,17924528	0,178082	0,38028169
0,065789474	0,15	0,274194	0,396551724
0,111111111	0,18965517	0,246914	0,384615385
0,035714286	0,125	0,116667	0,294117647
0,102040816	0,16666667	0,145038	0,295454545

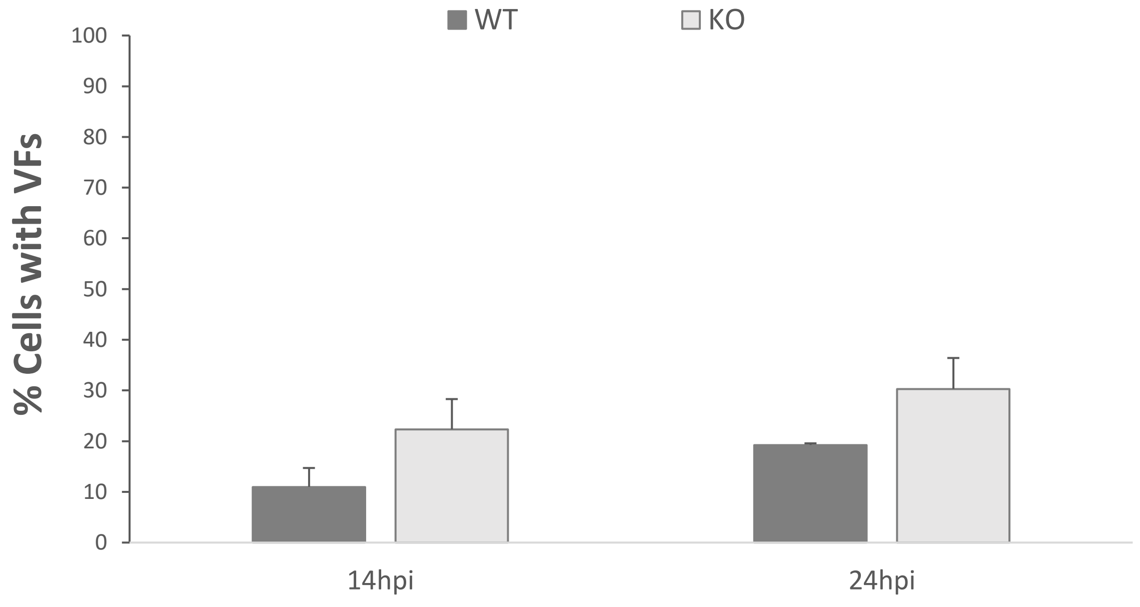

FIGURE 2B: WB µNS/Tomm22

						µNS/Tomm22
R1	µNS	14hpi	MWT14	2.736.523.972.817	273,652397	1,32385155
			MKO14	2.714.015.055.824	271,401506	1,48234538
		24hpi	MWT24	2.705.556.290.840	270,555629	1,91586717
			MKO24	2.394.230.728.746	239,423073	2,137163
	Tomm22	14hpi	TWT14h	2.067.092.771.940	206,709277	
			TKO14h	1.830.892.512.419	183,089251	
		24hpi	TWT24h	1.412.183.645.693	141,218365	
			TKO24h	1.120.284.569.284	112,028457	
			b	0.0000000000		
						µNS/Tomm22
R2	µNS	14hpi	MWT14	10673956888673	106,739569	0,6680605
			MKO14	13635886377082	136,358864	0,66796007
		24hpi	MWT24	21959812180334,000	219,598122	0,83752085
			MKO24	34327672450287,000	343,276725	1,12894914
	Tomm22	14hpi	TWT14h	15977530382706	159,775304	
			TKO14h	20414223911025	204,142239	
		24hpi	TWT24h	26220018610696,000	262,200186	
			TKO24h	30406748487279,000	304,067485	
			b	0.0000000000		
						µNS/Tomm22
R3	µNS	14hpi	MWT14	2.007.164.572.559	200,716457	0,23487568
			MKO14	4.670.098.484.752	467,009848	0,38016654
		24hpi	MWT24	9.358.978.295.148	935,89783	1,04178869
			MKO24	14.119.399.185.666	1411,93992	1,04661704
	Tomm22	14hpi	TWT14h	8.545.646.536.568	854,564654	
			TKO14h	12.284.348.975.204	1228,4349	
		24hpi	TWT24h	8.983.566.835.631	898,356684	
			TKO24h	13.490.511.514.414	1349,05115	
			b	0.0000000000		

Summary

		R1	R2	R3	Mean	Std deviation	Std error
WT	14hpi	1,323852	0,66806	0,234875684	0,74226258	0,54826688	0,31654203
KO	14hpi	1,482345	0,66796	0,380166543	0,84349066	0,57167105	0,33005443
WT	24hpi	1,915867	0,837521	1,041788687	1,2650589	0,57279568	0,33070374
KO	24hpi	2,137163	1,128949	1,046617037	1,43757639	0,60725671	0,35059982

			p value
14hpi	T-student	WT vs KO	0,835675
24hpi		WT vs KO	0,41793

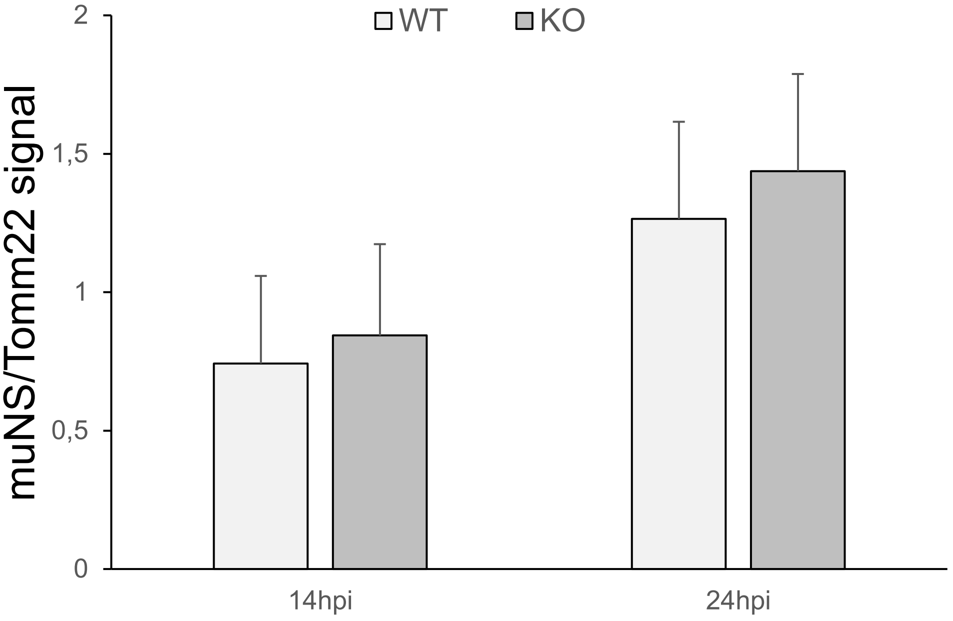

FIGURE 2C: Extracellular titration

Extracellular Viral titer		R1	R2	R3	Mean	Std deviation	Std error
0 hpi	WT	17000	34000	12000	21000,00	11532,56259	6658,32812
	KO	40000	6000	35000	27000,00	18357,55975	10598,7421
14 hpi	WT	150000	180000	140000	156666,67	20816,65999	12018,5043
	KO	260000	220000	150000	210000,00	55677,64363	32145,5025
24 hpi	WT	700000	700000	730000	710000,00	17320,50808	10000
	KO	5E+06	4E+06	3300000	4133333,33	1040833	600925,213

Extracellular Titration			p value
T student	14hpi	WT vs KO	0,23352987
	24hpi	WT vs KO	0,02942959

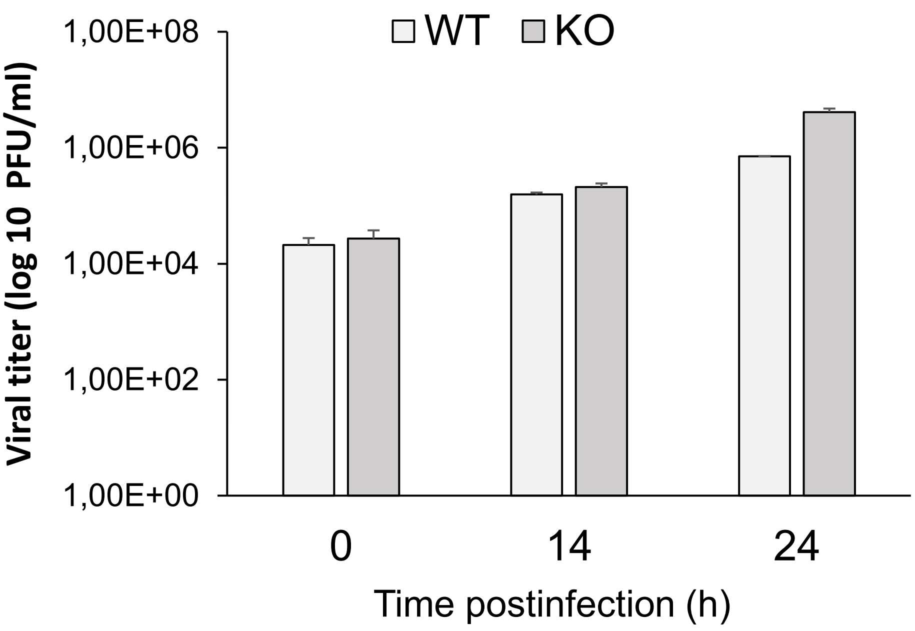

FIGURE 2D: Intracellular titration

Intracellular Viral titer		R1	R2	R3	Mean	Std deviation	Std error
0 hpi	WT	17000	17000	12000	15333,33	2886,751346	1666,66667
	KO	40000	30000	35000	35000,00	5000	2886,75135
14 hpi	WT	11000000	4950000	6000000	7316666,67	3232774,866	1866443,44
	KO	5500000	12500000	4500000	7500000,00	4358898,944	2516611,48
24 hpi	WT	25000000	5000000	40000000	23333333,33	17559422,92	10137937,6
	KO	55000000	21000000	45000000	40333333,33	10088497,3	5824596,63

Intracellular Titration			p value
T student	14hpi	WT vs KO	0,956370354
	24hpi	WT vs KO	0,300334419

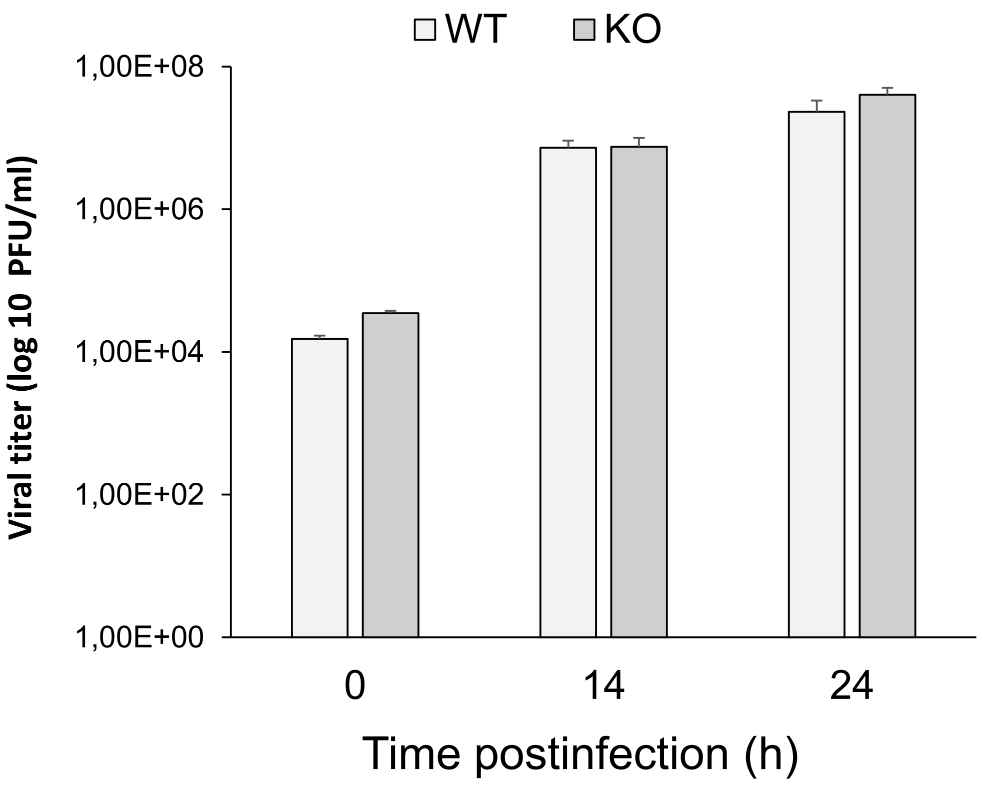

FIGURE 2E: % Infected cell in Early times postinfection
Counting infected cells with early/advanced infection

R1	infected cells/total cells						
WT 7hpi	KO 7hpi	WT 8hpi	KO 8hpi	WT 9hpi	KO 9hpi	WT 10hpi	KO 10hpi
0,000000	0,0000000	0,007422	0,019349	0,0000000	7,99E-02	0,02543783	0,034576449
0,019643	0,0000000	0,011007	0,016985	0,1776236	1,02E-02	0,01110866	0,028377161
0,009292	0,0104132	0,015416	0,023216	0,0000000	4,72E-02	0,02773512	0,027441828
0,000000	0,0000000	0,005401	0,000000	0,0240093	3,25E-02	0,01939722	0,026503878
0,000000	0,0155783	0,000000	0,018815	0,0000000	1,97E-02	0,02026897	0,093670946
0,021029	0,0158507	0,012163	0,014805	0,0206099	2,99E-02	0,02005911	0,086149357
0,000000	0,0196529	0,000000	0,012003	0,0181877	8,03E-02	0,02784862	0,092806034
0,000000	0,0148492	0,012031	0,000000	0,0639695	4,76E-02	0,02176035	0,055848827
0,000000	0,0157568	0,012148	0,000000	0,0000000	3,25E-02	0,02797472	0,196445448
0,000000	0,0466192	0,000000	0,027181	0,0000000	5,65E-02	0,01885369	0,031261811
0,000000	0,0537964	0,000000	0,000000	0,0245291	6,48E-02	0,06906010	0,046860754
0,000000	0,0000000	0,000000	0,008539		6,51E-02	0,01045579	0,09133992
0,000000	0,0171177	0,008222	0,038341	0,0241200	4,62E-02	0,02117509	0,063440603
0,000000	0,0317121	0,014681	0,000000	0,0218072	2,47E-02	0,03884584	0,069530702
0,000000	0,0099368	0,000000	0,008376	0,0155114	0,006765561	0,01470983	0,112711562
0,000000	0,0070786	0,006263	0,000000	0,0304404	0,017948939	0,02776557	0,113431932
0,000000	0,0000000	0,023669	0,022284	0,0329117	0,024049469	0,03046671	0,0848873
0,000000	0,0000000	0,000000	0,032493	0,0184565	0,038683252	0,02445211	0,099014382
0,000000	0,0052463	0,011991	0,070605	0,0336015	4,84E-02	0,02274068	0,075616548
0,005115	0,0096272	0,005704	0,020780	0,0113429	5,72E-02	0,01060817	0,120247343
		0,006646	0,052678	0,0281516	1,88E-02	0,00895338	0,131004864
		0,000000	0,025057	0,0068937	4,54E-02	0,01253892	0,092090846
		0,012226	0,000000	0,0367789	8,20E-02	0,02774893	0,083767887
		0,012449	0,000000	0,0181399	2,46E-02	0,03827862	0,085989891
		0,005702	0,016865	0,0372142	3,70E-02	0,03816105	0,092633746
		0,000000	0,000000	0,0271786	4,79E-02	0,06010103	0,119783704
		0,009168	0,007942	0,0113154	5,10E-02	0,02844668	0,066030918
		0,000000	0,012560	0,0288145	4,93E-02	0,03237826	0,083238567
		0,006452	0,032884	0,0298562	1,53E-02	0,01819756	0,060723948
		0,000000	0,000000	0,0522017	2,61E-02	0,10440346	0,065252163

R2	infected cells/total cells						
0,00000000	0	0,019713	0,022626	0,80421728	0,112745545	0,047079425	0,14019289
0,00949786	0	0,043996	0,045955	0,41843285	0,079854237	0,055147337	0,190841222
0,00000000	0,009258	0,027142	0,050255	0,33818791	0,024395922	0,065245038	0,072137181
0,00000000	0,008102	0,06364	0,014373	0,95191151	0,078264294	0,031049454	0,082218035
0,00000000	0,007682	0,027235	0,012093	0,57032848	0,070594909	0,024391024	0,123779387
0,00000000	0	0,05992	0	0,83610055	0,133589658	0,056464362	0,159777025
0,00863629	0,009463	0,057962	0,06467	0,71758401	0,104889691	0,079508383	0,146170607
0,00000000	0,007911	0,034503	0,031096	0,3125611	0,049685194	0,109115486	0,158961539
0,00000000	0,00846	0,046876	0,051295	0,49358872	0,092754033	0,046231599	0,18791765
0,00879468	0,011785	0,010962	0,021882	0,55353031	0,135007682	0,044543384	0,243902962
0,00000000	0	0,02432	0	1,1400801	0,134882563	0,091306902	0,118309725
0,00000000	0	0,015022	0,015146	1,20265646	0,174192674	0,089488574	0,144839927
0,00844499	0,012794	0,009692	0,007218	0,5337421	0,046974294	0,092772673	0,088009347
0,00000000	0	0,014734	0,008678	0,64226396	0,107116736	0,07928058	0,166779928
0,00862138	0	0,017323	0,009346	1,14996035	0,108812287	0,041392973	0,094622642
0,00000000	0	0	0,039758	0,3996229	0,079053268	0,067414994	0,197819664
0,00787649	0	0,059552	0,036019	1,485106	0,09477603	0,033852206	0,063817687
0,00000000	0	0,090471	0	0,87977668	0,133740967	0,079444362	0,152016948
0,00000000	0	0,021687	0,013886	0,15824959	0,018336317	0,050360476	0,115869599
0,00000000	0	0,019859	0,029934	0,3487388	0,078106147	0,058941955	0,223967471

R3	infected cells/total cells						
0	0,000000	0	0,013307	0	0,059310972	0,246634826	0,090636649
0	0,000000	0,019726	0,049126	0,01805775	0,058059877	0,148695132	0,058326114
0	0,000000	0,011535	0,043081	0,01583084	0,102502149	0,380411703	0,188040507
0	0,000000	0,010353	0,050135	0,02610678	0,094907247	0,322360884	0,082674603
0	0,000000	0,028009	0,057102	0,01748752	0,072296162	0,756757228	0,076326704
0	0,074113	0,014853	0,073184	0,01924765	0,122483695	0,539184885	0,166362465
0	0,000000	0,012067	0,053325	0,03676138	0,158453615	0,634915846	0,113136542
0	0,000000	0,02103	0	0,02208238	0,148576783	0,351335468	0,083356172
0	0,000000	0,022852	0,039822	0,02021324	0,170671524	1,045380506	0,105986196
0	0,000000	0	0,058599	0	0,038789964	0,128173151	0,07871412
0	0,000000	0	0,053016	0,0303902	0,11977813	0,396085394	0,134842886
0	0,000000	0	0,016837	0,02026865	0,116368074	0,630626661	0,215170315
0	0,075815	0	0,018575	0,01221053	0,057817001	0,720839378	0,148375905
0,01055385	0,095808	0	0	0,01158866	0,160694829	0,416900902	0,108703061
0	0,000000	0	0,055587	0	0,034484581	1,191169037	0,038679836
0	0,000000	0	0,017635	0,0104082	0,075412494	0,573410799	0,050863027
0	0,000000	0,011759	0,063107	0	0,041822879	1,330153195	0,124024784
0	0,000000	0	0,021615	0	0,036346257	0,651337784	0,08189404
0,01411512	0,047363	0,010921	0,013156	0,00977743	0,096445109	0,652600408	0,26256062
0,01601544	0,036440	0	0,019495	0	0,07060207	0,536896461	0,114686565

		p value		
**T student:**	7 hpi	8,60E-04	0,00086	***
WT vs KO	8 hpi	5,83E-03	0,00583	**
	9 hpi	1,27E-03	0,00127	**
	10 hpi	1,82E-02	0,01820	*

R1

Infected cells	WT 7hpi	KO 7hpi	WT 8hpi	KO 8hpi	WT 9hpi	KO 9hpi	WT 10hpi	KO 10hpi
Early infected	6	24	32	42	60	79	91	215
Advan. Infected	0	0	1	2	4	10	39	55
Total	6	24	33	44	64	89	130	270
Total cells	2512	2435	4947	2999	2891	2396	4673	3172
% INF EARLY	0,24	0,99	0,65	1,40	2,08	3,30	1,95	6,78
% INF ADV	0,00	0,00	0,02	0,07	0,14	0,42	0,83	1,73
% total	0,24	0,99	0,67	1,47	2,21	3,71	2,78	8,51

R2

Infected cells	WT 7hpi	KO 7hpi	WT 8hpi	KO 8hpi	WT 9hpi	KO 9hpi	WT 10hpi	KO 10hpi
Early infected	6	10	8	29	23	73	50	64
Advan. Infected	0	0	4	14	16	28	45	67
Total	6	10	12	43	39	101	95	131
Total cells	2355	932	1844	1366	1831	1071	1582	922
% INF EARLY	0,25	1,07	0,43	2,12	1,26	6,82	3,16	6,94
% INF ADV	0,00	0,00	0,22	1,03	0,87	2,62	2,84	7,27
% total	0,25	1,07	0,65	3,15	2,13	9,43	6,00	14,21

R3

Infected cells	WT 7hpi	KO 7hpi	WT 8hpi	KO 8hpi	WT 9hpi	KO 9hpi	WT 10hpi	KO 10hpi
Early infected	3	11	13	29	24	44	26	42
Advan. Infected	0	0	2	12	9	26	27	40
Total	3	11	15	41	33	70	53	82
Total cells	1659	765	1972	1137	2289	865	1268	728
% INF EARLY	0,18	1,44	0,66	2,55	1,05	5,09	2,05	5,77
% INF ADV	0,00	0,00	0,10	1,06	0,39	3,01	2,13	5,50
% total	0,18	1,44	0,76	3,61	1,44	8,09	4,18	11,27

**Summary**	WT 7 hpi	KO 7 hpi	WT 8 hpi	KO 8 hpi	WT 9 hpi	KO 9 hpi	WT 10 hpi	KO 10 hpi
Mean Early	0,22	1,17	0,58	2,02	1,46	5,07	2,39	6,50
Mean Advanced	0,00	0,00	0,11	0,72	0,47	2,01	1,94	4,83
**Mean Total**	**0,22**	**1,17**	**0,69**	**2,74**	**1,93**	**7,08**	**4,32**	**11,33**
Std Dev total	0,04	0,24	0,06	1,13	0,42	2,99	1,62	2,85
Std Error total	0,02	0,14	0,03	0,65	0,24	1,73	0,93	1,65

Early-infected WT
Advanced-infected WT
Early-infected KO Early-infected (< 5 VFs / cell)
Advanced-infected Advanced-infected (≥ 5VFs / cell)

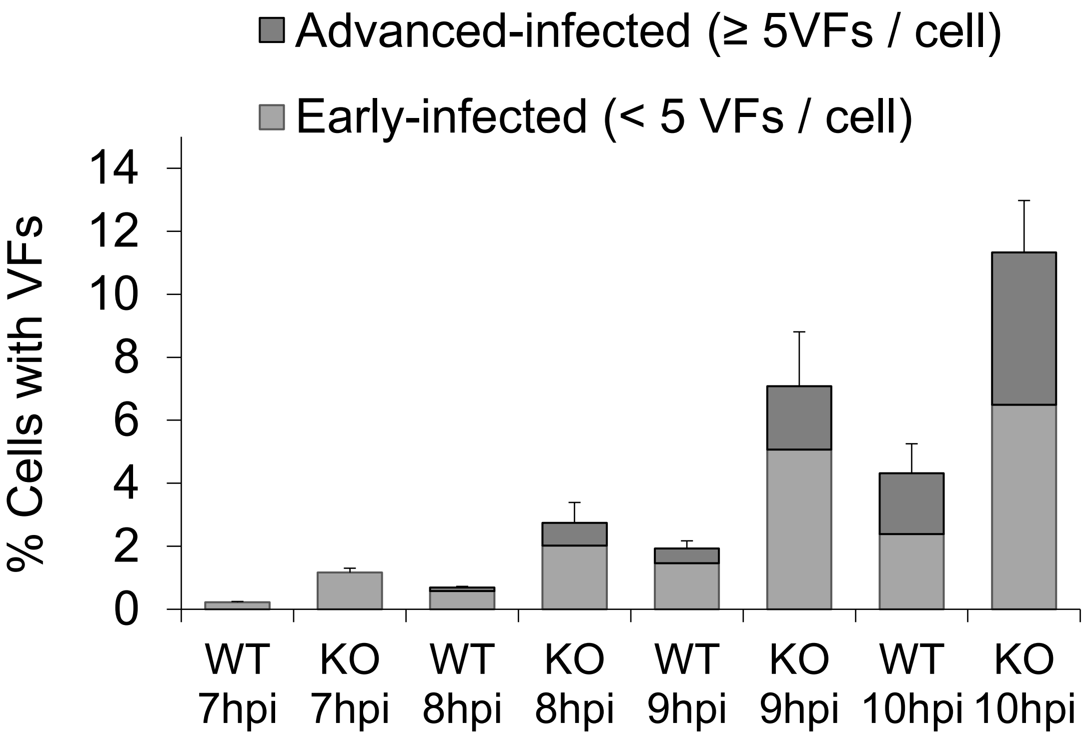

Advanced-infected (≥ 5VFs / cell)
Early-infected (< 5 VFs / cell)
% Cells with VFs
0
2
4
6
8
10
12
14
WT 7hpi
KO 7hpi
WT 8hpi
KO 8hpi
WT 9hpi
KO 9hpi
WT 10hpi
KO 10hpi

FIGURE 2F: WB Early Times Postinfection
WB σNS/GAPDH

				σNS/GAPDH	
R1					
σNS	WT7	2.737.055.632.000	273,7055632	1,29229436	1,29229436
	KO7	3.061.284.395.397	306,1284395	1,38392886	1,38392886
	WT8	2.137.056.794.183	213,7056794	0,86248098	0,86248098
	KO8	2.208.597.874.305	220,8597874	0,96021392	0,96021392
	WT9	2.779.564.510.749	277,9564511	1,11674001	1,11674001
	KO9	2.317.271.499.019	231,7271499	1,02570449	1,02570449
	WT10	2.351.393.296.753	235,1393297	0,97726799	0,97726799
	KO10	3.647.734.872.830	364,7734873	1,71477097	1,71477097
	B	0.0000000000			
GAPDH	WT7	21.179.815.622.989	211,7981562		
	KO7	22.120.243.909.682	221,2024391		
	WT8	24.778.016.541.546	247,7801654		
	KO8	23.001.102.419.239	230,0110242		
	WT9	24.889.987.650.915	248,8998765		
	KO9	22.591.999.268.682	225,9199927		
	WT10	24.060.885.313.550	240,6088531		
	KO10	21.272.431.931.123	212,7243193		
	B	0.0000000000			

				σNS/GAPDH	
R2					
σNS	WT7	983.080.533.860	98,30805339	0,41625691	0,41625691
	KO7	1.762.362.179.156	176,2362179	1,00909147	1,00909147
	WT8	1.115.840.133.445	111,5840133	0,57131322	0,57131322
	KO8	1.654.190.345.585	165,4190346	0,78227972	0,78227972
	WT9	1.390.319.888.742	139,0319889	0,66736173	0,66736173
	KO9	685.446.701.529	68,54467015	0,45932509	0,45932509
	WT10	1.469.387.836.011	146,9387836	0,6377028	0,6377028
	KO10	1.647.738.745.173	164,7738745	0,96760343	0,96760343
	B	0.0000000000			
GAPDH	WT7	23.617.158.574.117	236,1715857		
	KO7	17.464.840.736.978	174,6484074		
	WT8	19.531.144.979.979	195,3114498		
	KO8	21.145.765.509.704	211,4576551		
	WT9	20.833.077.943.079	208,3307794		
	KO9	14.922.910.174.731	149,2291017		
	WT10	23.041.890.870.731	230,4189087		
	KO10	17.029.070.971.386	170,2907097		
	B	0.0000000000			

R3				σNS/GAPDH	
σNS	WT7	501.289.351.997	50,1289352	0,42145294	0,42145294
	KO7	427.669.422.853	42,76694229	0,42376373	0,42376373
	WT8	929.532.250.442	92,95322504	0,84504748	0,84504748
	KO8	1.359.280.522.317	135,9280522	1,06174969	1,06174969
	WT9	1.711.036.113.657	171,1036114	1,32387117	1,32387117
	KO9	1.601.573.960.004	160,157396	1,25760603	1,25760603
	WT10	2.568.382.123.936	256,8382124	2,04937396	2,04937396
	KO10	2.755.478.535.878	275,5478536	3,33203308	3,33203308
	B	0.0000000000			
GAPDH	WT7	11.894.313.888.086	118,9431389		
	KO7	10.092.166.839.724	100,9216684		
	WT8	10.999.763.648.766	109,9976365		
	KO8	12.802.269.119.373	128,0226912		
	WT9	12.924.491.104.952	129,244911		
	KO9	12.735.100.790.641	127,3510079		
	WT10	12.532.520.537.711	125,3252054		
	KO10	8.269.661.407.841	82,69661408		
	B	0.0000000000			

Summary

	CTL7	KO7	CTL8	KO8	CTL9	KO9	CTL10	KO10
R1	1,292294	1,38393	0,86248	0,96021	1,11674	1,0257	0,97727	1,714771
R2	0,416257	1,00909	0,57131	0,78228	0,66736	0,45933	0,6377	0,967603
R3	0,421453	0,42376	0,84505	1,06175	1,32387	1,25761	2,04937	3,332033
Mean	0,710001	0,93893	0,75961	0,93475	1,03599	0,91421	1,22145	2,004802
Std deviation	0,504287	0,48391	0,16331	0,14146	0,33562	0,41065	0,73683	1,208603
Std error	0,29115	0,27939	0,09428	0,08167	0,19377	0,23709	0,42541	0,697787

T test WT vs KO	P value
7 hpi	0,600864
8 hpi	0,234373
9 hpi	0,711918
10 hpi	0,402513

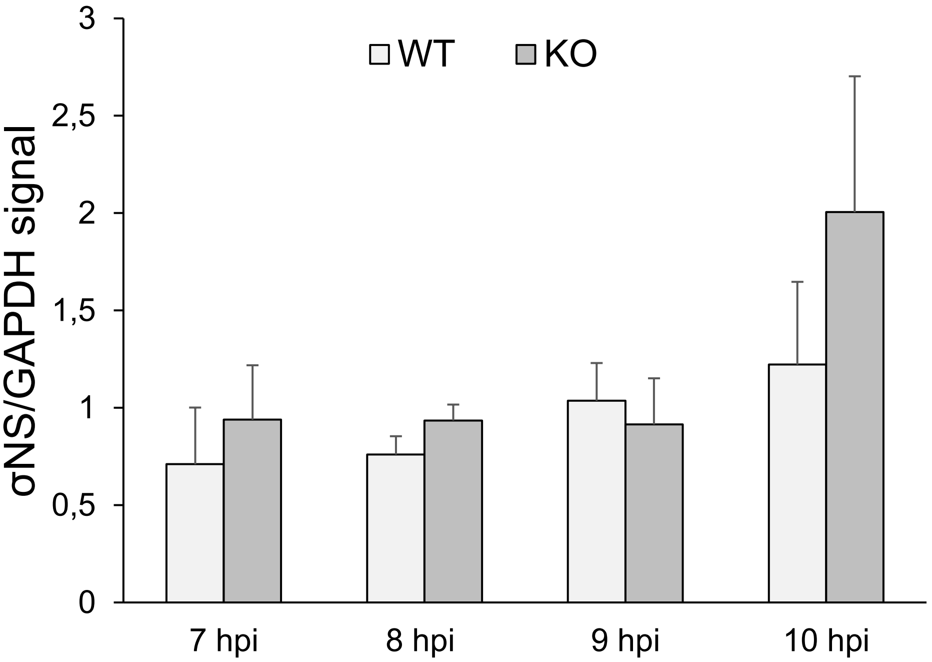

FIGURE 3F: % Cells with the main ER morphologies

		Raw			
ER morphologies	Samples	R1	R2	R3	R4
Normal ER	Mock-infected WT cells	10	10	15	11
	Mock-infected ANXA2 KO cells	0	0	1	6
	14hpi WT cells	0	0	0	0
	14hpi ANXA2 KO cells	0	0	0	0
Fragmented ER	Mock-infected WT cells	0	0	0	0
	Mock-infected ANXA2 KO cells	5	10	9	16
	14hpi WT cells	1	5	5	12
	14hpi ANXA2 KO cells	7	5	2	23
Unbranched ER	Mock-infected WT cells	0	0	1	0
	Mock-infected ANXA2 KO cells	0	1	0	0
	14hpi WT cells	1	6	5	3
	14hpi ANXA2 KO cells	4	4	3	15
Collapsed ER	Mock-infected WT cells	5	5	7	5
	Mock-infected ANXA2 KO cells	1	11	11	22
	14hpi WT cells	4	10	8	18
	14hpi ANXA2 KO cells	7	5	4	25

						Total
Num cells	Mock-infected WT cells	10	10	15	11	46
	Mock-infected ANXA2 KO cells	5	11	11	22	49
	14hpi WT cells	5	11	10	20	46
	14hpi ANXA2 KO cells	7	5	4	25	41

%

R1	R2	R3	R4	Mean	Std d	Std error
100	100	100	100	100,000	0	0,00
0	0	9,091	27	9,091	13	6,43
0	0	0	0	0	0	0,00
0	0	0	0	0	0	0,00
0	0	0	0	0	0	0,00
100	90,90909091	81,82	73	86,364	12	5,87
20	45,45454545	50	60	43,864	17	8,51
100	100	50	92	85,500	24	11,98
0	0	6,667	0	1,667	3,3	1,67
0	9,090909091	0	0	2,273	4,5	2,27
20	54,54545455	50	15	34,886	20	10,13
57,14285714	80	75	60	68,036	11	5,59
50	50	46,67	45	48,030	2,3	1,16
20	100	100	100	80,000	40	20,00
80	90,90909091	80	90	85,227	6	3,02
100	100	100	100	100,000	0	0,00

Summary:

		Normal ER	Fragmented ER	Unbranched ER	Collapsed ER
Mock-infected	WT	100,00	0,00	1,67	48,03
	ANXA2 KO	9,09	86,36	2,27	80,00
Infected	WT	0,00	43,86	34,89	85,23
	ANXA2 KO	0,00	85,50	68,04	100,00

	ER MORPHOLOGY			
	Normal ER	Unbranched ER	Fragmented ER	Collapsed ER
Mock-infected WT cells	46 / 100%	1 / 2.17%	0 / 0%	22 / 47.83%
Mock-infected ANXA2 KO cells	7 / 14.29%	1 / 2.04%	40 / 81.63%	45 / 91.84%
14 hpi WT cells	0 / 0%	15 / 32.61%	23 / 50%	40 / 86.96%
14 hpi ANXA2 KO cells	0 / 0%	26 / 63.41%	37 / 90.24%	41 / 100%

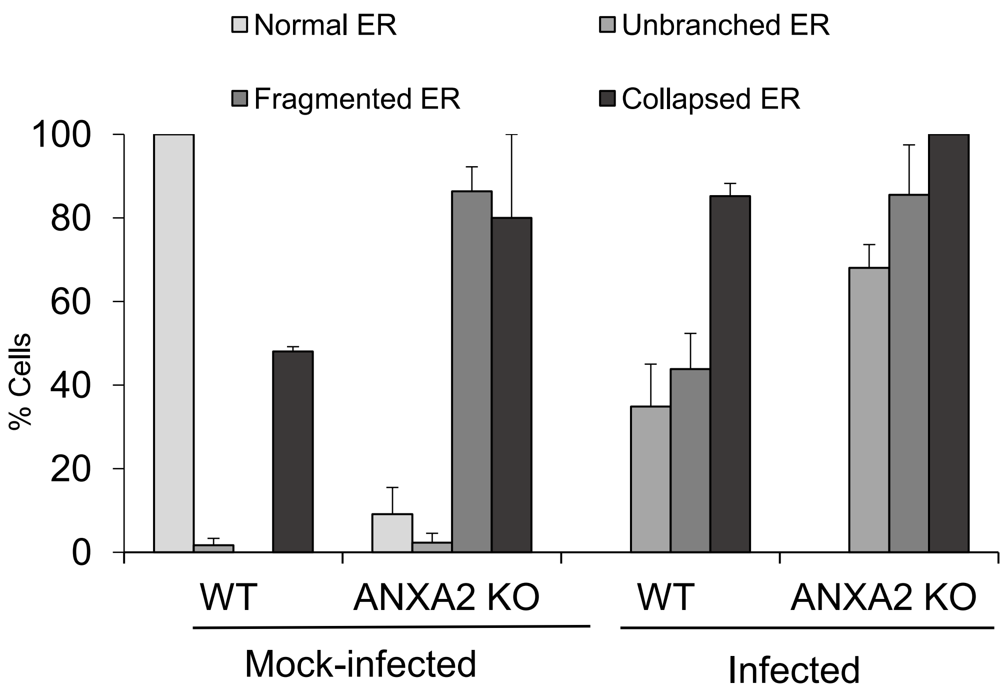

FIGURE 6A: Minimum distances
Between ER & ANXA2

	ch1	R	ANXA2
	ch2	G	sigmaNS
	ch3	B	ER

**WT NI**	ER & sigmaNS	
	Ch3 & Ch1	Number of measurements
	Total all distances measured	14284
	Distances 0-150 nm	3281
	Total all distances measured	61786
	Distances 0-150 nm	39919
	Total all distances measured	73386
	Distances 0-150 nm	42877
	Total all distances measured	50572
	Distances 0-150 nm	26302
	Total all distances measured	74619
	Distances 0-150 nm	28559
	Total all distances measured	108939
	Distances 0-150 nm	46794
	Total all distances measured	62589
	Distances 0-150 nm	11840
	Total all distances measured	48090
	Distances 0-150 nm	13080
	Total all distances measured	60581
	Distances 0-150 nm	15622

**WT 14**	ER & sigmaNS	
	Ch3 & Ch1	Number of measurements
	Total all distances measured	214341
	Distances 0-150 nm	41862
	Total all distances measured	100047
	Distances 0-150 nm	28595
	Total all distances measured	282862
	Distances 0-150 nm	56535
	Total all distances measured	188882
	Distances 0-150 nm	63990
	Total all distances measured	114849
	Distances 0-150 nm	29321
	Total all distances measured	117441
	Distances 0-150 nm	35135
	Total all distances measured	42578
	Distances 0-150 nm	10840
	Total all distances measured	92857
	Distances 0-150 nm	29015
	Total all distances measured	65722
	Distances 0-150 nm	8900

**Summary:**	**ER & ANXA2**	
	% Adjacent signal	
	WT NI	WT 14hpi
	22,96975637	19,5305611
	64,60848736	28,5815667
	58,42667539	19,986778
	52,00901685	33,8782944
	38,27309398	25,5300438
	42,95431388	29,9171499
	18,9170621	25,4591573
	27,19900187	31,2469711
	25,78696291	13,5418886
Mean	**39,01604119**	**25,2969345**
Std Deviation	16,55569595	6,52771455
Std error	5,853322436	2,17590485

T test:	WTNI vs WT14	P value
	ER & ANXA2	0,04230702

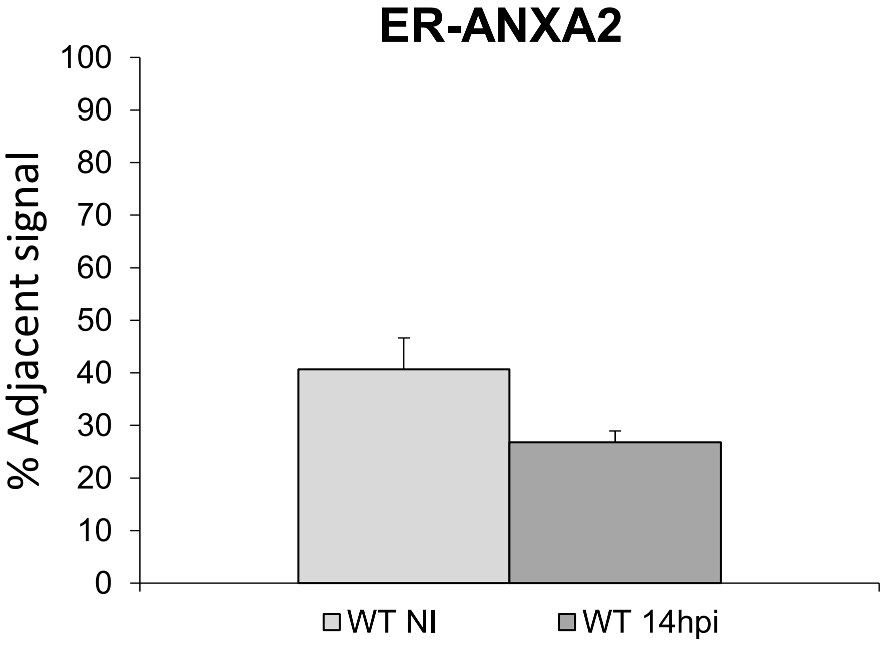

FIGURE 6B: Minimum distances

	Between actin and ANXA2		
	ch1	R	ANXA2
	ch2	G	sigmaNS
	ch3	B	Act
**WT 14**	ACT & ANXA2		
	Ch3 & Ch1	Number of measurements	
	Total all distances measured	39277	
	Distances 0-150 nm	6430	
	Total all distances measured	19141	
	Distances 0-150 nm	2776	
	Total all distances measured	44602	
	Distances 0-150 nm	5693	
	Total all distances measured	35608	
	Distances 0-150 nm	4852	
	Total all distances measured	24749	
	Distances 0-150 nm	4228	
	Total all distances measured	87887	
	Distances 0-150 nm	8362	
	Total all distances measured	68135	
	Distances 0-150 nm	9779	
	Total all distances measured	42735	
	Distances 0-150 nm	7143	
	Total all distances measured	8011	
	Distances 0-150 nm	1329	
	Total all distances measured	53774	
	Distances 0-150 nm	5742	
**WT NI**	ACT & ANXA2		
	Ch3 & Ch1	Number of measurements	
	Total all distances measured	163274	
	Distances 0-150 nm	33438	
	Total all distances measured	270564	
	Distances 0-150 nm	32273	
	Total all distances measured	161780	
	Distances 0-150 nm	29396	
	Total all distances measured	143045	
	Distances 0-150 nm	28878	
	Total all distances measured	262434	
	Distances 0-150 nm	33834	
	Total all distances measured	114929	
	Distances 0-150 nm	23743	
	Total all distances measured	87231	
	Distances 0-150 nm	15155	
	Total all distances measured	135493	
	Distances 0-150 nm	21883	
	Total all distances measured	54104	
	Distances 0-150 nm	12202	
	Total all distances measured	111510	
	Distances 0-150 nm	22365	

**Summary:**	**ACT & ANXA2**	
	% Adjacent signal	
	WT14	KO14
	16,37090409	20,4796845
	14,50289954	11,9280466
	12,76400161	18,1703548
	13,62615143	20,1880527
	17,08351853	12,8923844
	9,514490198	20,6588415
	14,35238864	17,3734108
	16,71463671	16,1506498
	16,58968918	22,5528612
	10,67802284	20,0564972
Mean	**14,61318666**	**18,0450783**
Std Deviation	2,623024884	3,49191668
Std error	0,829473299	1,10424101

T test:	WT14 vs WT NI	P value
	ACT & ANXA2	0,01326613

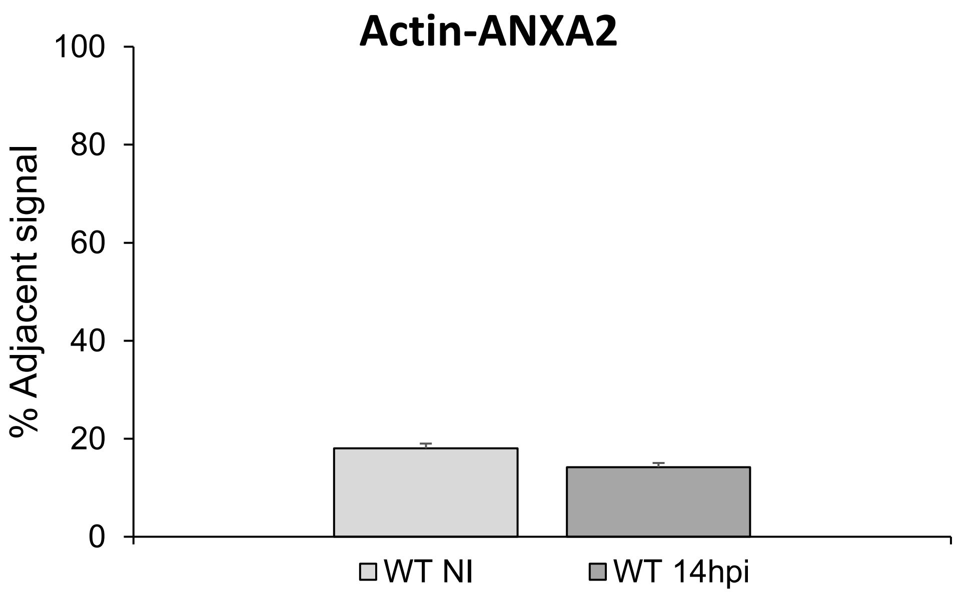

FIGURE 6C: Minimum distances

	Between ANXA2 and muNS/sigmaNS		
ch1	R	ANXA2	
ch2	G	sigmaNS	
ch3	B	ER	
**WT 14**	ANXA2 & sigmaNS		
	Ch1 & Ch2	Number of measurements	
	Total all distances measured		5458
	Distances 0-150 nm		3818
	Total all distances measured		11280
	Distances 0-150 nm		8494
	Total all distances measured		2301
	Distances 0-150 nm		1491
	Total all distances measured		2891
	Distances 0-150 nm		2009
	Total all distances measured		43553
	Distances 0-150 nm		21574
	Total all distances measured		46017
	Distances 0-150 nm		25685
	Total all distances measured		2790
	Distances 0-150 nm		1015
	Total all distances measured		8584
	Distances 0-150 nm		4117
	Total all distances measured		30529
	Distances 0-150 nm		11511
	Total all distances measured		473
	Distances 0-150 nm		139
ch1	R	muNS	
ch2	G	ANXA2	
ch3	B	ER	
**WT 14**	ANXA2 & muNS		
	Ch1 & Ch2		
	Total all distances measured		1427
	Distances 0-150 nm		391
	Total all distances measured		55672
	Distances 0-150 nm		32905
	Total all distances measured		194058
	Distances 0-150 nm		101173
	Total all distances measured		173932
	Distances 0-150 nm		88589
	Total all distances measured		81886
	Distances 0-150 nm		41596
	Total all distances measured		29339
	Distances 0-150 nm		10842
	Total all distances measured		33665
	Distances 0-150 nm		11274

**Summary:**	**ANXA2 & muNS/sigmaNS**	
	% Adjacent signal	
	ANXA2-muNS	ANXA2-sigmaNS
	WT14	WT14
	27,40014015	69,9523635
	59,10511568	75,3014184
	52,13544404	64,797914
	50,93312329	69,4915254
	50,79745011	49,5350493
	36,95422475	55,8163287
	33,48878657	36,3799283
		47,9613234
		37,7051328
Mean	**44,40204066**	**56,326776**
Std Deviation	11,71306921	14,3691324
Std error	4,427124033	4,7897108
T test:	WT14	P value
	ACT & ANXA2 & ANXA2-sigmaNS	0,08897868
		T-test:

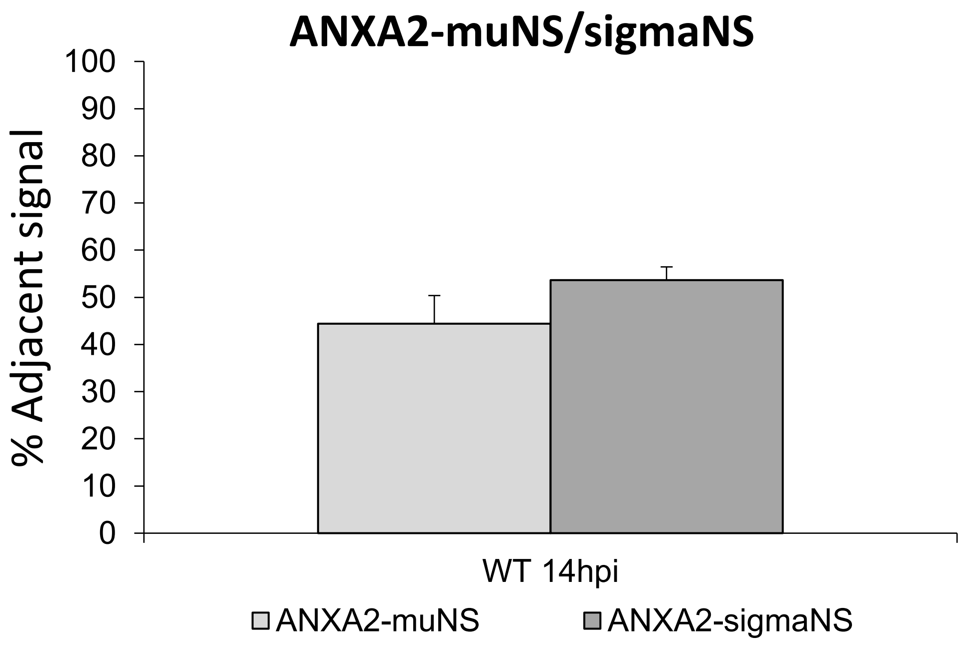

FIGURE 6D: Minimum distances

	Between ER and actin		
	ch1	R	muNS
	ch2	G	ER
	ch3	B	Act
**WT NI**	ER & actin		
	Ch2 & Ch3	Number of measurements	
	Total all distances measured	17808	
	Distances 0-150 nm	4264	
	Total all distances measured	25947	
	Distances 0-150 nm	6150	
	Total all distances measured	18341	
	Distances 0-150 nm	5966	
	Total all distances measured	60796	
	Distances 0-150 nm	4024	
	Total all distances measured	46797	
	Distances 0-150 nm	7252	
	Total all distances measured	83515	
	Distances 0-150 nm	16882	
	Total all distances measured	53013	
	Distances 0-150 nm	10621	
	Total all distances measured	49466	
	Distances 0-150 nm	10821	
	Total all distances measured	112903	
	Distances 0-150 nm	10413	
	Total all distances measured	54153	
	Distances 0-150 nm	6154	
**KO NI**	ER & actin		
	Ch2 & Ch3	Number of measurements	
	Total all distances measured	29527	
	Distances 0-150 nm	16086	
	Total all distances measured	15382	
	Distances 0-150 nm	9652	
	Total all distances measured	8210	
	Distances 0-150 nm	2650	
	Total all distances measured	9429	
	Distances 0-150 nm	5996	
	Total all distances measured	122914	
	Distances 0-150 nm	34752	
	Total all distances measured	37191	
	Distances 0-150 nm	21483	
	Total all distances measured	27112	
	Distances 0-150 nm	10999	
	Total all distances measured	27600	
	Distances 0-150 nm	8593	
	Total all distances measured	28145	
	Distances 0-150 nm	10307	
	Total all distances measured	34683	
	Distances 0-150 nm	7629	

**WT 14**	ER & actin	
	Ch2 & Ch3	Number of measurements
	Total all distances measured	44340
	Distances 0-150 nm	15074
	Total all distances measured	38415
	Distances 0-150 nm	10665
	Total all distances measured	36372
	Distances 0-150 nm	13035
	Total all distances measured	87029
	Distances 0-150 nm	21038
	Total all distances measured	19361
	Distances 0-150 nm	1426
	Total all distances measured	67832
	Distances 0-150 nm	18899
	Total all distances measured	42522
	Distances 0-150 nm	13560

**KO 14**	ER & actin	
	Ch2 & Ch3	Number of measurements
	Total all distances measured	29067
	Distances 0-150 nm	4150
	Total all distances measured	60865
	Distances 0-150 nm	37256
	Total all distances measured	2672
	Distances 0-150 nm	1288
	Total all distances measured	88084
	Distances 0-150 nm	59239
	Total all distances measured	4929
	Distances 0-150 nm	2382
	Total all distances measured	20869
	Distances 0-150 nm	11665
	Total all distances measured	48896
	Distances 0-150 nm	19998

**Summary:**	**ER & actin**			
	% Adjacent signal			
	WT NI	KO NI	WT 14	**KO 14**
	23,9442947	54,4789515	33,99639152	14,2773592
	23,7021621	62,7486673	27,76259274	61,2108765
	32,52821547	32,2777101	35,83800726	48,2035928
	6,618856504	63,5910489	24,17355134	67,2528496
	15,49671988	28,2734269	7,365322039	48,3262325
	20,21433275	57,7639752	27,86148131	55,8963055
	20,03470847	40,5687518	31,88937491	40,899051
	21,87563175	31,134058		
	9,222961303	36,6210695		
	11,36409802	21,9963671		
Mean	**18,50019809**	**42,9454026**	**30,25356651**	**48,0094667**
Std Deviation	7,86941073	15,3626784	4,39918613	17,2980651
Std error	2,488526175	4,85810547	1,662736067	6,53805407

T test:	ER & actin	P value
	WTNI vs KONI	0,00057569
	WT14Ivs KO14	0,00133378
	WTNI vs WT14	0,01133937
	KONI vs KO14	0,11007839

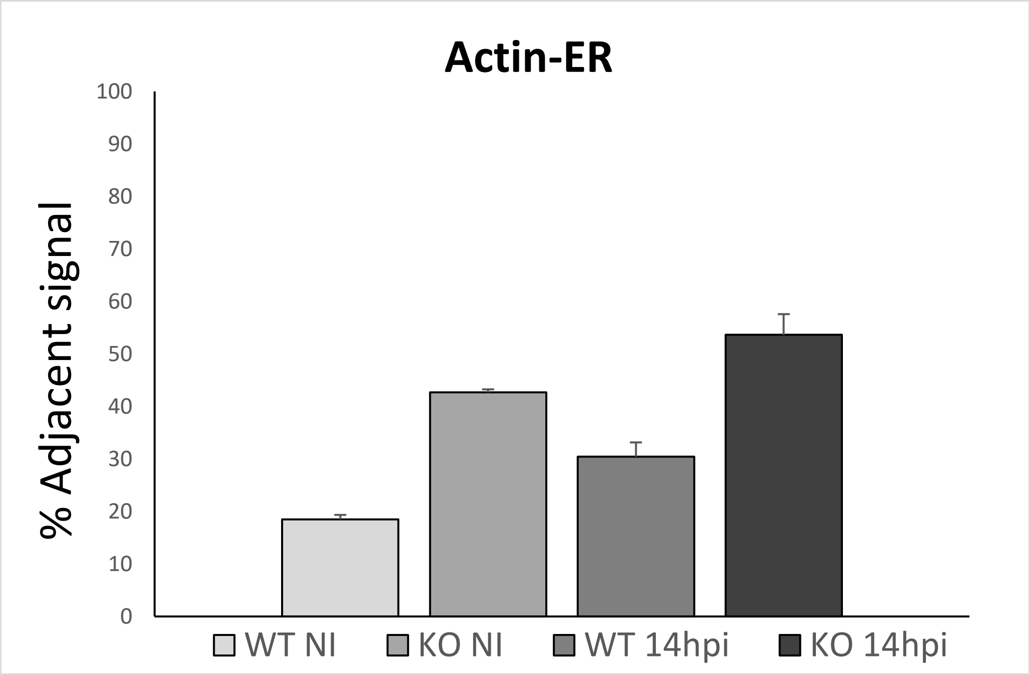

FIGURE 6E: Minimum distances

Between ER and muNS

ch1	R	ER
ch2	G	muNS
ch3	B	ANXA2

WT 14

ER & muNS	
Ch1 & Ch2	Number of measurements
Total all distances measured	50891
Distances 0-150 nm	23816
Total all distances measured	26771
Distances 0-150 nm	10082
Total all distances measured	12014
Distances 0-150 nm	5250
Total all distances measured	36657
Distances 0-150 nm	3949
Total all distances measured	38198
Distances 0-150 nm	5442
Total all distances measured	63804
Distances 0-150 nm	21846
Total all distances measured	86554
Distances 0-150 nm	13769
Total all distances measured	32109
Distances 0-150 nm	3524
Total all distances measured	56974
Distances 0-150 nm	20588

KO 14

ER & muNS	
Ch1 & Ch2	Number of measurements
Total all distances measured	21734
Distances 0-150 nm	11437
Total all distances measured	7735
Distances 0-150 nm	3880
Total all distances measured	128992
Distances 0-150 nm	53128
Total all distances measured	31121
Distances 0-150 nm	16629
Total all distances measured	9494
Distances 0-150 nm	5378
Total all distances measured	11113
Distances 0-150 nm	5983
Total all distances measured	1112
Distances 0-150 nm	553
Total all distances measured	4080
Distances 0-150 nm	2030
Total all distances measured	133765
Distances 0-150 nm	66935

**Summary:**	**ER & muNS**	
	% Adjacent signal	
	WT14	KO14
	46,7980586	52,6226189
	37,66015464	50,1616031
	43,69901781	41,1870504
	10,77284011	53,433373
	14,24681921	56,6463029
	34,23923265	53,8378476
	15,90798808	49,7302158
	10,97511601	49,754902
	36,13578123	50,0392479
Mean	**27,82611204**	**50,8236846**
Std Deviation	14,66279317	4,31988257
Std error	4,887597724	1,43996086

T test:	WT14 vs KO14	P value
	ER & muNS	0,00131613

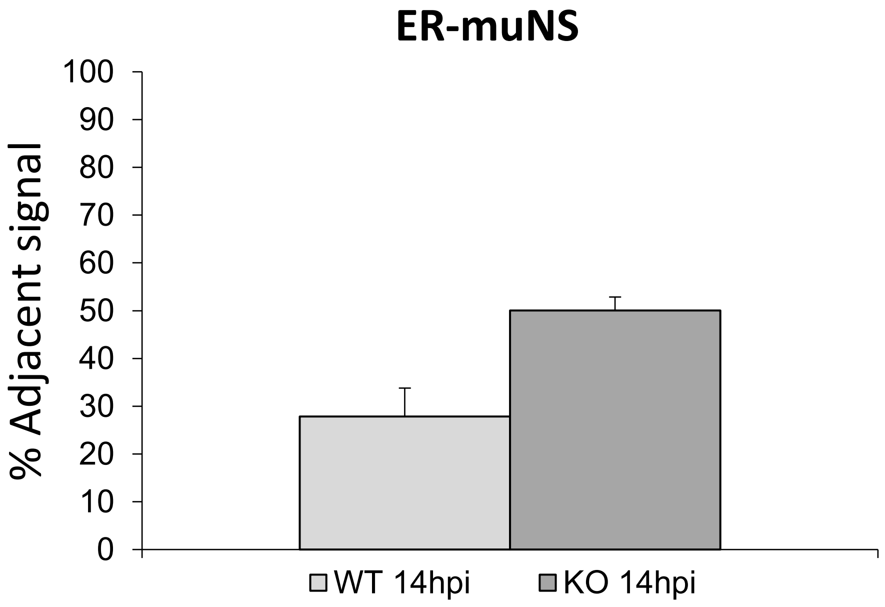

**FIGURE 6F:**	Minimum distances		
	Between ER and sigmaNS		
	ch1	R	ANXA2
	ch2	G	sigmaNS
	ch3	B	ER
**WT 14**	ER & sigmaNS		
	Ch3 & Ch1	Number of measurements	
	Total all distances measured	4912	
	Distances 0-150 nm	1289	
	Total all distances measured	7719	
	Distances 0-150 nm	2948	
	Total all distances measured	2260	
	Distances 0-150 nm	773	
	Total all distances measured	2820	
	Distances 0-150 nm	1527	
	Total all distances measured	152388	
	Distances 0-150 nm	30301	
	Total all distances measured	44300	
	Distances 0-150 nm	12984	
	Total all distances measured	2663	
	Distances 0-150 nm	958	
	Total all distances measured	7672	
	Distances 0-150 nm	2189	
	Total all distances measured	28790	
	Distances 0-150 nm	4565	
	Total all distances measured	29637	
	Distances 0-150 nm	14734	
**KO 14**			
	Total all distances measured	12950	
	Distances 0-150 nm	5080	
	Total all distances measured	154454	
	Distances 0-150 nm	11238	
	Total all distances measured	542	
	Distances 0-150 nm	368	
	Total all distances measured	122	
	Distances 0-150 nm	81	
	Total all distances measured	1907	
	Distances 0-150 nm	1046	
	Total all distances measured	317	
	Distances 0-150 nm	145	
	Total all distances measured	1365	
	Distances 0-150 nm	903	
	Total all distances measured	15473	
	Distances 0-150 nm	8612	
	Total all distances measured	1067	
	Distances 0-150 nm	725	
	Total all distances measured	4073	
	Distances 0-150 nm	2780	

Summary:	**ER & sigmaNS**	
	% Adjacent signal	
	WT14	KO14
	26,24185668	39,2277992
	38,19147558	7,27595271
	34,20353982	67,896679
	54,14893617	66,3934426
	19,88411161	54,8505506
	29,30925508	45,7413249
	35,97446489	66,1538462
	28,53232534	55,6582434
	15,85620007	67,9475164
	49,71488342	68,254358
Mean	**33,20570487**	**59,1248623**
Std Deviation	12,05855931	19,3366331
Std error	3,81325127	6,11478029

T test:	WT14 vs KO14	P value
	ER & sigmaNS	9,4911E-05

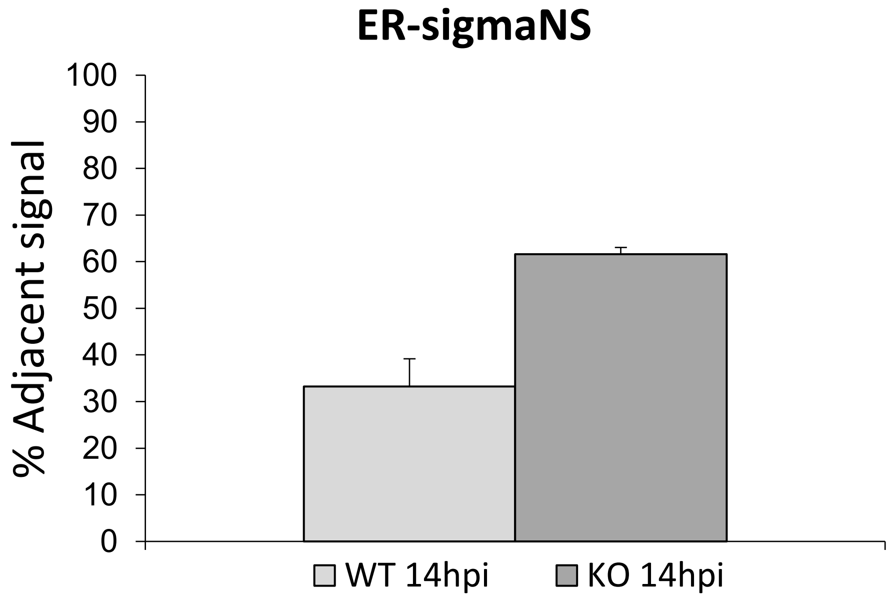

**FIGURE 6G:**	Minimum distances	
	Between Actin and muNS	
ch1	R	muNS
ch2	G	ANXA2
ch3	B	Act
**WT 14**	ACT & muNS	
	Ch3 & Ch1	Number of measurements
	Total all distances measured	364
	Distances 0-150 nm	305
	Total all distances measured	10097
	Distances 0-150 nm	2362
	Total all distances measured	14248
	Distances 0-150 nm	3566
	Total all distances measured	7800
	Distances 0-150 nm	1222
	Total all distances measured	21510
	Distances 0-150 nm	4737
	Total all distances measured	1472
	Distances 0-150 nm	331
	Total all distances measured	4867
	Distances 0-150 nm	1220
	Total all distances measured	34457
	Distances 0-150 nm	9159
	Total all distances measured	4745
	Distances 0-150 nm	967
	Total all distances measured	30363
	Distances 0-150 nm	4982
**KO 14**	ACT & muNS	
	Ch3 & Ch1	Number of measurements
	Total all distances measured	7230
	Distances 0-150 nm	1247
	Total all distances measured	4235
	Distances 0-150 nm	1564
	Total all distances measured	11776
	Distances 0-150 nm	3554
	Total all distances measured	14266
	Distances 0-150 nm	4319
	Total all distances measured	6580
	Distances 0-150 nm	2149
	Total all distances measured	1225
	Distances 0-150 nm	286
	Total all distances measured	3499
	Distances 0-150 nm	1099
	Total all distances measured	4571
	Distances 0-150 nm	1662
	Total all distances measured	4197
	Distances 0-150 nm	1245
	Total all distances measured	188
	Distances 0-150 nm	61

**Summary:**	**ACT & muNS**	
	% Adjacent signal	
	WT14	KO14
	83,79120879	17,24757953
	23,39308706	36,93034238
	25,02807412	30,18002717
	15,66666667	30,2747792
	22,0223152	32,65957447
	22,48641304	23,34693878
	25,06677625	31,40897399
	26,58095597	36,35965872
	20,37934668	29,66404575
	16,40812831	32,44680851
Mean	**22,57795437**	**30,05187285**
Std Deviation	19,89962044	5,874631362
Std error	6,292812515	1,857721552

T test:	WT14 vs KO14	P value
	ACT & muNS	8,76367E-05

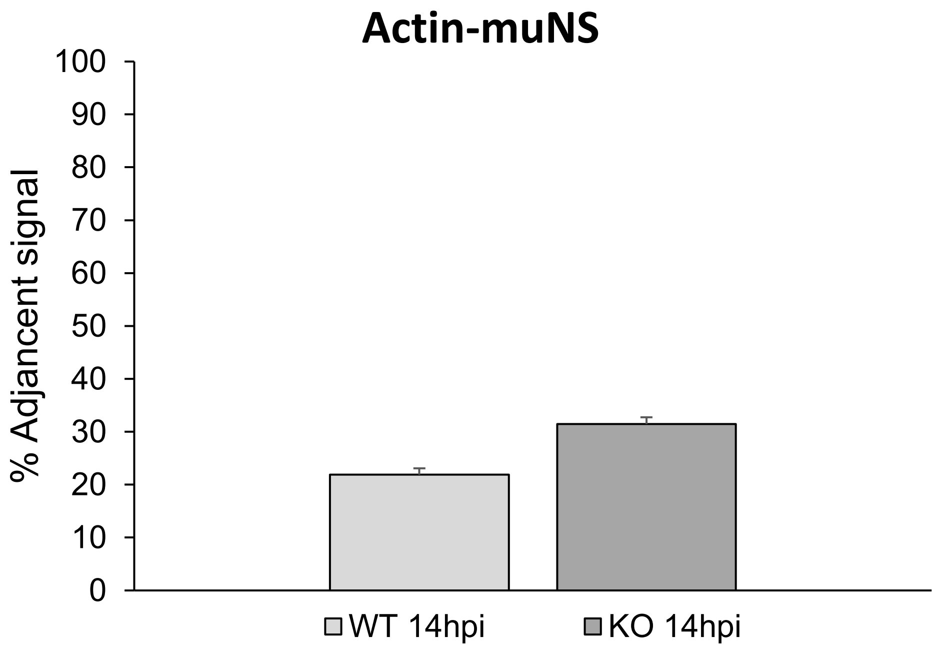

**FIGURE 6H:**	Minimum distances		
	Between Actin and sigmaNS		
	ch1	R	ANXA2
	ch2	G	sigmaNS
	ch3	B	Act
**WT 14**	ACT & sigmaNS		
	Ch3 & Ch2	Number of measurements	
	Total all distances measured	20456	
	Distances 0-150 nm	2820	
	Total all distances measured	18371	
	Distances 0-150 nm	2567	
	Total all distances measured	17531	
	Distances 0-150 nm	3472	
	Total all distances measured	25798	
	Distances 0-150 nm	3146	
	Total all distances measured	12009	
	Distances 0-150 nm	1653	
	Total all distances measured	84634	
	Distances 0-150 nm	8945	
	Total all distances measured	35489	
	Distances 0-150 nm	4471	
	Total all distances measured	23055	
	Distances 0-150 nm	2999	
	Total all distances measured	29294	
	Distances 0-150 nm	5123	
	Total all distances measured	43381	
	Distances 0-150 nm	5886	
**KO 14**	ACT & sigmaNS		
	Ch3 & Ch2	Number of measurements	
	Total all distances measured	44060	
	Distances 0-150 nm	5282	
	Total all distances measured	18692	
	Distances 0-150 nm	2229	
	Total all distances measured	9047	
	Distances 0-150 nm	609	
	Total all distances measured	16377	
	Distances 0-150 nm	1544	
	Total all distances measured	41466	
	Distances 0-150 nm	3385	
	Total all distances measured	957	
	Distances 0-150 nm	101	
	Total all distances measured	27405	
	Distances 0-150 nm	2751	
	Total all distances measured	10666	
	Distances 0-150 nm	1235	
	Total all distances measured	25324	
	Distances 0-150 nm	2999	
	Total all distances measured	40495	
	Distances 0-150 nm	3518	

**Summary:**	**ACT & sigmaNS**	
	% Adjacent signal	
	WT14	KO14
	13,78568635	11,9881979
	13,97310979	11,9248877
	19,804917	6,73151321
	12,19474378	9,42785614
	13,76467649	8,16331452
	10,56903845	10,553814
	12,59826989	10,0383142
	13,00802429	11,5788487
	17,48822284	11,8425209
	13,56815196	8,68749228
Mean	**14,13185432**	**10,093676**
Std Deviation	2,668241518	1,82186231
Std error	0,843772054	0,57612345

T test:	WT14 vs KO14	P value
	ACT & sigmaNS	0,00129564

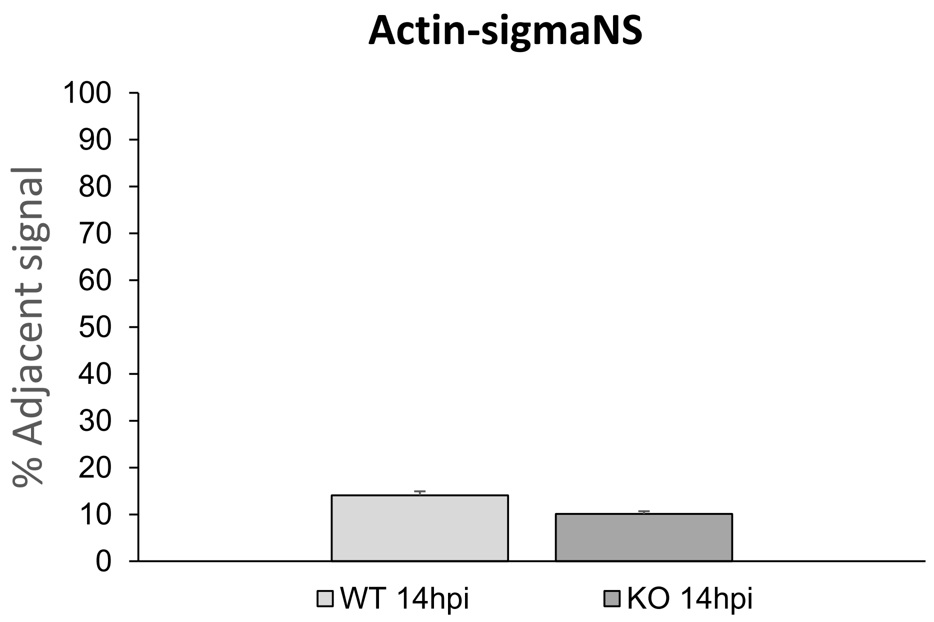

FIGURE S3: % Infected cell in Early times postinfection
Counting infected cells with large/small VFs

R1

Infected cells	WT 7hpi	KO 7hpi	WT 8hpi	KO 8hpi	WT 9hpi	KO 9hpi	WT 10hpi	KO 10hpi
Small VFs (< 1 µm)	6	24	33	44	64	89	99	222
Large VFs (≥ 1µm)	0	0	0	0	0	0	31	48
Total	6	24	33	44	64	89	130	270
Total cells	2512	2435	4947	2999	2891	2396	4673	3172
% Cells with Small VFs (< 1 µm)	0,24	0,99	0,67	1,47	2,21	3,71	2,13	6,99
% Cells with Large VFs (≥ 1µm)	0,00	0,00	0,00	0,00	0,00	0,00	0,65	1,52
% Total Infected cells	0,24	0,99	0,67	1,47	2,21	3,71	2,78	8,51

R2

Infected cells	WT 7hpi	KO 7hpi	WT 8hpi	KO 8hpi	WT 9hpi	KO 9hpi	WT 10hpi	KO 10hpi
Small VFs (< 1 µm)	6	10	12	43	39	101	73	109
Large VFs (≥ 1µm)	0	0	0	0	0	0	22	22
Total	6	10	12	43	39	101	95	131
Total cells	2355	932	1844	1366	1831	1071	1582	922
% Cells with Small VFs (< 1 µm)	0,25	1,07	0,43	2,12	1,26	6,82	3,16	6,94
% Cells with Large VFs (≥ 1µm)	0,00	0,00	0,22	1,03	0,87	2,62	2,84	7,27
% Total Infected cells	0,25	1,07	0,65	3,15	2,13	9,43	6,00	14,21

R3

Infected cells	WT 7hpi	KO 7hpi	WT 8hpi	KO 8hpi	WT 9hpi	KO 9hpi	WT 10hpi	KO 10hpi
Small VFs (< 1 µm)	3	11	15	41	33	70	44	70
Large VFs (≥ 1µm)	0	0	0	0	0	0	9	12
Total	3	11	15	41	33	70	53	82
Total cells	1659	765	1972	1137	2289	865	1268	728
% Cells with Small VFs (< 1 µm)	0,18	1,44	0,66	2,55	1,05	5,09	2,05	5,77
% Cells with Large VFs (≥ 1µm)	0,00	0,00	0,10	1,06	0,39	3,01	2,13	5,50
% Total Infected cells	0,18	1,44	0,76	3,61	1,44	8,09	4,18	11,27

Summary

	WT 7hpi	KO 7hpi	WT 8hpi	KO 8hpi	WT 9hpi	KO 9hpi	WT 10hpi	KO 10hpi
Mean % Cells with Small VFs (< 1 µm)	0,224819	1,1656	0,692799	2,740712	1,928554	7,0803	3,398293	9,487215
Mean % Cells with Large VFs (≥ 1µm)	0	0	0	0	0	0	0,923402	1,843058
Mean % Total Infected cells	**0,224819**	**1,1656**	**0,692799**	**2,740712**	**1,928554**	**7,0803**	**4,321695**	**11,33027**
Std deviation	0,038881	0,2401	0,059329	1,126381	0,423853	2,99096	1,616276	2,850925
Std error	0,022448	0,13862	0,034254	0,650317	0,244712	1,72683	0,933157	1,645982

	% infected cel	WT10	KO10
R1	Small VFs	76,47	82,09
	Large VFs	23,53	17,91
R2	Small VFs	76,67	83,55
	Lage VFs	23,33	16,45
R3	Small VFs	82,76	85,56
	Large VFs	17,24	14,44
Mean	Small VFs	78,63333333	83,73333333
	Large VFs	21,36666667	16,26666667
	**% cells with**		
Mean	Small VFs	3,398293217	9,487215406
	Large VFs	0,923402269	1,84305777

	% infected cel	WT10	KO10
R1	Large VFs	23,53	17,91
R2		23,33	16,45
R3		17,24	14,44

		p value
T Student:	Large VFs<br>WT vs KO<br>10 hpi	0,116027389

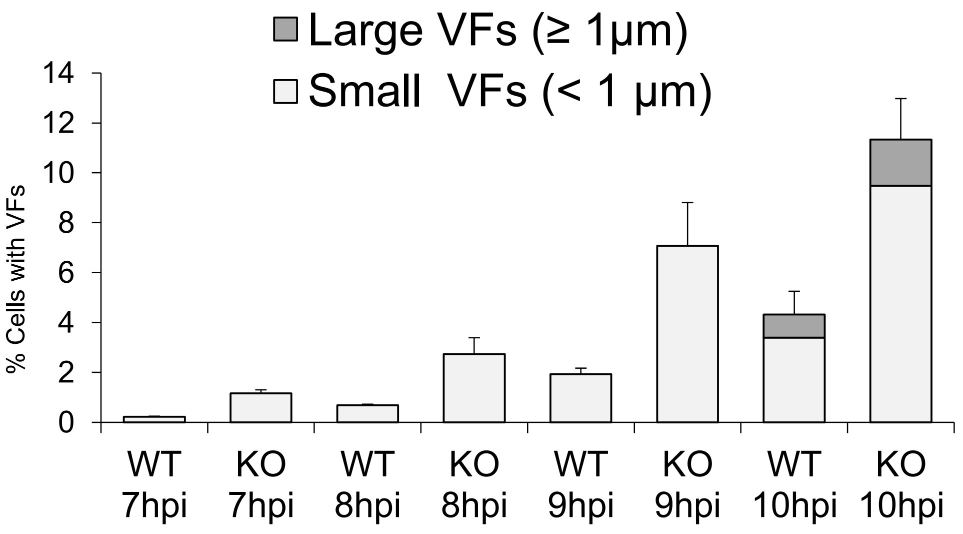

FIGURE S4: JaCoP DATA

WT1	Pearson	M1	M2	M1	M2	WT2	Pearson	M1	M2	M1	M2
1	0,855	1,000	1,000	0,72	0,825	1	0,888	1	0,963	0,843	0,95
2	0,86	1,000	1,000	0,85	0,908	2	0,89	1	0,944	0,943	0,91
3	0,831	1,000	1,000	0,74	0,94	3	0,885	1	0,95	0,963	0,86
4	0,904	1,000	1,000	0,91	0,976	4	0,862	1	0,939	0,908	0,88
5	0,932	0,999	0,999	0,88	0,973	5	0,9	1	0,962	0,939	0,92
6	0,846	1,000	1,000	0,89	0,838	6	0,866	1	0,926	0,883	0,86
7	0,864	1,000	1,000	0,88	0,937	7	0,86	1	0,836	0,96	0,7
8	0,817	1,000	1,000	0,84	0,974	8	0,896	1	0,73	0,991	0,79
9	0,881	1,000	1,000	0,87	0,94	9	0,87	1	0,617	0,979	0,71
10	0,851	1,000	1,000	0,92	0,946	10	0,831	1	0,712	0,902	0,71
11	0,882	1,000	1,000	0,87	0,949	11	0,898	1	0,959	0,807	0,97
12	0,862	1,000	1,000	0,86	0,974	12	0,853	1	1	0,734	0,96
mean	**0,865**	**1,000**	**1,000**	**0,852**	**0,932**	mean	**0,875**	**1,000**	**0,878**	**0,904**	**0,851**

ANXA2KO	Pearson	M1	M2	M1	M2	ANXA2KO2	Pearson	M1	M2	M1	M2
1	0,874	0,999	0,947	0,789	0,853	1	0,876	1,000	0,815	0,858	0,560
2	0,874	1,000	0,961	0,596	0,890	2	0,864	1,000	0,819	0,829	0,588
3	0,771	0,999	0,947	0,672	0,680	3	0,845	1,000	0,804	0,749	0,566
4	0,815	1,000	0,964	0,771	0,797	4	0,811	1,000	0,977	0,758	0,754
5	0,848	1,000	0,961	0,441	0,764	5	0,848	1,000	0,822	0,733	0,699
6	0,786	0,999	0,936	0,663	0,769	6	0,865	0,999	0,909	0,747	0,643
7	0,754	0,999	0,962	0,776	0,462	7	0,828	1,000	0,937	0,729	0,830
8	0,832	1,000	0,983	0,644	0,845	8	0,742	1,000	0,845	0,732	0,544
9	0,876	1,000	0,967	0,451	0,557	9	0,835	1,000	0,887	0,651	0,761
10	0,773	1,000	0,954	0,277	0,557	10	0,763	1,000	0,875	0,549	0,622
11	0,795	1,000	0,983	0,640	0,636	11	0,736	1,000	0,856	0,602	0,508
12	0,697	0,999	0,937	0,744	0,547	12	0,777	1,000	0,871	0,617	0,731
mean	**0,808**	**1,000**	**0,959**	**0,622**	**0,696**	mean	**0,816**	**1,000**	**0,868**	**0,713**	**0,651**

WT3	Pearson	M1	M2	M1	M2	ANXA2KO3	Pearson	M1	M2	M1	M2
1	0,898	1	1,000	0,94	0,836	1	0,797	1	0,952	0,756	0,74
2	0,911	1	1,000	0,89	0,916	2	0,765	1	0,937	0,585	0,75
3	0,813	1	1,000	0,87	0,89	3	0,784	1	0,959	0,551	0,47
4	0,87	1	1,000	0,88	0,904	4	0,771	1	0,958	0,631	0,68
5	0,882	1	1,000	0,9	0,935	5	0,817	1	0,968	0,685	0,74
6	0,746	1	1,000	0,75	0,919	6	0,806	1	0,995	0,714	0,66
7	0,768	1	1,000	0,8	0,918	7	0,729	1	0,999	0,633	0,67
8	0,87	1	1,000	0,85	0,911	8	0,826	1	0,995	0,672	0,75
9	0,89	1	1,000	0,86	0,861	9	0,702	1	0,996	0,886	0,46
10	0,881	1	1,000	0,87	0,886	10	0,796	1	0,999	0,789	0,6
11	0,922	1	1,000	0,88	0,946	11	0,821	1	0,897	0,686	0,71
12	0,853	1	1,000	0,73	0,963	12	0,82	1	0,963	0,812	0,82
mean	**0,859**	**1,000**	**1,000**	**0,853**	**0,907**	mean	**0,786**	**1,000**	**0,968**	**0,700**	**0,669**

Pearson		Mander	1	Mander's	2
WT	KO	WT	KO	WT	KO
0,855	0,874	0,722	0,789	0,825	0,853
0,86	0,874	0,852	0,596	0,908	0,890
0,831	0,771	0,742	0,672	0,94	0,680
0,904	0,815	0,909	0,771	0,976	0,797
0,932	0,848	0,876	0,441	0,973	0,764
0,846	0,786	0,885	0,663	0,838	0,769
0,864	0,754	0,879	0,776	0,937	0,462
0,817	0,832	0,839	0,644	0,974	0,845
0,881	0,876	0,87	0,451	0,94	0,557
0,851	0,773	0,919	0,277	0,946	0,557
0,882	0,795	0,873	0,640	0,949	0,636
0,862	0,697	0,855	0,744	0,974	0,547
0,888	0,876	0,843	0,858	0,953	0,560
0,89	0,864	0,943	0,829	0,905	0,588
0,885	0,845	0,963	0,749	0,864	0,566
0,862	0,811	0,908	0,758	0,875	0,754
0,9	0,848	0,939	0,733	0,916	0,699
0,866	0,865	0,883	0,747	0,857	0,643
0,86	0,828	0,96	0,729	0,698	0,830
0,896	0,742	0,991	0,732	0,794	0,544
0,87	0,835	0,979	0,651	0,714	0,761
0,831	0,763	0,902	0,549	0,712	0,622
0,898	0,736	0,807	0,602	0,966	0,508
0,853	0,777	0,734	0,617	0,963	0,731
0,898	0,797	0,944	0,76	0,836	0,735
0,911	0,765	0,894	0,59	0,916	0,746
0,813	0,784	0,871	0,55	0,89	0,465
0,87	0,771	0,882	0,63	0,904	0,676
0,882	0,817	0,904	0,69	0,935	0,735
0,746	0,806	0,753	0,71	0,919	0,66
0,768	0,729	0,802	0,63	0,918	0,672
0,87	0,826	0,85	0,67	0,911	0,75
0,89	0,702	0,858	0,89	0,861	0,462
0,881	0,796	0,866	0,79	0,886	0,598
0,922	0,821	0,881	0,69	0,946	0,713
0,853	0,82	0,734	0,81	0,963	0,821

Summary

WT	Pearson	M1	M2	M1	M2
WT1	0,865	1,000	1,000	0,852	0,932
WT2	0,875	1,000	0,878	0,904	0,851
WT3	0,859	1,000	1,000	0,853	0,907
Mean	**0,866**	**1,000**	**0,959**	**0,870**	**0,897**
Std deviation	0,008	0,000	0,070	0,030	0,041
Std error	0,005	0,000	0,041	0,017	0,024

KO	Pearson's	Mander's 1	Mander's 2	M1	M2
KO1	0,808	1,000	0,959	0,622	0,696
KO2	0,816	1,000	0,868	0,713	0,651
KO3	0,786	1,000	0,968	0,700	0,669
Mean	**0,803**	**1,000**	**0,932**	**0,678**	**0,672**
std deviation	0,015	0,000	0,055	0,049	0,023
std error	0,009	0,000	0,032	0,028	0,013

T test Pearson's Wt vs KO		T test Mander's 1 WT vs KO		T test Mander's 2 WT vs KO	
p value:	0,00780549	p value:	0,007924471	p value:	0,003116342

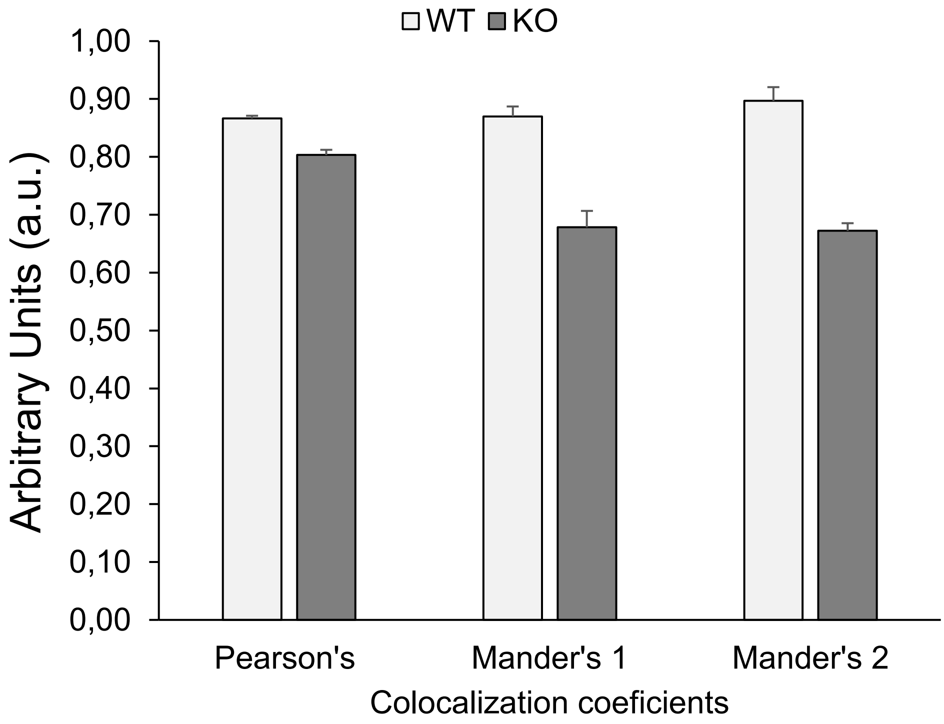

WT	Pearson	Mander's 1	Mander's 2
c1	0,865	0,852	0,932
c2	0,875	0,904	0,851
c3	0,859	0,853	0,907
Mean	**0,866**	**0,870**	**0,897**
Std deviation	0,008	0,030	0,041
Std error	0,005	0,017	0,024
KO	Pearson's	Mander's 1	Mander's 2
k1	0,808	0,622	0,696
k2	0,816	0,713	0,651
k3	0,786	0,700	0,669
Mean	**0,803**	**0,678**	**0,672**
Std deviation	0,015	0,049	0,023
Std error	0,009	0,028	0,013

**WT**	**Pearson's**	**Mander's 1**	**Mander's 2**
WT1	0.865	0.851	0.931
WT2	0.874	0.904	0.851
WT3	0.858	0.853	0.907
Average	0.866	0.869	0.896
**KO**	**Pearson's**	**Mander's 1**	**Mander's 2**
KO1	0.807	0.622	0.696
KO2	0.815	0.712	0.650
KO3	0.786	0.7	0.669
Average	0.803	0.678	0.672

FIGURE S7B: % Cells with the main ER morphologies in transfected cells

Cell number with these phenotypes

		Stretched ER	<6nm Small ER collapses	Fragmented ER	> 6nm Large ER collapses	total cells
WT	Total	15	6	4	2	16
sigmaNS	R1	5	3	3	0	5
transfection	R2	4	1	0	0	5
	R3	6	2	1	2	6
KO	Total	1	10	1	0	12
sigmaNS	R1	0	2	0	0	3
tranfection	R2	1	5	0	0	6
	R3	0	3	1	0	3
WT	Total	1	10	12	2	20
muNS	R1	1	0	7	0	9
transfection	R2	0	4	4	1	4
	R3	0	6	7	1	7
KO	Total	0	8	10	16	18
muNS	R1	0	1	3	6	6
transfection	R2	0	2	2	4	5
	R3	0	5	5	6	7
WT	Total	6	9	8	3	13
muNS+sigmaNS	R1	1	3	2	1	5
transfection	R2	2	3	2	1	4
	R3	3	3	4	1	4
KO	Total	1	12	6	8	14
muNS+sigmaNS	R1	1	3	3	2	5
transfection	R2	0	4	2	3	4
	R3	0	5	1	3	5

% Cells with these phenotypes

	Stretched ER	<6nm Small ER collapses	Fragmented ER	> 6nm Large ER collapses	total cells
σNS-transfected WT cells					16
R1	100,00	60,00	60,00	0,00	5
R2	80,00	20,00	0,00	0,00	5
R3	100,00	33,33	16,67	33,33	6
Mean	93,33	37,78	25,56	11,11	
Std deviation	11,5	20,4	31,0	19,2	
Std error	6,67	11,76	17,88	11,11	
σNS-tranfected KO cells					12
R1	11,11	0,00	77,78	0,00	3
R2	0,00	100,00	100,00	25,00	6
R3	0,00	85,71	100,00	14,29	3
Mean	3,70	61,90	92,59	13,10	
Std deviation	6,4	54,1	12,8	12,5	
Std error	3,70	31,23	7,41	7,24	
μNS-transfected WT cells					20
R1	20,00	60,00	40,00	20,00	9
R2	50,00	75,00	50,00	25,00	4
R3	75,00	75,00	100,00	25,00	7
Mean	48,33	70,00	63,33	23,33	
Std deviation	27,5	8,7	32,1	2,9	
Std error	15,90	5,00	18,56	1,67	
mNS-transfeced KO cells					18
R1	2000,00	1200,00	1200,00	0,00	6
R2	1600,00	400,00	0,00	0,00	5
R3	1666,67	555,56	277,78	555,56	7
Mean	1755,56	718,52	492,59	185,19	
Std deviation	214,3	424,2	628,2	320,8	
Std error	123,73	244,89	362,68	185,19	
mNS+ σNS-transfected WT cells					13
R1	370,37	0,00	2592,59	0,00	5
R2	0,00	1666,67	1666,67	416,67	4
R3	0,00	2857,14	3333,33	476,19	4
Mean	123,46	1507,94	2530,86	297,62	
Std deviation	213,8	1435,2	835,0	259,5	
Std error	123,46	828,60	482,11	149,80	
mNS+ σNS-transfected KO cells					14
R1	222,22	666,67	444,44	222,22	5
R2	1250,00	1875,00	1250,00	625,00	4
R3	1071,43	1071,43	1428,57	357,14	5
Mean	847,88	1204,37	1041,01	401,46	
Std deviation	549,1	615,0	524,3	205,0	
Std error	317,05	355,09	302,70	118,36	

48,33	70,00	63,33	23,33

	ER MORPHOLOGY			
	Stretched ER	Small ER collapses	Fragmented ER	Large ER collapses
σNS-transfected WT cells	15 / 93.33%	6 / 37.77%	4 /25.55%	2 / 11.11%
σNS-transfected KO cells	1 / 5.55%	10 /83.33%	1 /11.11%	0 /0%
µNS-transfected WT cells	1 / 3.70%	10 / 61.90%	12 / 92.59%	2 / 13.09%
µNS-transfected KO cells	0 /0%	8 / 42.69%	10 / 53.80%	16 / 88.57%
µNS + σNS transfected WT cells	6 / 48.33%	9 / 70%	8 / 63.33%	3 / 23.33%
µNS + σNS transfected KO cells	1 / 6.67%	12 / 86.67%	6 / 43.33%	8 / 58,33%

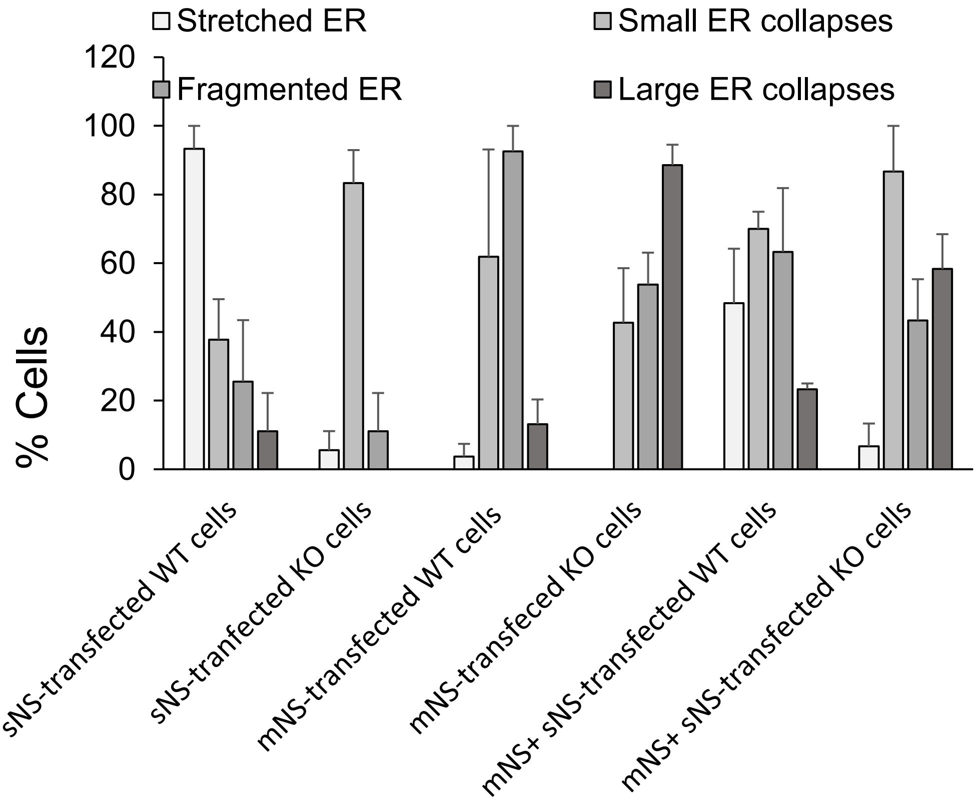

FIGURE S8C: Minimum distances (Biological test)

	Between giantin (Golgi complex) and muNS	
Ch1	R	muNS
Ch2	G	Giantin
Ch3	B	Act
**WT 14hpi**	Ch2 & Ch1	Number of measurements
	Total all distances measured	5086
	Distances 0-150 nm	11
	Total all distances measured	3202
	Distances 0-150 nm	34
	Total all distances measured	3734
	Distances 0-150 nm	27
	Total all distances measured	6131
	Distances 0-150 nm	76
	Total all distances measured	3797
	Distances 0-150 nm	13
	Total all distances measured	3771
	Distances 0-150 nm	13
	Total all distances measured	7157
	Distances 0-150 nm	8
	Total all distances measured	1592
	Distances 0-150 nm	8
	Total all distances measured	1252
	Distances 0-150 nm	0
	Total all distances measured	6463
	Distances 0-150 nm	68

**KO 14hpi**	Ch2 & Ch1	Number of measurements
	Total all distances measured	2692
	Distances 0-150 nm	3
	Total all distances measured	1484
	Distances 0-150 nm	15
	Total all distances measured	3628
	Distances 0-150 nm	19
	Total all distances measured	6223
	Distances 0-150 nm	29
	Total all distances measured	794
	Distances 0-150 nm	5
	Total all distances measured	2841
	Distances 0-150 nm	0
	Total all distances measured	1646
	Distances 0-150 nm	0
	Total all distances measured	3738
	Distances 0-150 nm	0
	Total all distances measured	3830
	Distances 0-150 nm	1
	Total all distances measured	3558
	Distances 0-150 nm	1

Summary:

	% Adjacent signal	
	WT14	KO14
	0,216279984	0,11144131
	29109,09091	49466,6667
	1,061836352	1,01078167
	10982,35294	24186,6667
	0,723085163	0,52370452
	22707,40741	32752,6316
	1,239602023	0,46601318
	4996,052632	2737,93103
	0,34237556	0,62972292
	29007,69231	56820
Mean	**9680,617938**	**16596,6638**
Std Deviation	12529,25786	22573,8222
Std error	3962,099223	7138,46935

T test:	WT14 vs KO14	P value
	Golgi & muNS	0,39957206

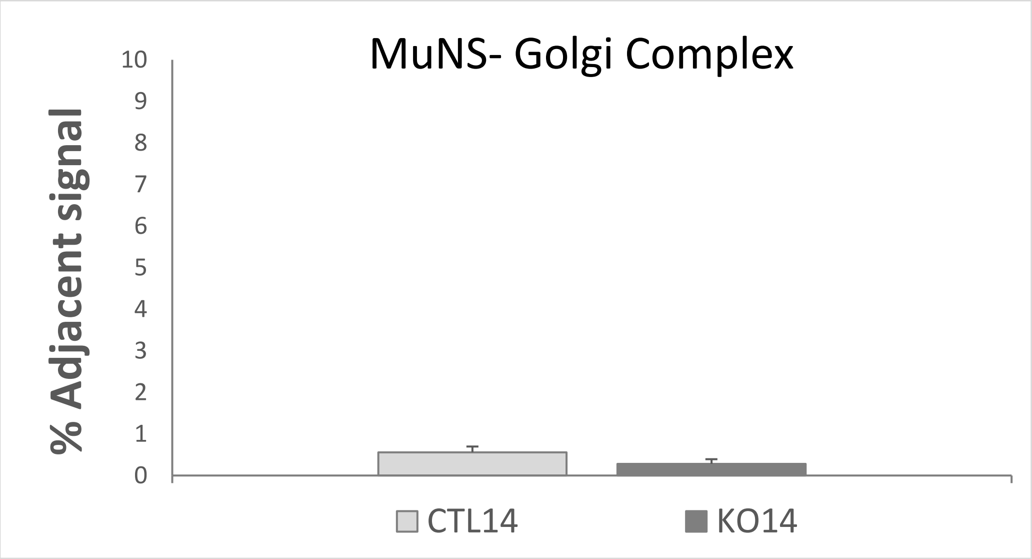

FIGURE S8D: Minimum distances (Biological test)
Between gigantin (Golgi complex) and nucleus

Ch1	R	muNS
Ch2	G	Giantin
Ch3	B	DAPI

**WT NI**	Ch3 & Ch2	Number of measurements
1	Total all distances measured	4895
	Distances 0-150 nm	25
2	Total all distances measured	1125
	Distances 0-150 nm	9
3	Total all distances measured	1857
	Distances 0-150 nm	14
4	Total all distances measured	2167
	Distances 0-150 nm	15
5	Total all distances measured	1880
	Distances 0-150 nm	8
6	Total all distances measured	1469
	Distances 0-150 nm	9
7	Total all distances measured	1776
	Distances 0-150 nm	17
8	Total all distances measured	2520
	Distances 0-150 nm	32
9	Total all distances measured	1560
	Distances 0-150 nm	56
10	Total all distances measured	5296
	Distances 0-150 nm	145

**WT 14hpi**	Ch3 & Ch2	Number of measurements
1	Total all distances measured	2298
	Distances 0-150 nm	32
2	Total all distances measured	551
	Distances 0-150 nm	14
3	Total all distances measured	866
	Distances 0-150 nm	7
4	Total all distances measured	1660
	Distances 0-150 nm	22
5	Total all distances measured	1583
	Distances 0-150 nm	10
6	Total all distances measured	1782
	Distances 0-150 nm	10
7	Total all distances measured	2667
	Distances 0-150 nm	11
8	Total all distances measured	534
	Distances 0-150 nm	3
9	Total all distances measured	1190
	Distances 0-150 nm	5
10	Total all distances measured	2230
	Distances 0-150 nm	57

KO NI		Ch3 & Ch2	Number of measurements
	1	Total all distances measured	1285
		Distances 0-150 nm	86
	2	Total all distances measured	409
		Distances 0-150 nm	1
	3	Total all distances measured	270
		Distances 0-150 nm	0
	4	Total all distances measured	1757
		Distances 0-150 nm	3
	5	Total all distances measured	883
		Distances 0-150 nm	1
	6	Total all distances measured	1903
		Distances 0-150 nm	2
	7	Total all distances measured	1437
		Distances 0-150 nm	4
	8	Total all distances measured	2320
		Distances 0-150 nm	3
	9	Total all distances measured	1278
		Distances 0-150 nm	30
	10	Total all distances measured	2711
		Distances 0-150 nm	0

KO 14hpi		Ch3 & Ch2	Number of measurements
	1	Total all distances measured	2057
		Distances 0-150 nm	9
	2	Total all distances measured	1402
		Distances 0-150 nm	6
	3	Total all distances measured	563
		Distances 0-150 nm	16
	4	Total all distances measured	278
		Distances 0-150 nm	3
	5	Total all distances measured	526
		Distances 0-150 nm	6
	6	Total all distances measured	1578
		Distances 0-150 nm	4
	7	Total all distances measured	1699
		Distances 0-150 nm	2
	8	Total all distances measured	1143
		Distances 0-150 nm	7
	9	Total all distances measured	980
		Distances 0-150 nm	5
	10	Total all distances measured	1086
		Distances 0-150 nm	0

Summary:

	% Adjacent signal			
	WT NI	WT 14	KO NI	KO 14
	0,51072523	1,39251523	6,692607	0,43753038
	0,8	2,54083485	0,24449878	0,42796006
	0,753904146	0,80831409	0	2,84191829
	0,6922012	1,3253012	0,17074559	1,07913669
	0,425531915	0,63171194	0,11325028	1,14068441
	0,612661675	0,56116723	0,10509721	0,25348542
	0,957207207	0,41244844	0,27835769	0,1177163
	1,26984127	0,56179775	0,12931034	0,61242345
	3,58974359	0,42016807	2,34741784	0,51020408
	2,737915408	2,55605381	0	0
Mean	**1,234973164**	**1,12103126**	**1,00812847**	**0,74210591**
Std Deviation	1,062720885	0,82645668	2,11726075	0,82482658
Std error	0,375728572	0,26134855	0,66953664	0,26083307

T test:	Golgi & nucleus	P value
	WT14 vs KO14	0,31837522
	WT NI vs KO NI	0,76673497

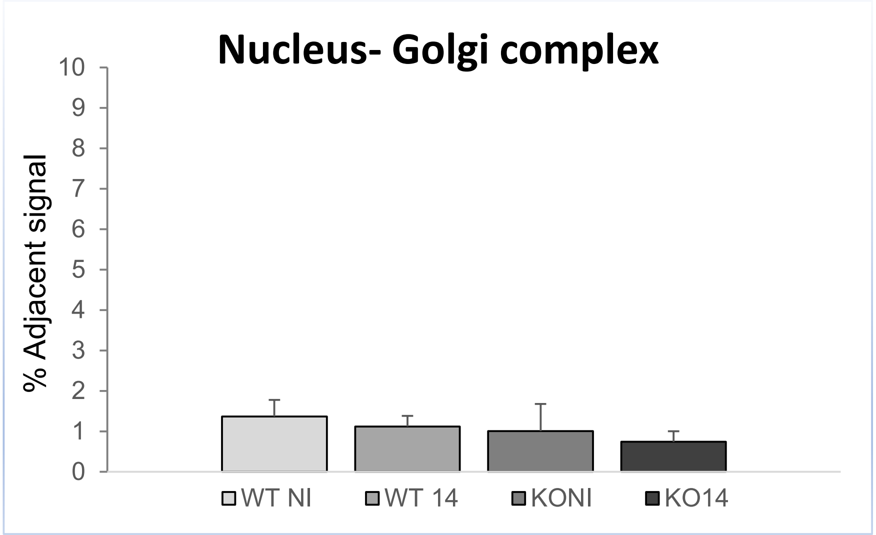

FIGURE S10A: Recomplemented cells (CTL /KO/KO+)
Counting = Infected cells/total cells

R1					R2				
14hpi		WT	KO	KOrecompl.	14hpi		WT	KO	KOrecompl.
	1	0,018519	0,04545	0,01886792		1	0,21875	0,392857143	0,14285714
	2	0,023256	0,05882	0,04545455		2	0,2258065	0,239130435	0,15384615
	3	0,027778	0,03175	0,04761905		3	0,2222222	0,266666667	0,09302326
	4	0,060606	0,05155	0,0212766		4	0,0961538	0,35	0,10869565
	5	0,047619	0,06481	0		5	0,1666667	0,20754717	0,1025641
	6	0,047619	0,02299	0,07272727		6	0,2631579	0,195121951	0,11904762
	7	0,057143	0,05	0		7	0,1538462	0,269230769	0,12
	8	0,019608	0,03175	0,0483871		8	0,2105263	0,282051282	0,08108108
	9	0,060606	0,03571	0,01538462		9	0,2321429	0,285714286	0,15384615
	10	0,054054	0,05357	0,02380952		10	0,2295082	0,304347826	0,11627907
Mean		0,041681	0,04464	0,02935266	Mean		0,2018781	0,279266753	0,11912402
**%**		**4,168071**	**4,46406**	**2,93526622**	**%**		**20,187806**	**27,92667528**	**11,9124023**
24hpi		WT	KO	KOrecompl.	24hpi		WT	KO	KOrecompl.
	1	0,116279	0,2069	0,08888889		1	0,34	0,590909091	0,37209302
	2	0,121951	0,07692	0,1		2	0,4318182	0,307692308	0,28947368
	3	0,162791	0,21053	0,04081633		3	0,3272727	0,322580645	0,26470588
	4	0,137931	0,21429	0,04347826		4	0,2727273	0,515151515	0,20454545
	5	0,194444	0,17647	0,07017544		5	0,5185185	0,516129032	0,23809524
	6	0,111111	0,15152	0,10714286		6	0,3589744	0,41025641	0,15384615
	7	0,1	0,22222	0,08510638		7	0,2982456	0,424242424	0,31111111
	8	0,157895	0,22857	0,08695652		8	0,3043478	0,5	0,23684211
	9	0,081081	0,16981	0,10869565		9	0,1836735	0,387096774	0,20833333
	10	0,269231	0,17647	0,05714286		10	0,3090909	0,4	0,09090909
Mean		0,145271	0,18337	0,07884032	Mean		0,3344669	0,43740582	0,23699551
**%**		**14,52714**	**18,3369**	**7,88403186**	**%**		**33,446689**	**43,740582**	**23,6995508**

R3

14hpi		WT	KO	KOrecompl.
	1	0,189189	0,28	0,10526316
	2	0,338983	0,27273	0,15517241
	3	0,212766	0,37931	0,12727273
	4	0,214286	0,31429	0,15686275
	5	0,163636	0,43478	0,2
	6	0,222222	0,3871	0,15686275
	7	0,278689	0,57692	0,17857143
	8	0,209302	0,29167	0,25
	9	0,212766	0,33333	0,125
	10	0,16129	0,35556	0,13513514
Mean		0,220313	0,36257	0,15901404
**%**		**22,0313**	**36,2568**	**15,9014035**

24hpi		WT	KO	KOrecompl.
	1	0,433962	0,42857	0,2
	2	0,40678	0,42424	0,18518519
	3	0,232143	0,38095	0,23684211
	4	0,319149	0,4	0,25581395
	5	0,275	0,42857	0,21621622
	6	0,372549	0,35714	0,34782609
	7	0,280702	0,30612	0,17073171
	8	0,342466	0,35593	0,34782609
	9	0,235294	0,28814	0,21428571
	10	0,280488	0,3617	0,19047619
Mean		0,317853	0,37314	0,23652032
**%**		**31,78532**	**37,3137**	**23,6520325**

**Summary**		R1	R2	R3			
		WT	WT	WT	Mean	Std deviation	Std error
	14hpi	4,1680708	20,18780605	22,0312963	15,462391	9,82450336	5,67217966
	24hpi	14,527142	33,44668878	31,7853217	26,586384	10,4765944	6,0486646
		KO	KO	KO			
	14hpi	4,4640556	27,92667528	36,2568135	22,8825148	16,4856765	9,51800975
	24hpi	18,33693	43,740582	37,3137289	33,1304135	13,2083869	7,62586574
		KO+	KO+	KO+			
	14hpi	2,9352662	11,91240231	15,9014035	10,2496907	6,64105689	3,83421598
	24hpi	7,8840319	23,69955077	23,6520325	18,4118717	9,1174077	5,26393779

			p value				p value
T student	14hpi	CTL vs KO	0,025093303	T student	14hpi	KO vs KO+	0,0001283
	24hpi	CTL vs KO	0,02467409		24hpi	KO vs KO+	4,7774E-06

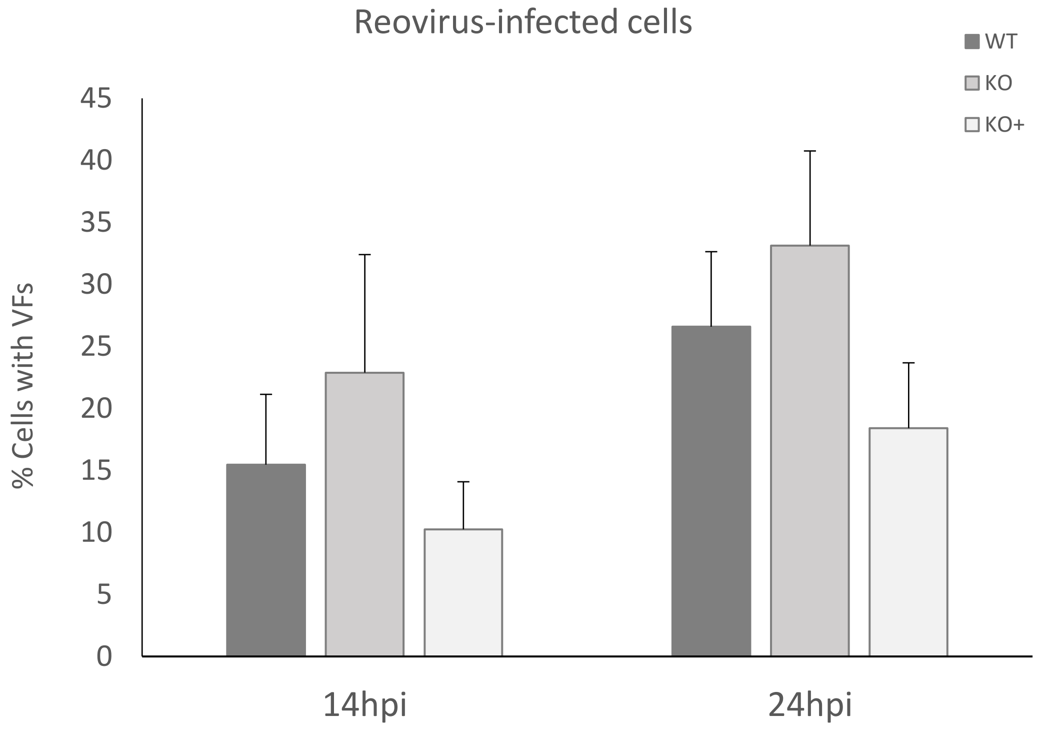

**All data**		14hpi WT	24hpi WT	14hpi KO	24hpi KO	14hpi KO+	24hpi KO+
	R1	0,0185185	0,11627907	0,04545455	0,20689655	0,01886792	0,08888889
		0,0232558	0,12195122	0,05882353	0,07692308	0,04545455	0,1
		0,0277778	0,162790698	0,03174603	0,21052632	0,04761905	0,04081633
		0,0606061	0,137931034	0,05154639	0,21428571	0,0212766	0,04347826
		0,047619	0,194444444	0,06481481	0,17647059	0	0,07017544
		0,047619	0,111111111	0,02298851	0,15151515	0,07272727	0,10714286
		0,0571429	0,1	0,05	0,22222222	0	0,08510638
		0,0196078	0,157894737	0,03174603	0,22857143	0,0483871	0,08695652
		0,0606061	0,081081081	0,03571429	0,16981132	0,01538462	0,10869565
		0,0540541	0,269230769	0,05357143	0,17647059	0,02380952	0,05714286
	R2	0,21875	0,34	0,39285714	0,59090909	0,14285714	0,37209302
		0,2258065	0,431818182	0,23913043	0,30769231	0,15384615	0,28947368
		0,2222222	0,327272727	0,26666667	0,32258065	0,09302326	0,26470588
		0,0961538	0,272727273	0,35	0,51515152	0,10869565	0,20454545
		0,1666667	0,518518519	0,20754717	0,51612903	0,1025641	0,23809524
		0,2631579	0,358974359	0,19512195	0,41025641	0,11904762	0,15384615
		0,1538462	0,298245614	0,26923077	0,42424242	0,12	0,31111111
		0,2105263	0,304347826	0,28205128	0,5	0,08108108	0,23684211
		0,2321429	0,183673469	0,28571429	0,38709677	0,15384615	0,20833333
		0,2295082	0,309090909	0,30434783	0,4	0,11627907	0,09090909
	R3	0,1891892	0,433962264	0,28	0,42857143	0,10526316	0,2
		0,3389831	0,406779661	0,27272727	0,42424242	0,15517241	0,18518519
		0,212766	0,232142857	0,37931034	0,38095238	0,12727273	0,23684211
		0,2142857	0,319148936	0,31428571	0,4	0,15686275	0,25581395
		0,1636364	0,275	0,43478261	0,42857143	0,2	0,21621622
		0,2222222	0,37254902	0,38709677	0,35714286	0,15686275	0,34782609
		0,2786885	0,280701754	0,57692308	0,30612245	0,17857143	0,17073171
		0,2093023	0,342465753	0,29166667	0,3559322	0,25	0,34782609
		0,212766	0,235294118	0,33333333	0,28813559	0,125	0,21428571
		0,1612903	0,280487805	0,35555556	0,36170213	0,13513514	0,19047619

FIGURE S10B: Recomplemented cells
WB µNS/Tomm22

R1					µNS/Tomm22
µNS	14hpi	MWT14	3.042.001.278.611	304,2001279	0,43461585
		MKOG14	1.871.752.648.360	187,1752648	0,20221765
		MKOGplus14	1.593.043.510.571	159,3043511	0,22248753
	24hpi	MWT24	5.225.222.857.963	522,5222858	2,57607806
		MKOg24	236.988.788.460	23,69887885	0,11986892
		MKOgplus24	9.247.293.924	0,924729392	0,00479206
Tomm22	14hpi	TWT14	6.999.287.523.421	699,9287523	
		TKOG14	9.256.129.031.917	925,6129032	
		TKOGplus14	7.160.147.427.022	716,0147427	
	24hpi	TWT24	2.028.363.555.807	202,8363556	
		TKO24	1.977.066.164.088	197,7066164	
		TKOplus24	1.929.711.417.065	192,9711417	
		b	0.0000000000		
R2					µNS/Tomm22
µNS	14hpi	MWT14	6.909.520.672.136	690,9520672	0,58860654
		MKOG14	6.504.790.272.969	650,4790273	0,57912447
		MKOGplus14	6.768.947.467.608	676,8947468	0,62765443
	24hpi	MWT24	1.960.569.680.696	196,0569681	1,22424666
		MKOG24	2.567.450.226.099	256,7450226	1,72715692
		MKOGplus24	1.815.336.986.982	181,5336987	1,30252285
Tomm22	14hpi	TWT14	11.738.776.554.825	1173,877655	
		TKO14	11.232.110.869.152	1123,211087	
		TKOplus14	10.784.513.169.471	1078,451317	
	24hpi	TWT24	16.014.498.907.653	160,1449891	
		TKOg24	14.865.182.136.516	148,6518214	
		TKOgplus24	13.937.083.575.055	139,3708358	
		b	0.0000000000		
R3					µNS/Tomm22
µNS	14hpi	MWT14	3.363.434.348.017	336,3434348	1,6575991
		MKO14	1.873.258.021.790	187,3258022	1,12973931
		MKOplus14	2.037.056.987.800	203,7056988	0,60946682
	24hpi	MWT24	2.673.184.788.403	267,3184788	0,16577779
		MKOG24	2.603.579.188.405	260,3579188	0,18322549
		MKOGplus24	1.749.674.031.679	174,9674032	0,12278056
Tomm22	14hpi	TWT14	2.029.100.013.959	202,9100014	
		TKO14	1.658.132.990.281	165,813299	
		TKOplus14	3.342.359.120.005	334,235912	
	24hpi	TWT24	16.125.108.012.491	1612,510801	
		TKOg24	14.209.699.534.677	1420,969953	
		TKOgplus24	14.250.416.301.721	1425,04163	
		b	0.0000000000		

Summary

14hpi	R1	R2	R3	Mean	Std deviation	Std error
WT	0,434615847	0,5886065	1,6575991	1,12310282	0,534496277	0,30859157
KO	0,202217649	0,5791245	1,12973931	0,85443189	0,27530742	0,15894881
KO+	0,222487529	0,6276544	0,60946682	0,61856063	0,009093805	0,00525031

14hpi			p value
	T student	WT vs KO	0,61696481
		WT vs KO+	0,40470158
		KO vsKO+	0,65138535

24hpi	R1	R2	R3	Mean	Std deviation	Std error
WT	2,576078062	1,2242467	1,65777791	1,44101229	0,216765626	0,12514969
KO	0,119868921	1,7271569	1,83225492	1,77970592	0,052548998	0,03033918
KO+	0,004792061	1,3025229	1,22780556	1,26516421	0,037358649	0,02156903

24hpi			p value
	T student	WT vs KO	0,43861325
		WT vs KO+	0,16818485
		KO vsKO+	0,61469405

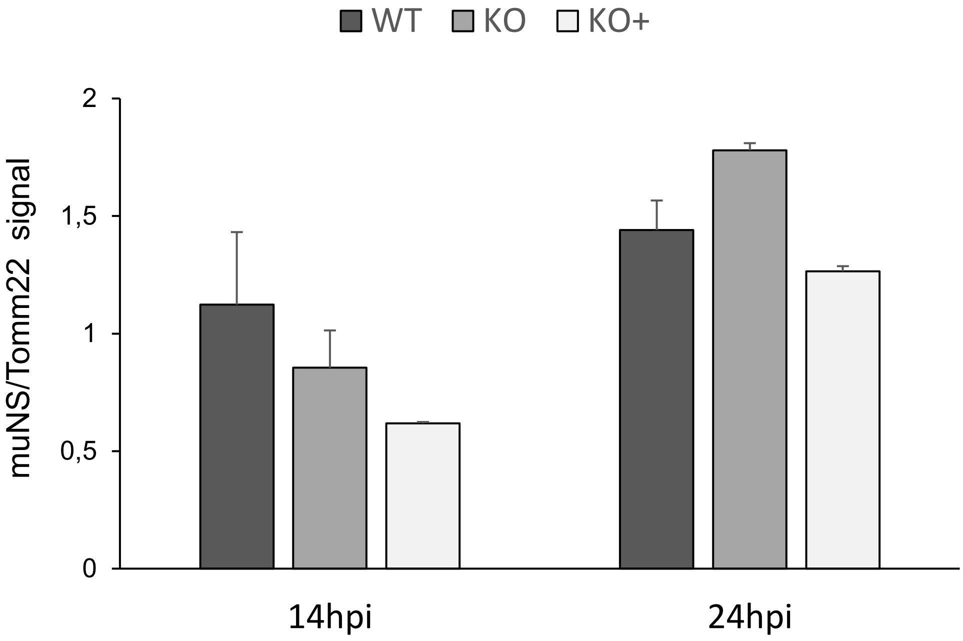

FIGURE S10C: Recomplemented cells
Extracellular titration

Extracellular Viral titer		R1	R2	R3	Mean	Std deviation	Std error
0 hpi	WT	150000	8000	14000	57333,33	80307,74143	46365,69613
	KO	350	250	150	250,00	100	57,73502692
	KO+	450	300	250	333,33	104,0833	60,09252126
14 hpi	WT	1000000	1100000	130000	743333,33	533510,3873	308022,3657
	KO	8000000	4000000	5000000	5666666,67	2081665,999	1201850,425
	KO+	1200000	270000	600000	690000,00	471487,0094	272213,1518
24 hpi	WT	50000000	45000000	30500000	41833333,33	10128343,07	5847601,598
	KO	170000000	250000000	40000000	153333333,33	105987420,6	61191865,84
	KO+	30000000	20000000	4000000	18000000,00	13114877,05	7571877,794

Extracellular Titration			p value
T stud	14hpi	WT vs KO	0,047242
		WT vs KO+	0,903122
		KO vs KO+	0,047632
	24hpi	WT vs KO	0,209101
		WT vs KO+	0,071392
		KO vs KO+	0,071392

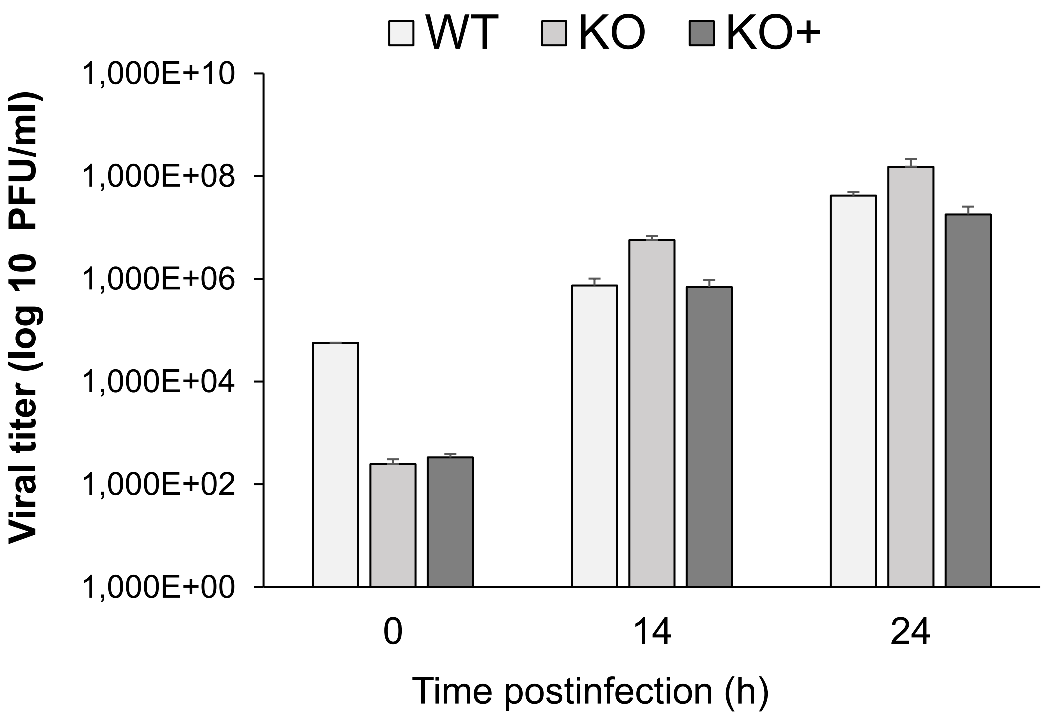

FIGURE S10D: Recomplemented cells
Intracellular titration

Intracellular Viral titer		R1	R2	R3	Mean	Std deviation	Std error
0 hpi	WT	8400	46500	21800	25566,67	19327,26916	11158,6041
	KO	1150	350	350	616,67	461,8802154	266,666667
	KO+	450	400	700	516,67	160,7275127	92,7960727
14 hpi	WT	45000	450000	1500000	665000,00	750949,3991	433560,838
	KO	125000	550000	200000	291666,67	226844,2931	130968,614
	KO+	335000	400000	3000000	1245000,00	1520222,023	877700,594
24 hpi	WT	550000	27500000	6000000	11350000,00	14249298,23	8226836,17
	KO	410000	30500000	19000000	16636666,67	15183577,75	8766242,7
	KO+	500000	2500000	1300000	1433333,33	1006644,591	581186,526

Extracellular Titration			p value
T student	14hpi	WT vs KO	0,484691182
		WT vs KO+	0,596257516
		KO vs KO+	0,39099539
	24hpi	WT vs KO	0,682919947
		WT vs KO+	0,351215189
		KO vs KO+	0,224590068

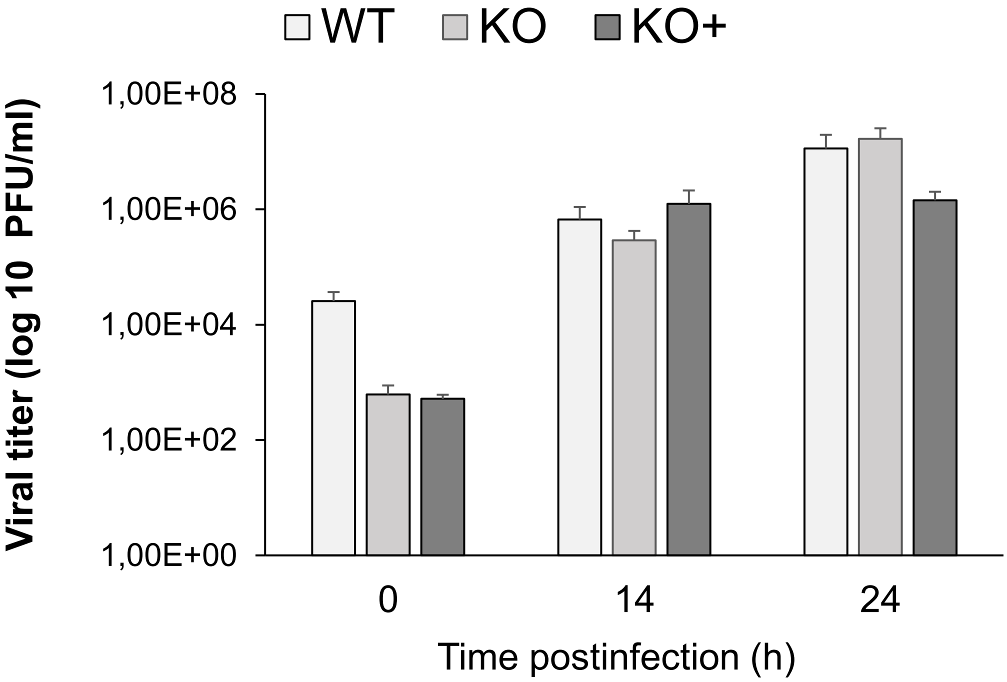

Supplement: File S1 — Raw data corresponding to graphs. [file jvi.01389-25-s0001.pdf]
