# Supplementary material for: Annexin A2 stabilizes the endoplasmic reticulum and actin cytoskeleton and influences the formation of reovirus factories
Source: J Virol. 2025 Nov 24;99(12):e01389-25. doi: 10.1128/jvi.01389-25 (PMC12724372; doi:10.1128/jvi.01389-25)
Supplement: Supplemental figures — Figures S1 to S10. [file jvi.01389-25-s0002.pdf]

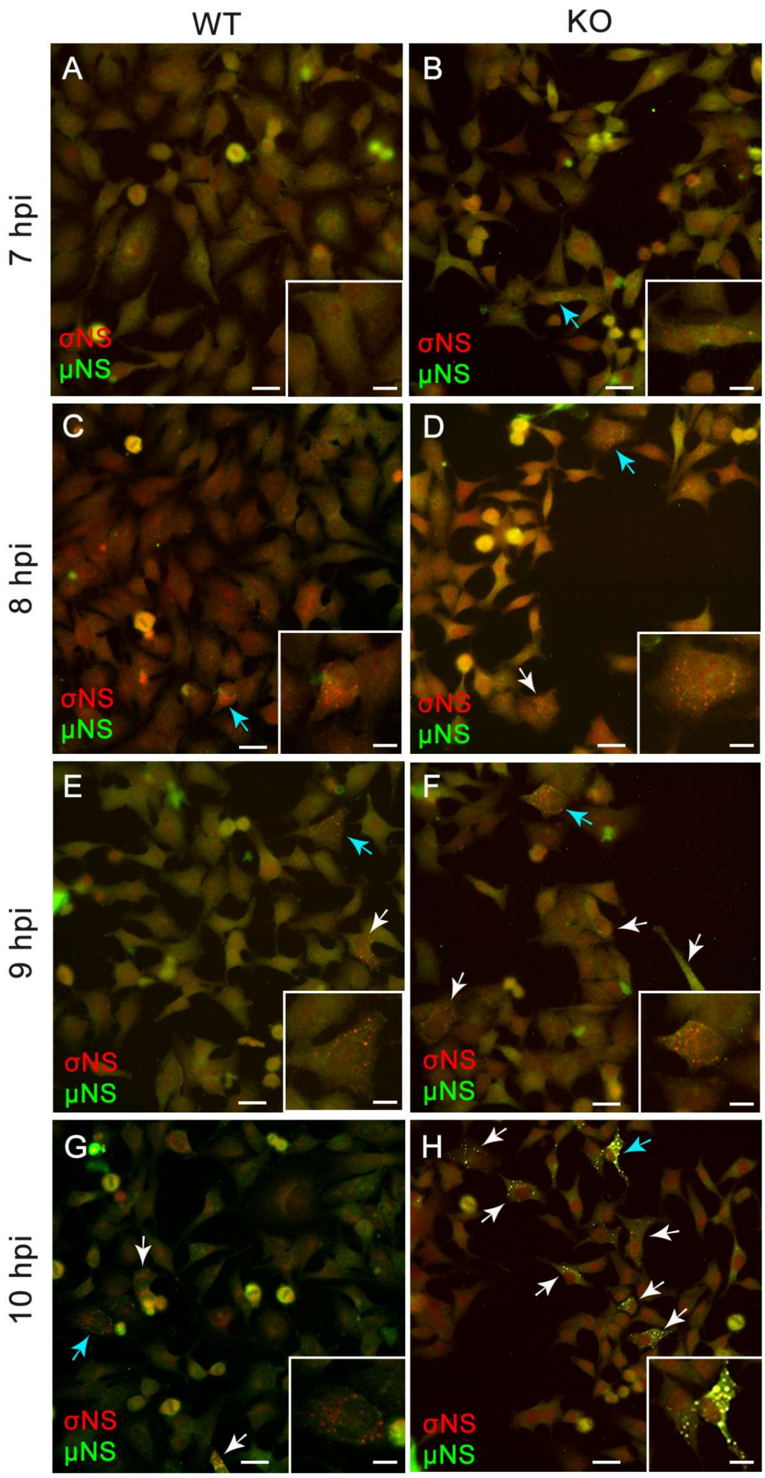
WT
KO
7 hpi
8 hpi
9 hpi
10 hpi
A
B
C
D
E
F
G
H
σNS
μNS

FIG S1 Effects of absence of ANXA2 on infection progression at early times post-infection. (A-H) WT and ANXA2 KO HeLa cells were infected with reovirus, fixed at 7, 8, 9, and 10 h post-adsorption, stained with µNS-specific and σNS-specific antibodies, and imaged by fluorescence microscopy. Cells with VFs (arrows) were more abundant in infected-KO HeLa cells than in infected-WT HeLa cells. Regions corresponding to high-magnification insets are indicated by blue arrow. Bars, 25 µm (Insets, 10 µm).

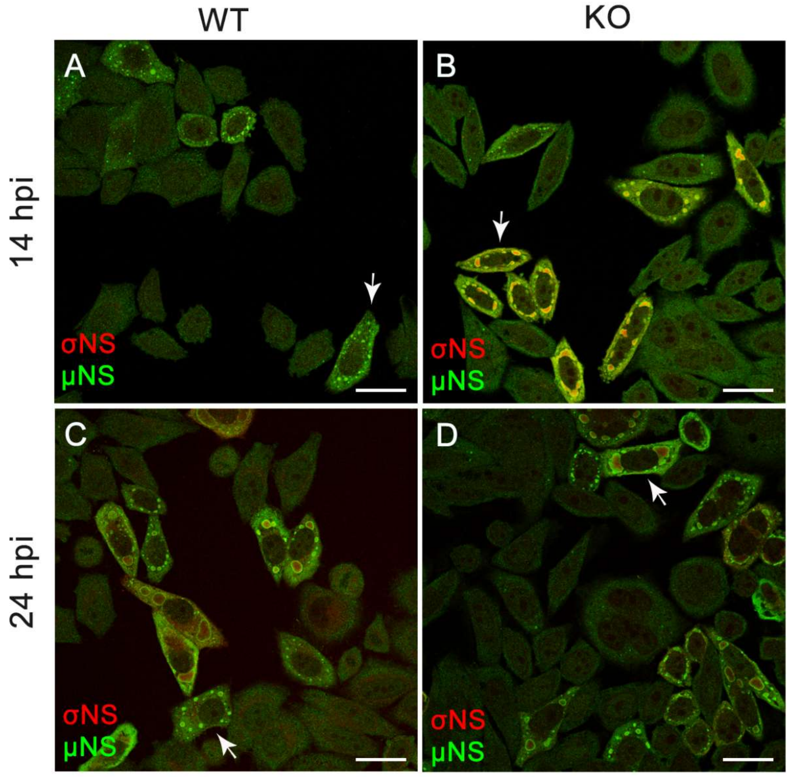

FIG S2 Effects of absence of ANXA2 on infection progression at 14 and 24 h post-adsorption. (A-C) WT and ANXA2 KO HeLa cells were infected with reovirus, processed for immunostaining with μNS and σNS-specific antibodies and imaged by fluorescence microscopy. At 14 and 24 h post-adsorption, cells with VFs are more abundant in KO HeLa cells compared to WT HeLa cells. Bars, 25 μm.

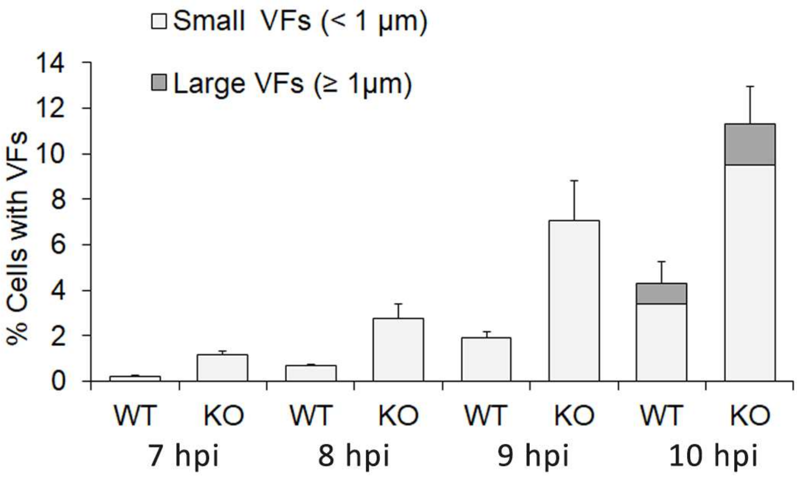

FIG S3 The absence of ANXA2 does not affect VF size at early times post-infection. WT and ANXA2 KO HeLa cells were infected with reovirus and fixed at 7, 8, 9, and 10 h post-adsorption. The percentage of cells with small VFs (less than 1 µm of length) or large VFs (1 µm or larger) was quantified by immunostaining with a µNS-specific antibody. At 10 h post-adsorption, in WT and KO cells, some large VFs are visible, but no significant differences in size were observed. Results are presented as the mean of three independent experiments.

A

WT	Pearson's	Mander's 1	Mander's 2
WT1	0.865	0.851	0.931
WT2	0.874	0.904	0.851
WT3	0.858	0.853	0.907
Average	0.866	0.869	0.896
**KO**	**Pearson's**	**Mander's 1**	**Mander's 2**
KO1	0.807	0.622	0.696
KO2	0.815	0.712	0.650
KO3	0.786	0.7	0.669
Average	0.803	0.678	0.672

B

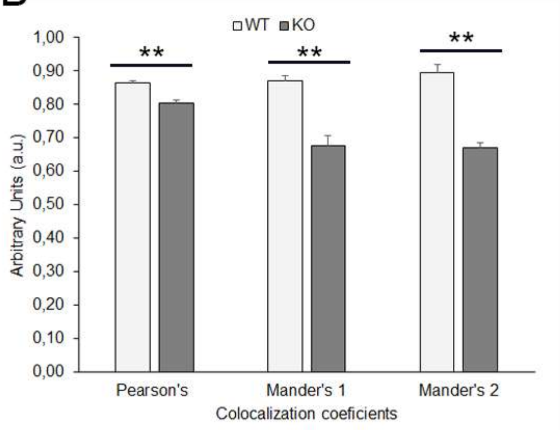

FIG S4 Effects of lack of ANXA2 in σNS and μNS colocalization. WT and ANXA2 KO HeLa cells were infected with reovirus. At 14 h post-adsorption, they were processed for immunostaining with μNS and σNS-specific antibodies and imaged by confocal microscopy. Colocalization of σNS and μNS in viral factories was quantified by Pearson's and Mander's colocalization coefficients. (A) Coefficients obtained from VFs in the presence or absence of ANXA2. WT 1 to 3 and KO 1 to 3 are replicates of the WT and ANXA2 KO experimental conditions, respectively. Twelve VFs from each replicate were studied. Mander's 1 represents the fraction of red signal (σNS) overlapping green signal (μNS) and Mander's 2 the fraction

of green signal (µNS) overlapping red signal (σNS). (B) Comparison of Pearson and Mander’s coefficients in these two conditions. Results are the mean of three independent experiments. Unpaired two-tailed Student’s *t*-test. **, $P < 0.01$.

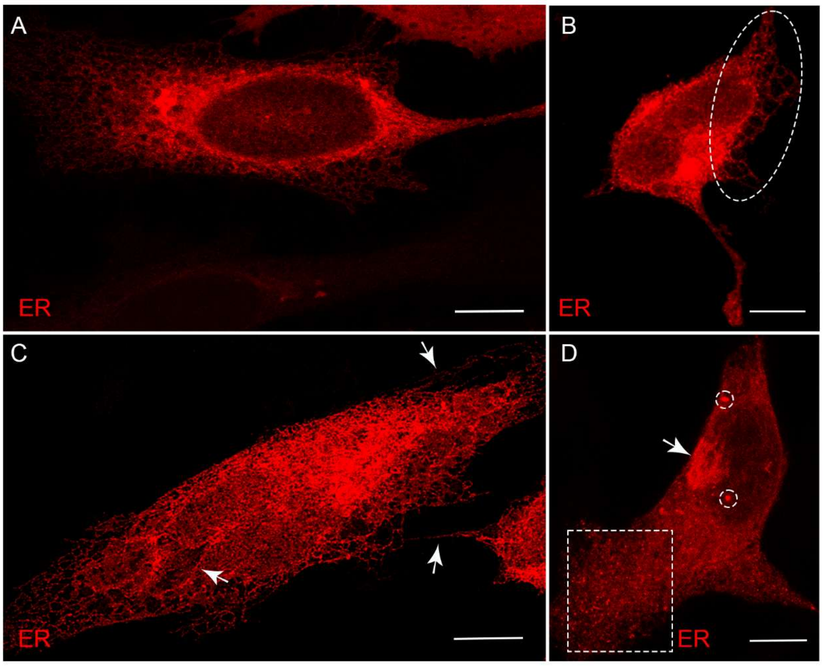

FIG S5 Representative images of ER morphologies. WT and ANXA2 KO HeLa cells were transfected with plasmid encoding mCherry-ER-3, incubated for 24 h, and mock-infected or infected with reovirus, Cells were fixed at 14 or 24 h post-adsorption and imaged using confocal immunofluorescence microscopy and deconvolution image processing. (A) Normal ER. (B) Stretched ER (dashed oval). (C) Unbranched ER (arrows). (D) Large collapsed ER (arrow), small collapsed ER (dashed circles), fragmented ER (dashed square). Bars, 10 μm.

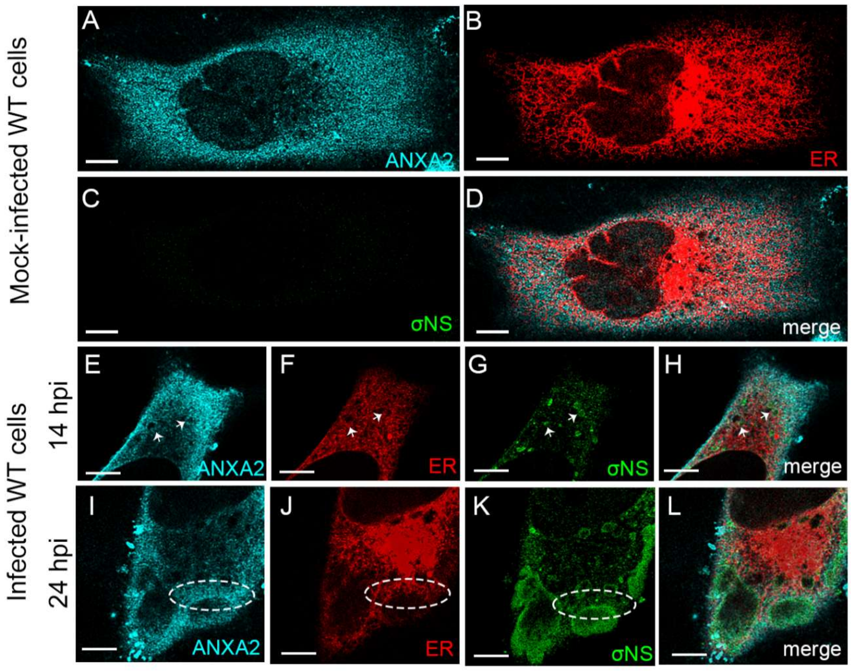

FIG S6 Localization of ANXA2 and the ER in infected HeLa cells. WT HeLa cells were transfected with plasmid encoding mCherry-ER-3, incubated for 24 h, and either mock-infected or infected with reovirus. At 14 and 24 h post-adsorption, cells were fixed and imaged using confocal immunofluorescence microscopy followed by deconvolution image processing. (A-D) In Mock infected cells, ANXA2 and ER do not colocalize. (E-H) At 14 h post-adsorption, some small VFs are surrounded by ANXA2 and ER (arrows). (I-L) At 24 h post-adsorption, ANXA2 is recruited around-large VFs and the ER signal with little colocalization between them (dashed ovals). Bars, 5 μm.

A

	ER MORPHOLOGY			
	Stretched ER	Small ER collapses	Fragmented ER	Large ER collapses
σNS-transfected WT cells	15 / 93.33%	6 / 37.77%	4 /25.55%	2 / 11.11%
σNS-transfected KO cells	1 / 5.55%	10 /83.33%	1 /11.11%	0 /0%
µNS-transfected WT cells	1 / 3.70%	10 / 61.90%	12 / 92.59%	2 / 13.09%
µNS-transfected KO cells	0 /0%	8 / 42.69%	10 / 53.80%	16 / 88.57%
µNS + σNS transfected WT cells	6 / 48.33%	9 / 70%	8 / 63.33%	3 / 23.33%
µNS + σNS transfected KO cells	1 / 6.67%	12 / 86.67%	6 / 43.33%	8 / 58,33%

B

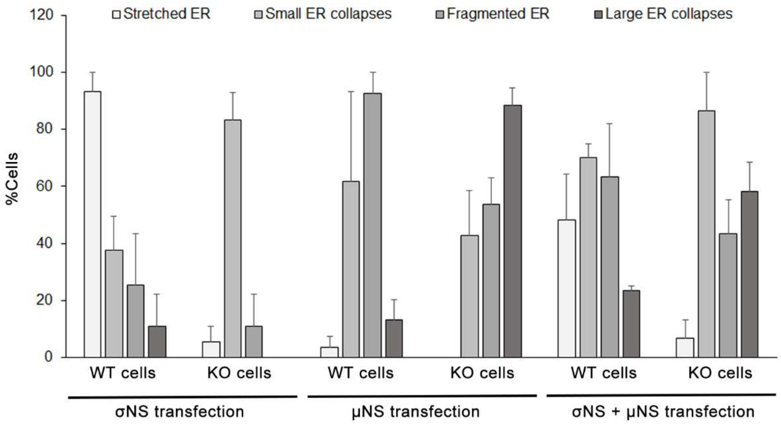

FIG S7 Quantification of ER morphology from Hela cells transfected with plasmids encoding reovirus nonstructural proteins. WT and ANXA2 KO HeLa cells were either transfected with plasmids encoding σNS, µNS or both, incubated for 24 h, fixed, and imaged using confocal immunofluorescence microscopy and deconvolution image processing (A) Number and percentage of cells with the most common ER remodeling features observed under these conditions: stretched ER, fragmented ER tubules, small collapsed ER (less than 6 µm), and large collapsed ER (6 µm or larger) in mock-infected or reovirus-infected WT or ANXA2 KO cells. One cell could have more than a single condition. Normal ER

is not observed under these conditions. (B) Comparison of ER morphologies in these four conditions. Results are the mean of three independent experiments.

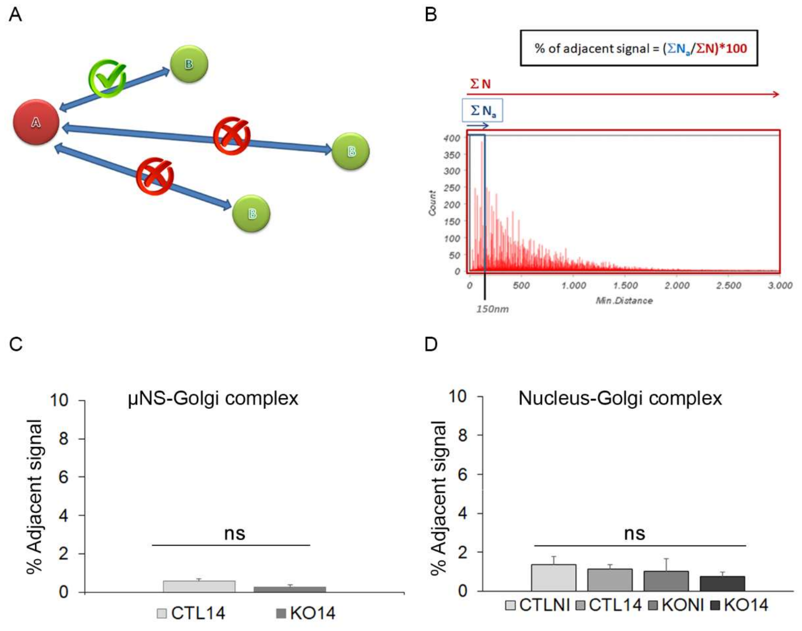

FIG S8 The minimum distance quantification method and its biological controls. (A) The minimum distances method measures distances from each fluorescent signal in channel A to each fluorescent signal in channel B and records the shortest distances. (B) The measurements obtained are ordered by length. We obtained the percent adjacent signal by dividing the sum of measurements designated as adjacent signal (from 0 to 150 nm) by the sum of the total measurements obtained between those two channels. The biological test of the method was conducted by measuring the minimum distances between the Golgi and (C) reovirus µNS or (D) the nucleus. The percent adjacent signal between these two signals was calculated and compared. The results are presented as

the mean of nine images analyzed for each condition. Unpaired two-tailed Student’s t-test. ns, non-significant.

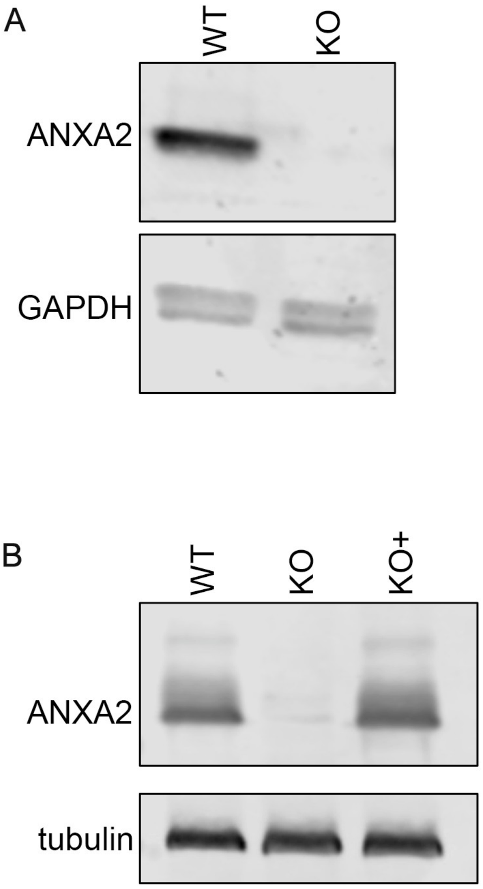

FIG S9 ANXA2 expression in WT/ KO and WT/KO/KO+ cell lines. Lysates of (A) WT and KO HeLa cells from the Kast lab and (B) rederived WT, KO, and KO+ HeLa cells were subjected to electrophoresis and immunoblotting using an ANXA2 antiserum. GAPDH and tubulin were used as loading controls. A representative immunoblot is shown. (A, B) In both KO cells lines ANXA2

expression is absent. (B) In the ANXA2 KO+ cell line ANXA2 expression is restored.

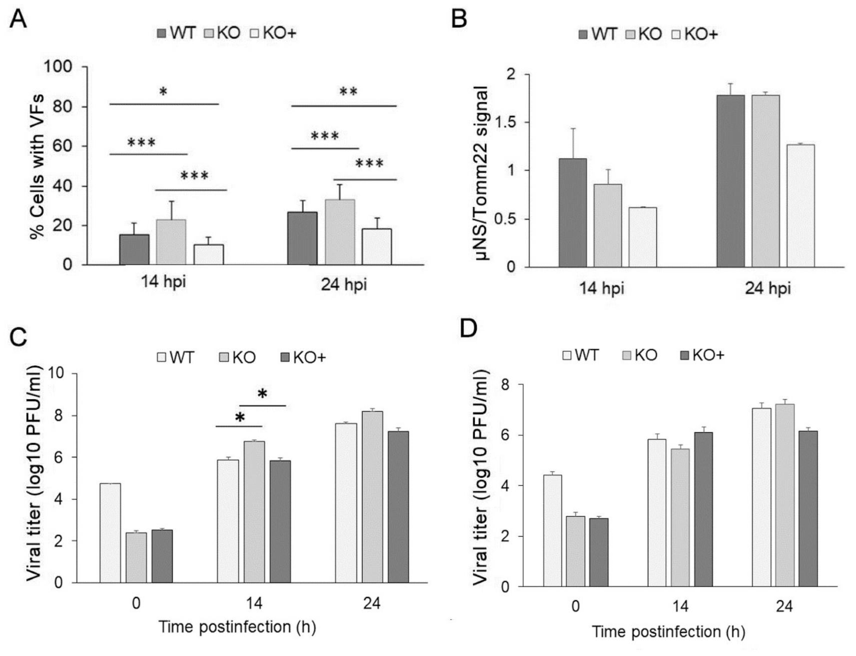

FIG S10 Complementation of ANXA2 expression in rederived KO cells with plasmid encoding WT ANXA2 (KO+) restores VF formation kinetics. (A-D) Rederived WT, ANXA2 KO, and ANXA2 KO+ HeLa cells were infected with reovirus and fixed at 14 and 24 h post-adsorption. (A) Percentage of infected cells detected by immunostaining with a μNS-specific antibody. (B) Reovirus μNS protein levels at 14 and 24 h post-adsorption determined by immunoblotting using a μNS-specific antibody and normalized to Tomm22. (C and D) Reovirus titers at 0, 14, and 24 h post-adsorption in (C) culture supernatants and (D) cell lysates determined by plaque assay. Results are the mean of three independent

experiments. Unpaired two-tailed Student's *t*-test. *, $P < 0.05$; **, $P < 0.01$; *** $P < 0.001$.

Suppl. Video Legends

VIDEO S1: 3D representation of mock-infected WT HeLa cells. Actin labelled (blue).

VIDEO S2: 3D representation of infected WT HeLa cells (14 h post-adsorption). Actin (blue) and σNS (green) labelled.

VIDEO S3: 3D representation of mock-infected ANXA2 KO HeLa cells. Actin labelled (blue).

VIDEO S4: 3D representation of infected ANXA2 KO HeLa cells (14 h post-adsorption). Actin (blue) and σNS (green) labelled.

VIDEO S5: 3D representation of mock-infected WT HeLa cells. Actin (blue) and the ER (red) labelled

VIDEO S6: 3D representation of infected WT HeLa cells (14 h post-adsorption). Actin (blue), the ER (red) and σNS (green) labelled.

VIDEO S7: 3D representation of mock-infected ANXA2 KO HeLa cells. Actin labelled (blue) and the ER (red).

VIDEO S8: 3D representation of infected ANXA2 KO HeLa cells (14 h post-adsorption). Actin (blue), the ER (red) and σNS (green) labelled.

Con formato: Inglés (Estados Unidos)
